# Supplementary material for: PLA2G16 expression predicts prognosis and gemcitabine sensitivity in patients with pancreatic cancer
Source: PeerJ. 2025 May 30;13:e19517. doi: 10.7717/peerj.19517 (PMC12129006; doi:10.7717/peerj.19517)
Supplement: Supplemental Information 1 [file peerj-13-19517-s001.docx]

| **Reagent Name** | **Company** | **Catalog Number** |
| --- | --- | --- |
| Gemcitabine | Med Chem Express | HY-17026 |
| RPMI-1640 Medium | Shanghai Yuanpei | L210KJ |
| High-Glucose DMEM Medium | Shanghai Yuanpei | L110KJ |
| Phosphate Buffered Saline (PBS) | VisTech | 06150925 |
| Fetal Bovine Serum (FBS) | VisTech | SE100-011 |
| Trypsin | Shanghai Yuanpei | S310KJ |
| Methanol | Sinopharm | 10014118 |
| 4% Paraformaldehyde (PFA) Fixative | Biosharp | BL539A |
| Crystal Violet Stain | Beyotime Biotechnology | C0121 |
| 5× Protein Loading Buffer | Beyotime Biotechnology | P0015 |
| BCA Protein Assay Kit | Beyotime Biotechnology | P0012 |
| Absolute Ethanol | Sinopharm | 10009218 |
| Tris-Glycine Transfer Buffer | Cowin Biotech | CW0044 |
| Phosphatase Inhibitor Cocktail | Coolaber | SL33213350 |
| Cell Counting Kit-8 (CCK-8) | Meilunbio | MA0218 |
| SDS-PAGE Running Buffer | Cowin Biotech | CW0045 |
| PMSF | Beyotime Biotechnology | ST506 |
| 20× TBS Buffer | Meilunbio | MA0141 |
| RIPA Lysis Buffer | Beyotime Biotechnology | P0031 |
| DMSO | Solarbio | D8371 |
| Antibody Dilution Buffer | Beyotime Biotechnology | P0256 |
| 0.22 μm PVDF Membrane | Millipore | ISEQ10100 |
| ECL Chemiluminescent Substrate | Meilunbio | MA0186 |
| Fast PAGE Gel Preparation Kit | Yeasen Biotechnology | PG112 |
| Anti-mouse IgG HRP | Cell Signaling Technology | 7076 |
| Anti-PLA2G16 Antibody | Abclonal | A16018 |
| Anti-β-actin Antibody | Abclonal | AC038 |
| Reverse Transcription Kit | Vazyme | R323 |
| qPCR Master Mix | Vazyme | Q711 |
| Anti-GAPDH Antibody | Cell Signaling Technology | 5174 |
| OPTI-MEM Medium | Senrui Biotech | CR22600 |
| Lipofectamine 2000 | Vazyme | TL201-01 |
| Chloroform | Sinopharm | CFSR-10006818 |

**Table S1.** Experimental reagents.
