# Supplementary material for: PLA2G16 expression predicts prognosis and gemcitabine sensitivity in patients with pancreatic cancer
Source: PeerJ. 2025 May 30;13:e19517. doi: 10.7717/peerj.19517 (PMC12129006; doi:10.7717/peerj.19517)
Supplement: Supplemental Information 2 [file peerj-13-19517-s002.docx]

| **Name (Abbreviation)** | **Company** | **Model Number** |
| --- | --- | --- |
| Biological Safety Cabinet | Thermo Scientific | 1389 |
| CO₂ Incubator | Thermo Scientific | HERAcell 150i |
| Multifunctional Microplate Reader | Thermo Scientific | N12639-02 |
| Vertical Electrophoresis & Transfer System | Bio-Rad, USA | 1658033 |
| Inverted Fluorescence Microscope | Nikon, Japan | 405503 |
| NanoDrop 2000 Spectrophotometer | Thermo Fisher, USA | ND-ONE-W |
| Real-Time PCR System | Bio-Rad, USA | 621BR44524 |
| -80°C Ultra-Low Freezer | Thermo Scientific | ULT1490 |
| High-Speed Refrigerated Centrifuge | Thermo Fisher, USA | ST 16R |
| Analytical Balance (0.01 mg) | ADAM, UK | NBL 214e |
| Benchtop High-Speed Centrifuge | Eppendorf, Germany | 5428HQ427254 |
| Micro Ultrasonic Cell Disruptor | Diagenode, Belgium | B01020001 |
| Flow Cytometer | Beckman Coulter, USA | BB12038 |
| Micropipettes | Eppendorf, Germany | 3120000 |
| Vortex Mixer | Kylin-Bell | Vortex-6 |
| Chemiluminescence Imaging System | Bio-Rad, USA | 733BR2073 |

**Table S2.** Experimental instruments.
