# Supplementary material for: PLA2G16 expression predicts prognosis and gemcitabine sensitivity in patients with pancreatic cancer
Source: PeerJ. 2025 May 30;13:e19517. doi: 10.7717/peerj.19517 (PMC12129006; doi:10.7717/peerj.19517)
Supplement: Supplemental Information 3 — PZF/PZFX files must be opened using GraphPad Prism. [file peerj-13-19517-s003.zip › FIG 1/FIG 1E.pdf]

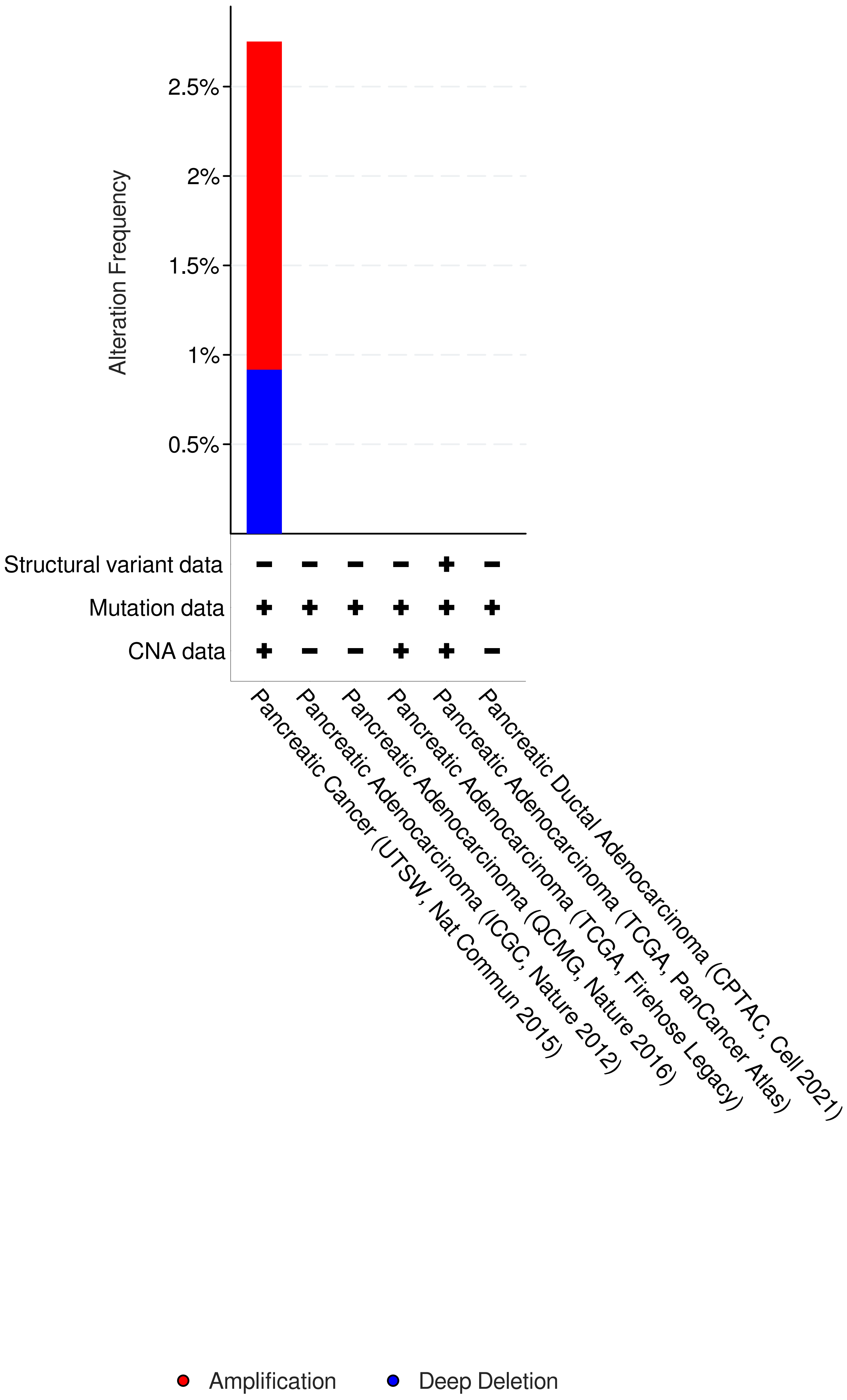

Alteration Frequency
2.5%
2%
1.5%
1%
0.5%
Structural variant data
Mutation data
CNA data
Pancreatic Cancer (UTSW, Nat Commun 2015)
Pancreatic Adenocarcinoma (ICGC, Nature 2012)
Pancreatic Adenocarcinoma (QCMG, Nature 2016)
Pancreatic Adenocarcinoma (TCGA, Firehose Legacy)
Pancreatic Adenocarcinoma (TCGA, PanCancer Atlas)
Pancreatic Ductal Adenocarcinoma (CPTAC, Cell 2021)
Amplification
Deep Deletion
