# Supplementary figures and images for: PLA2G16 expression predicts prognosis and gemcitabine sensitivity in patients with pancreatic cancer
Source: PeerJ. 2025 May 30;13:e19517. doi: 10.7717/peerj.19517 (PMC12129006; doi:10.7717/peerj.19517)

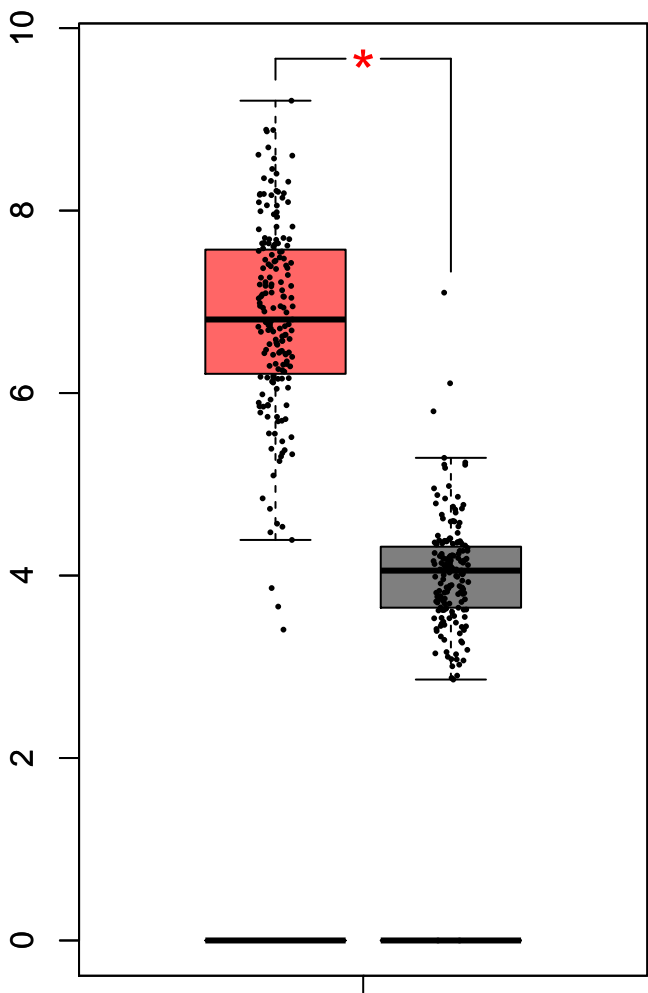

PAAD  
(num(T)=179; num(N)=171)

Supplement: Supplemental Information 3 — PZF/PZFX files must be opened using GraphPad Prism. [file peerj-13-19517-s003.zip › FIG 1/FIG 1A.pdf]

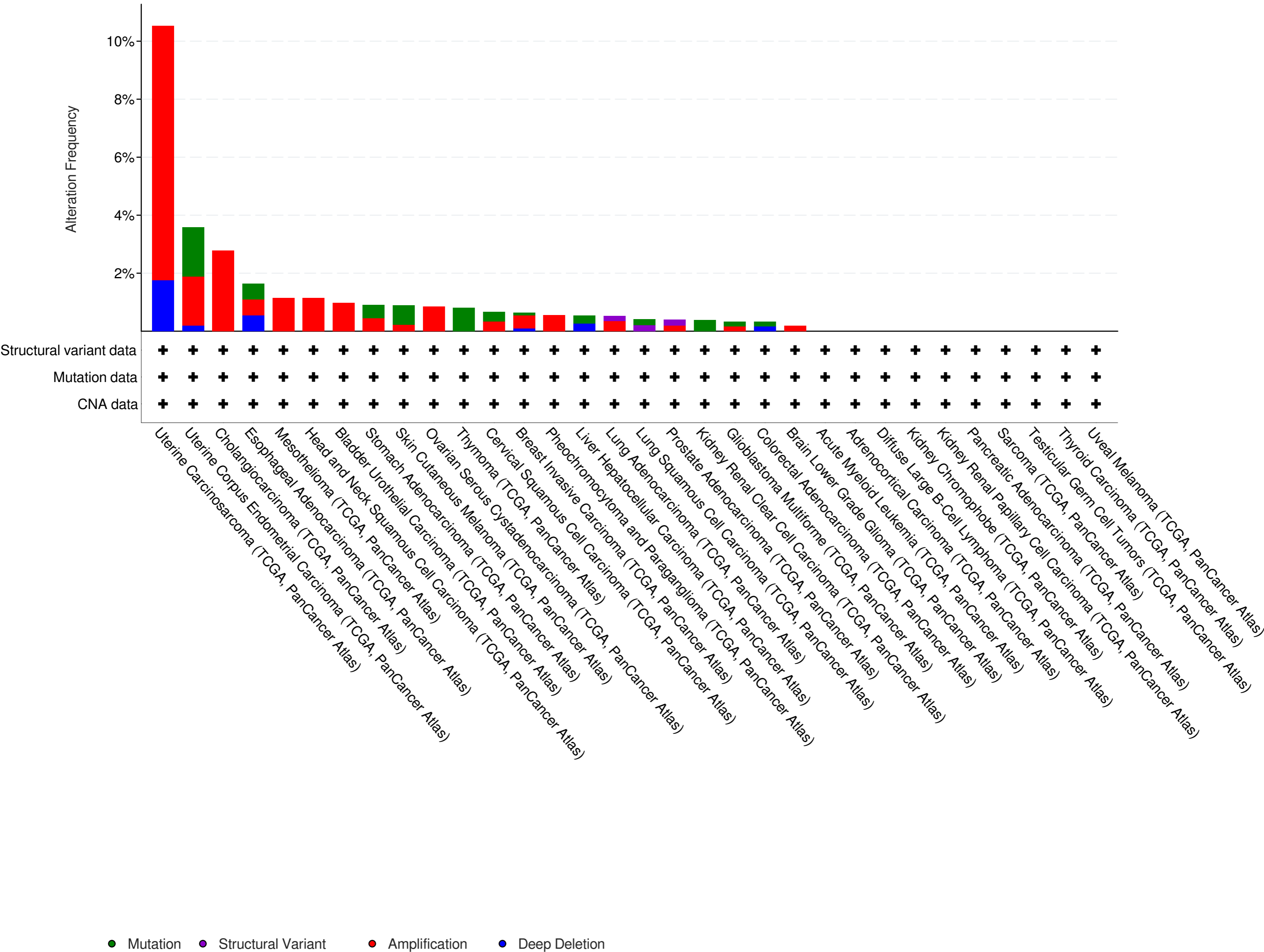

Supplement: Supplemental Information 3 — PZF/PZFX files must be opened using GraphPad Prism. [file peerj-13-19517-s003.zip › FIG 1/FIG 1D.pdf]

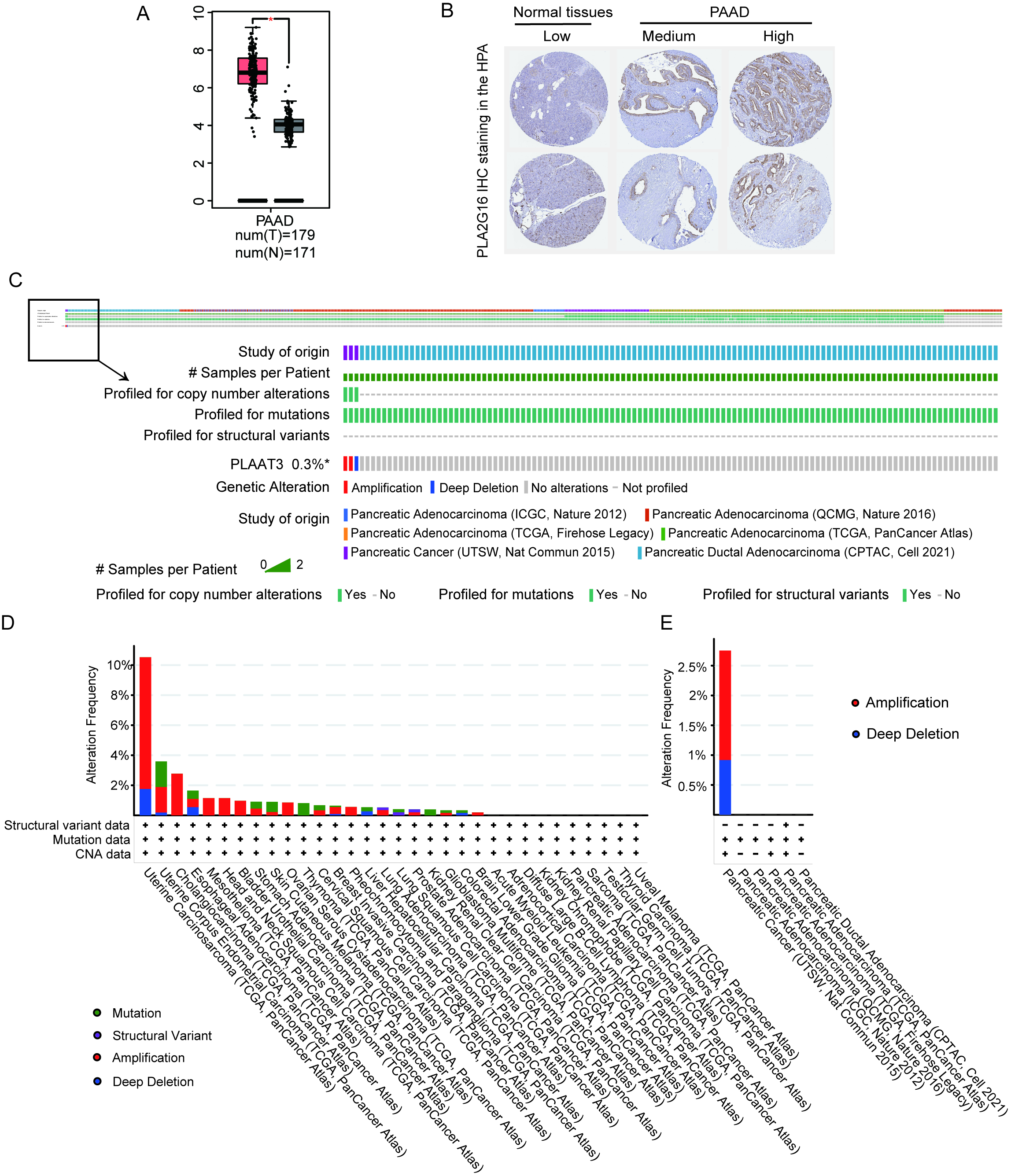

Supplement: Supplemental Information 3 — PZF/PZFX files must be opened using GraphPad Prism. [file peerj-13-19517-s003.zip › FIG 1/Figure_1.tif]

# Hazard ratio

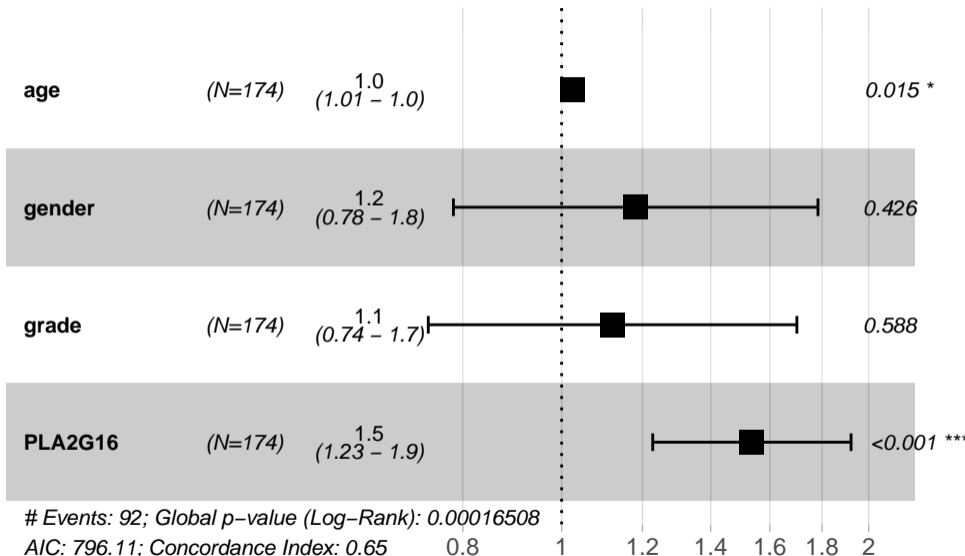

Supplement: Supplemental Information 4 — PZF/PZFX files must be opened using GraphPad Prism. [file peerj-13-19517-s004.zip › FIG 2/FIG 2A/FIG 2A.pdf]

# Overall Survival

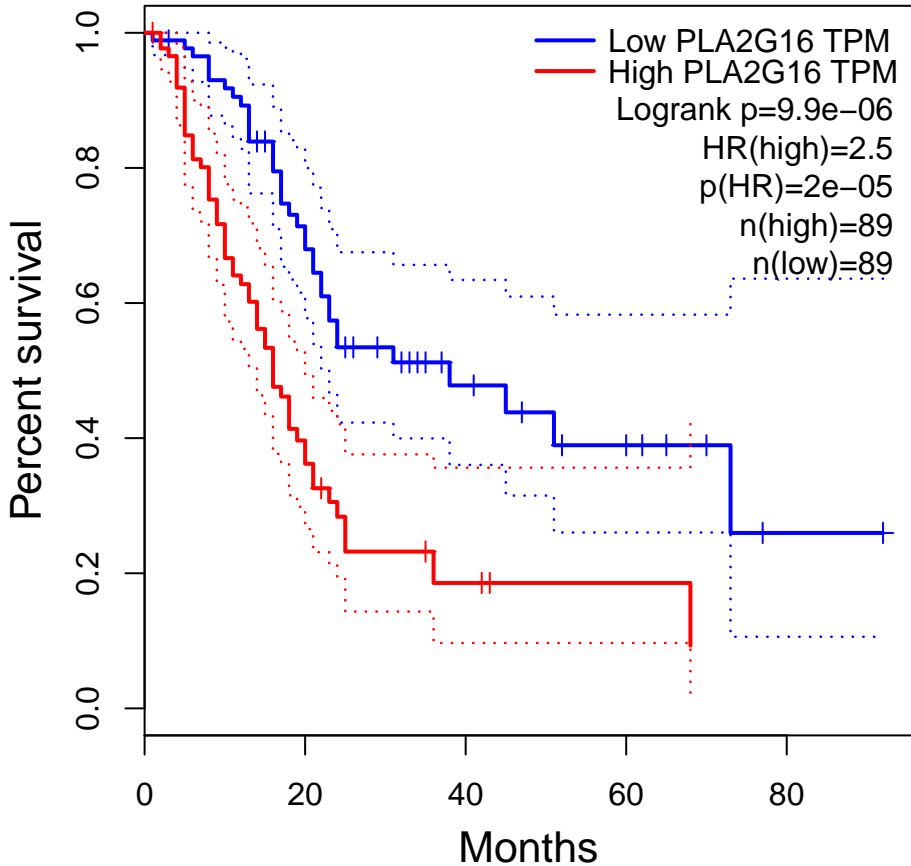

Supplement: Supplemental Information 4 — PZF/PZFX files must be opened using GraphPad Prism. [file peerj-13-19517-s004.zip › FIG 2/FIG 2B.pdf]

# Disease Free Survival

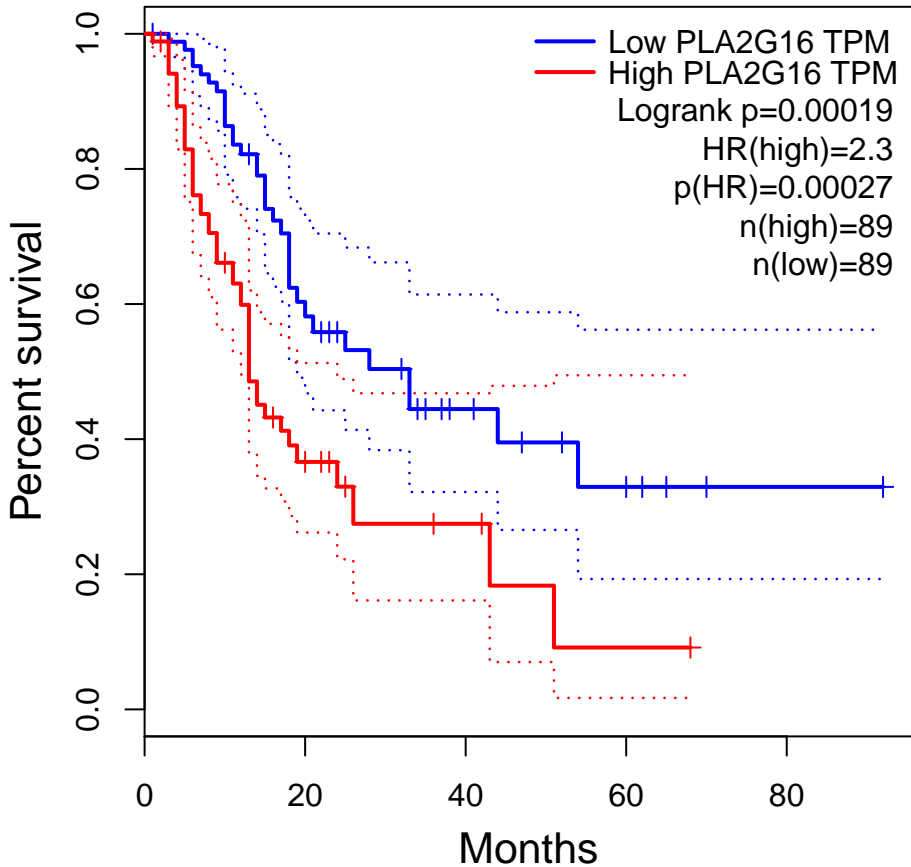

Supplement: Supplemental Information 4 — PZF/PZFX files must be opened using GraphPad Prism. [file peerj-13-19517-s004.zip › FIG 2/FIG 2C.pdf]

# Overall Survival

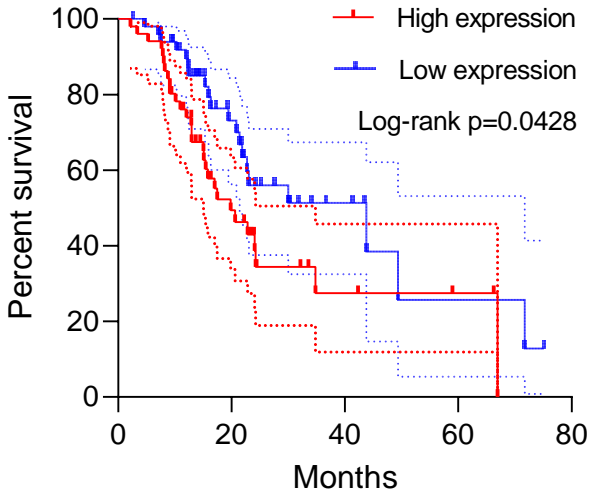

Supplement: Supplemental Information 4 — PZF/PZFX files must be opened using GraphPad Prism. [file peerj-13-19517-s004.zip › FIG 2/FIG 2D/FIG 2D.pdf]

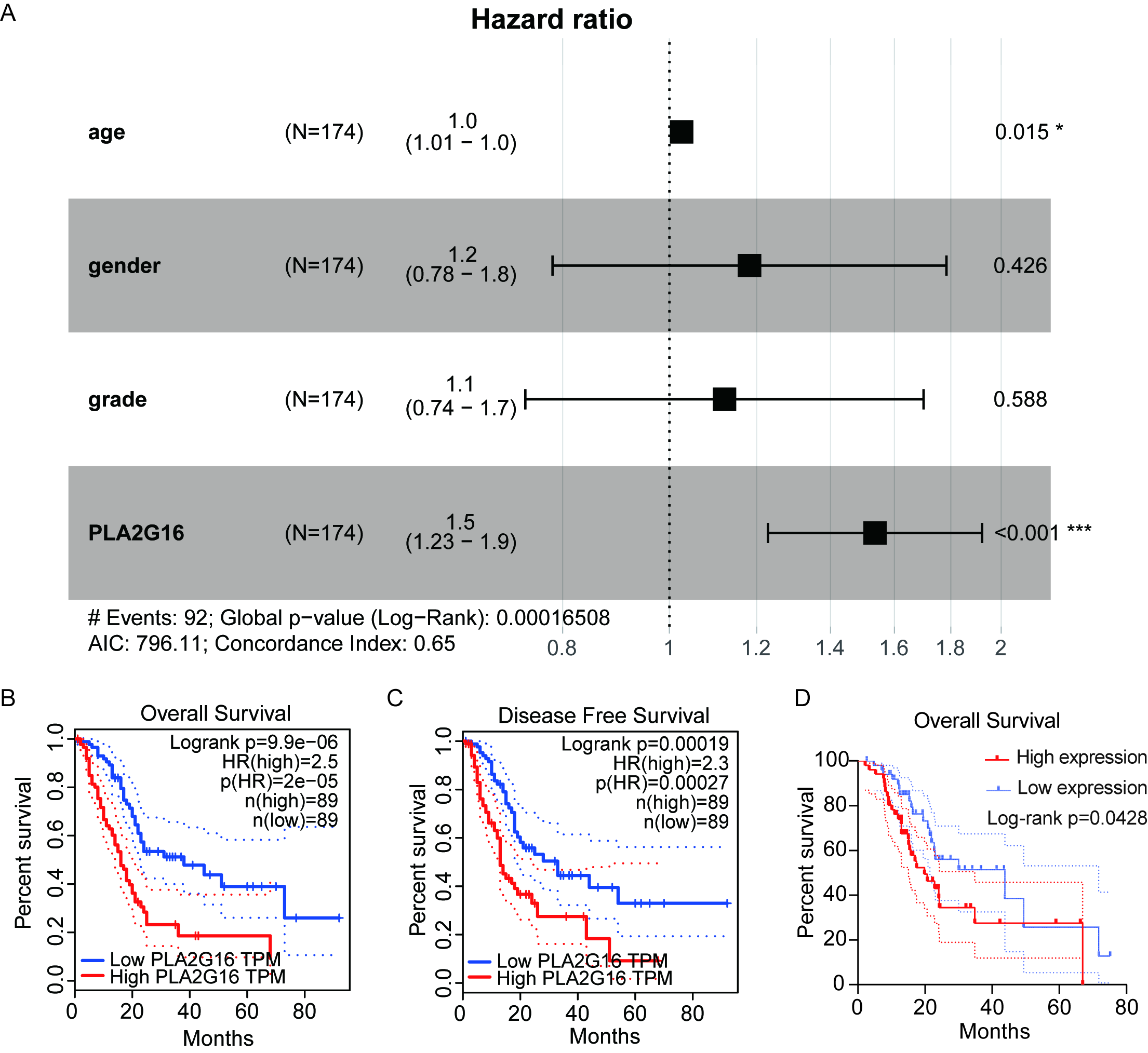

Supplement: Supplemental Information 4 — PZF/PZFX files must be opened using GraphPad Prism. [file peerj-13-19517-s004.zip › FIG 2/FIG2.tif]

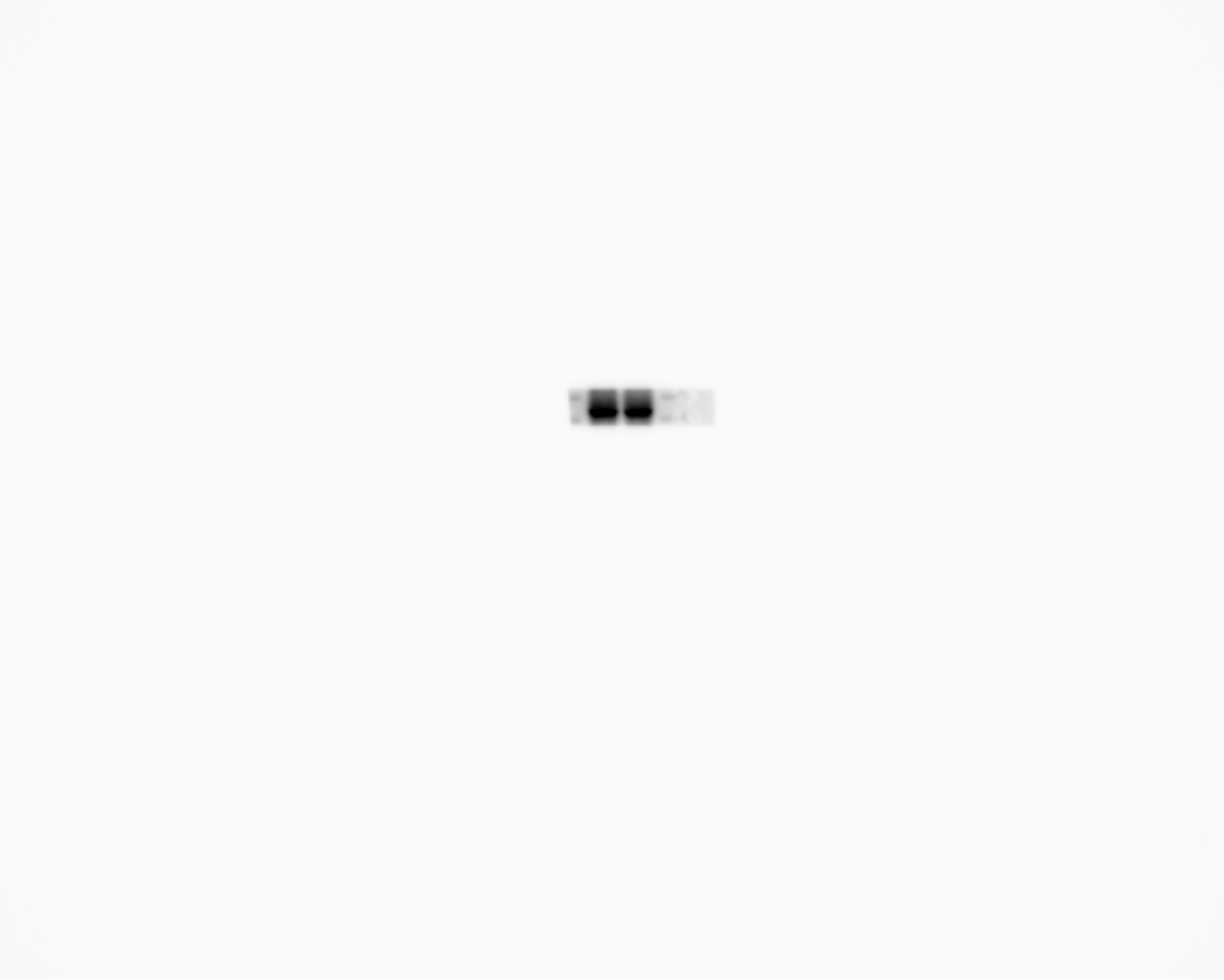

Supplement: Supplemental Information 5 — PZF/PZFX files must be opened using GraphPad Prism. [file peerj-13-19517-s005.zip › FIG _3B-I/FIG 3D/bactin-bxpc-3-250219_1(Chemiluminescence).tif]

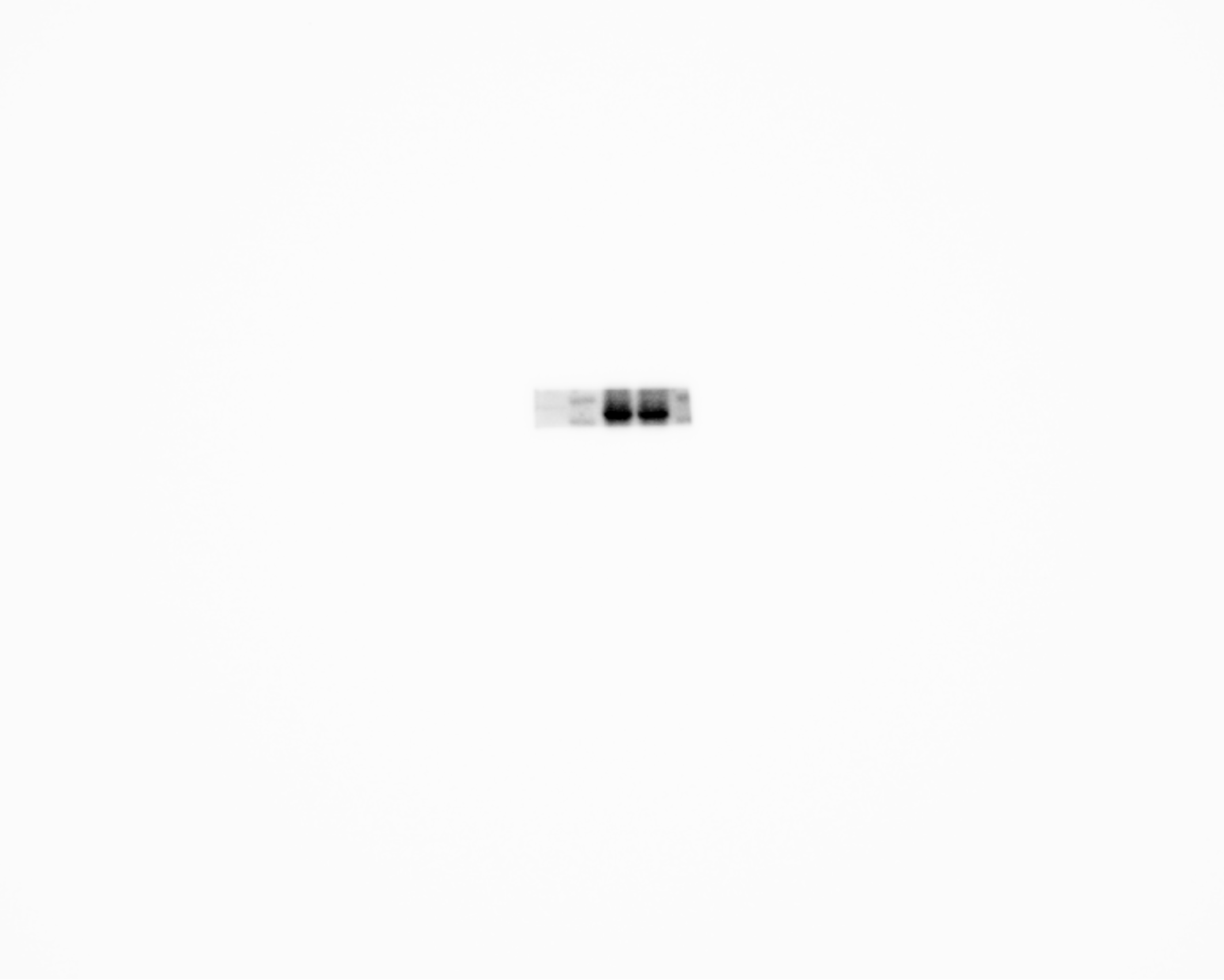

Supplement: Supplemental Information 5 — PZF/PZFX files must be opened using GraphPad Prism. [file peerj-13-19517-s005.zip › FIG _3B-I/FIG 3D/bactin-panc-1-250219_1(Chemiluminescence).tif]

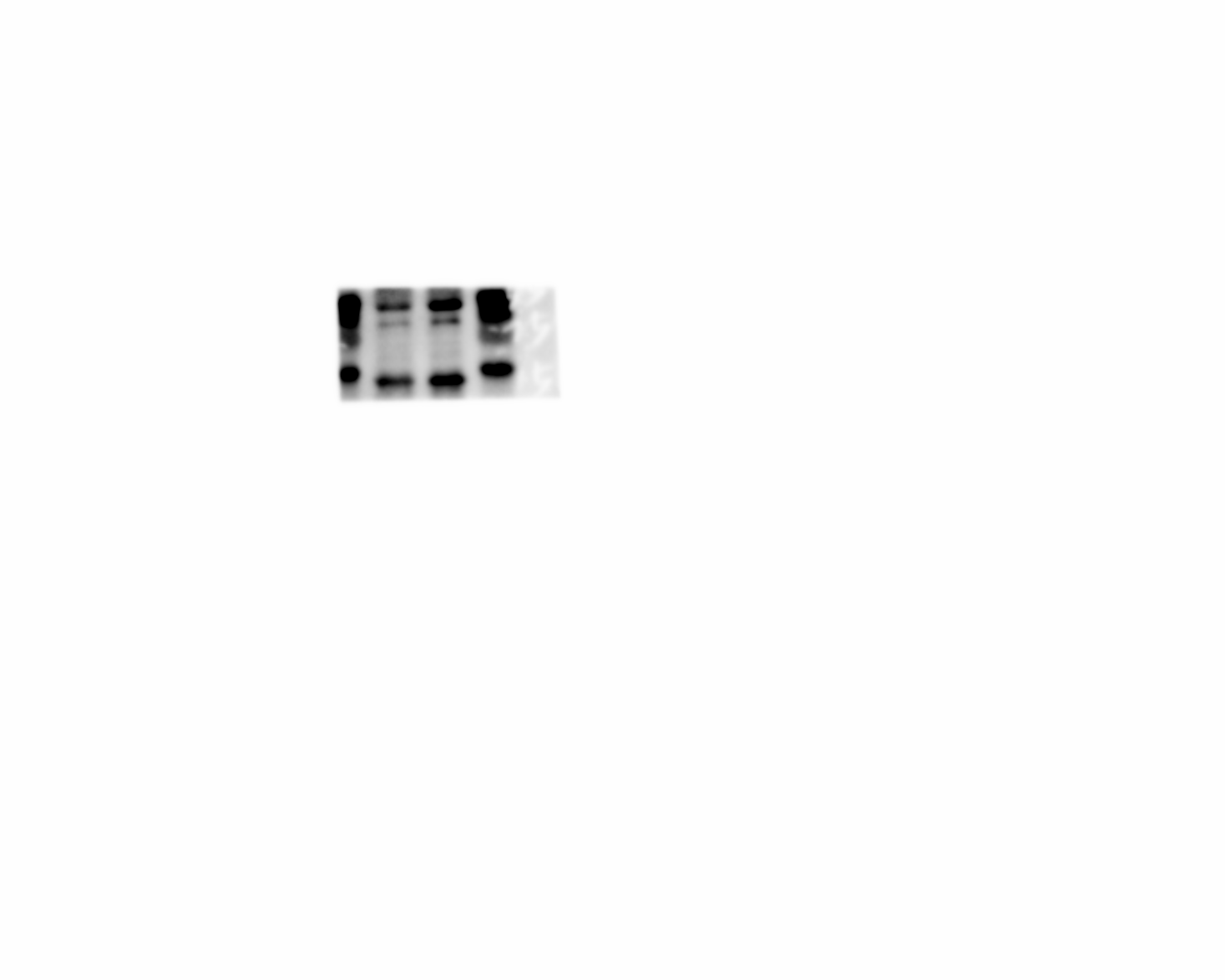

Supplement: Supplemental Information 5 — PZF/PZFX files must be opened using GraphPad Prism. [file peerj-13-19517-s005.zip › FIG _3B-I/FIG 3D/PLA2G16-BXPC-3-250219_1(Chemiluminescence).tif]

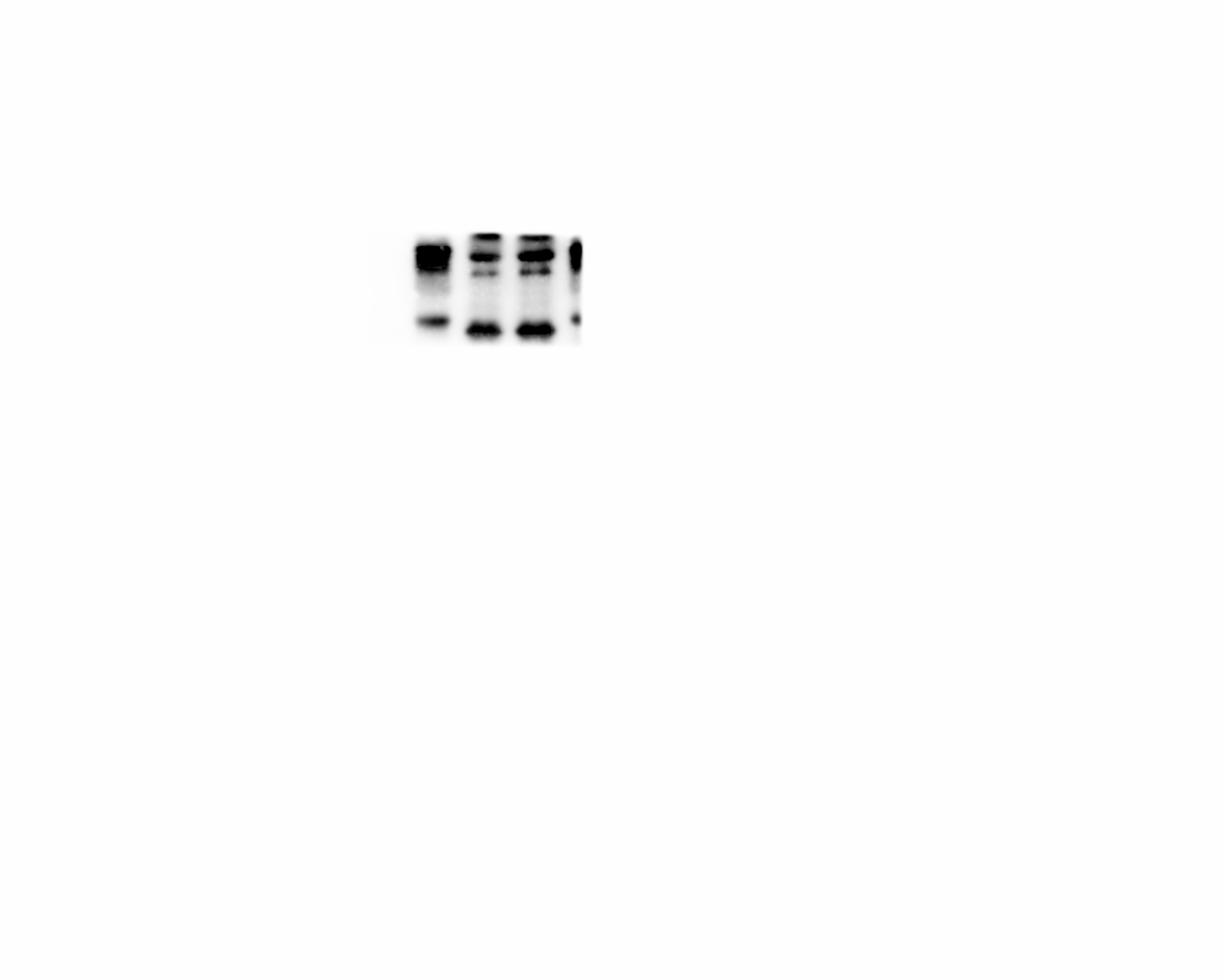

Supplement: Supplemental Information 5 — PZF/PZFX files must be opened using GraphPad Prism. [file peerj-13-19517-s005.zip › FIG _3B-I/FIG 3D/pla2G16-panc-1-250219_1(Chemiluminescence).tif]

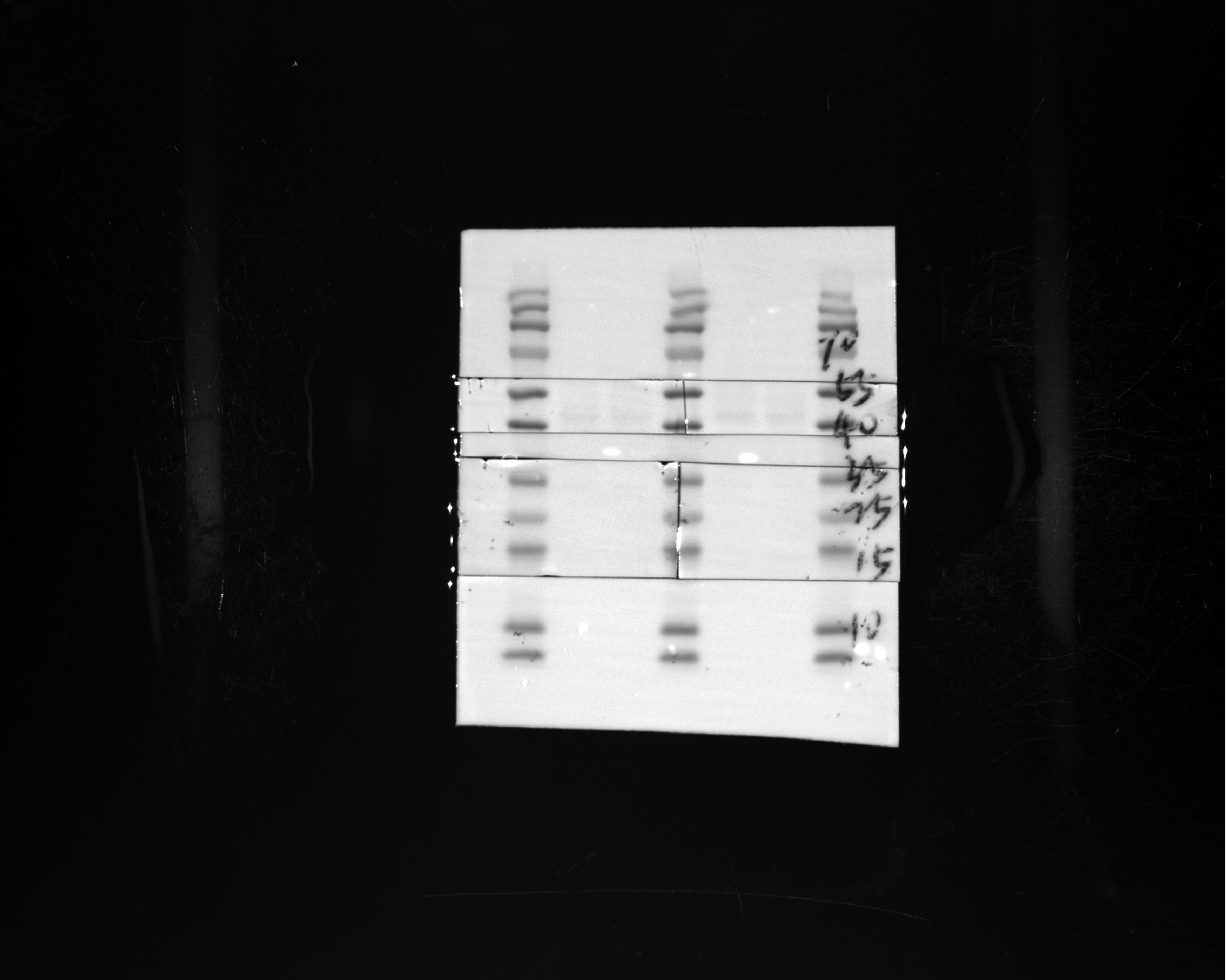

Supplement: Supplemental Information 5 — PZF/PZFX files must be opened using GraphPad Prism. [file peerj-13-19517-s005.zip › FIG _3B-I/FIG 3D/Sui-1 2025-02-19 13h19m58s(Colorimetric).tif]

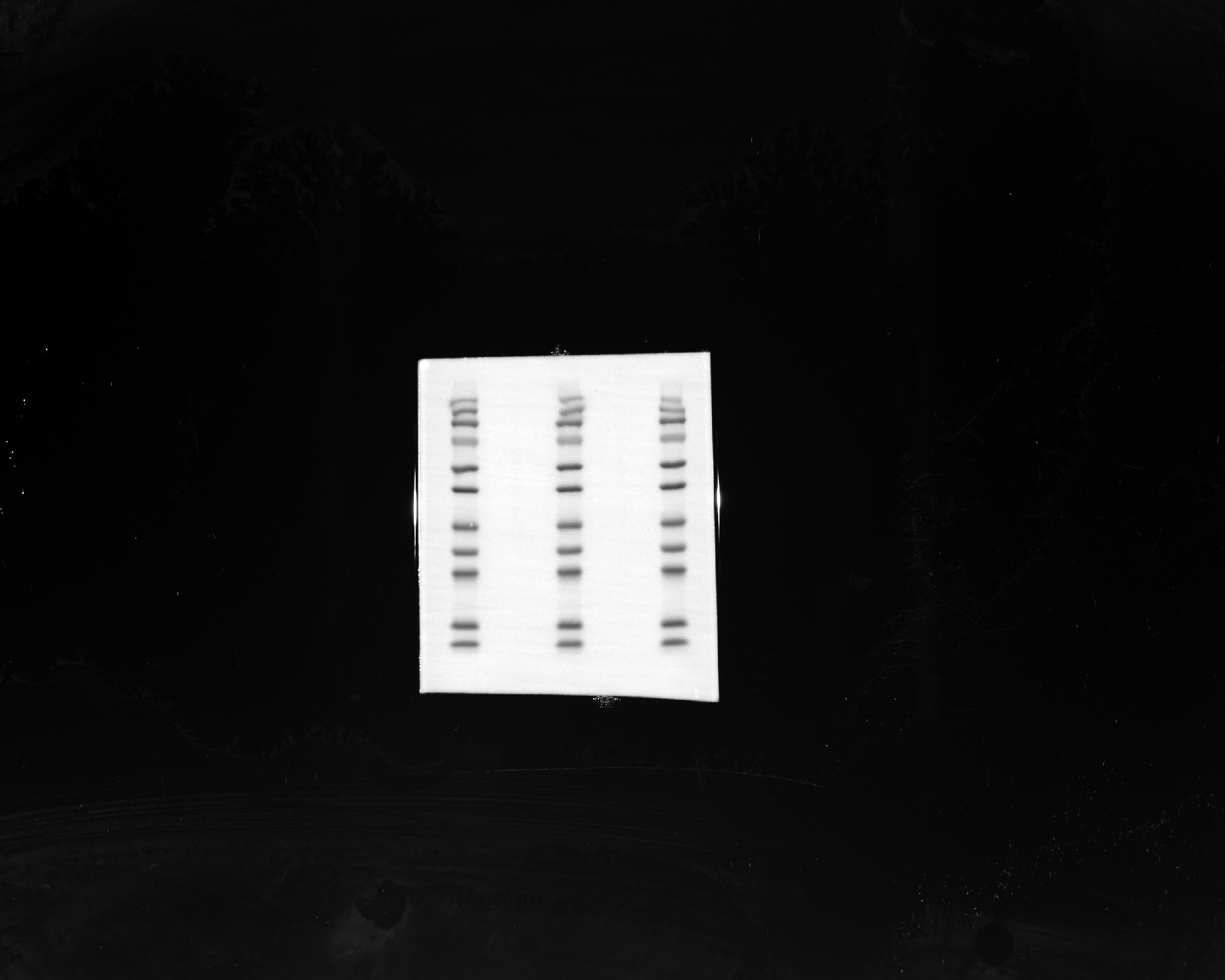

Supplement: Supplemental Information 5 — PZF/PZFX files must be opened using GraphPad Prism. [file peerj-13-19517-s005.zip › FIG _3B-I/FIG 3D/Sui-2 2025-02-17 19h04m39s(Colorimetric).tif]

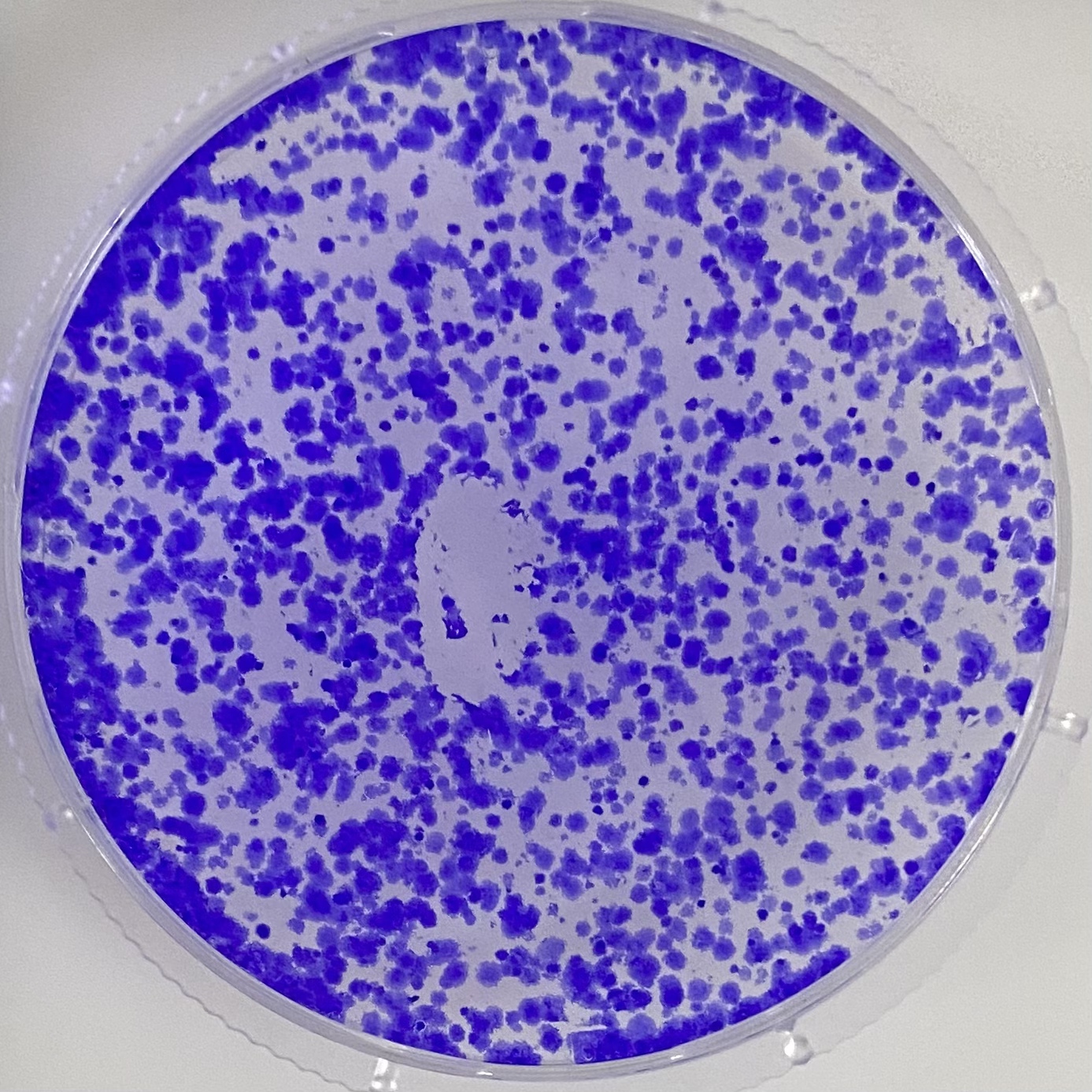

Supplement: Supplemental Information 6 — PZF/PZFX files must be opened using GraphPad Prism. [file peerj-13-19517-s006.zip › FIG 3JK/NC-2.jpg]

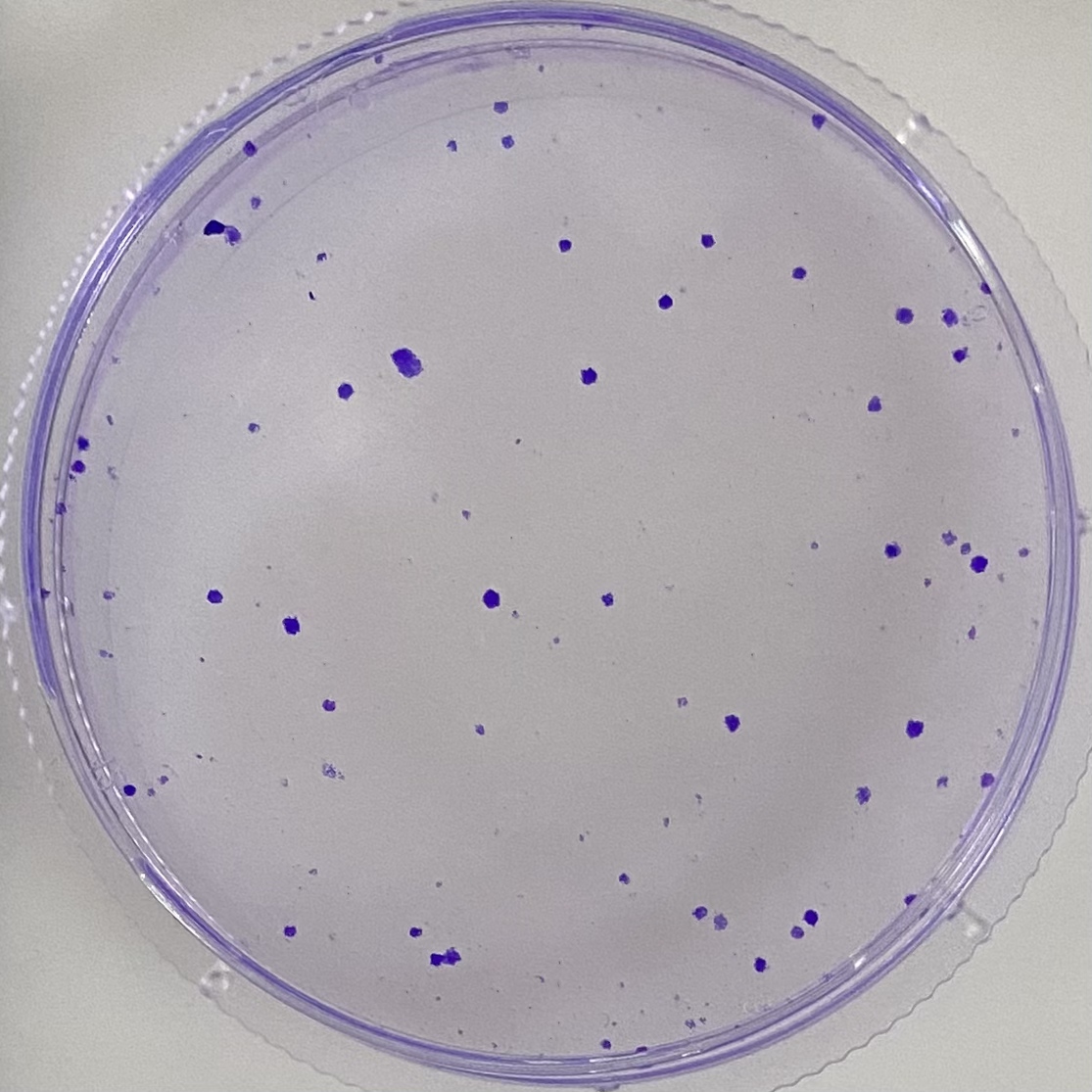

Supplement: Supplemental Information 6 — PZF/PZFX files must be opened using GraphPad Prism. [file peerj-13-19517-s006.zip › FIG 3JK/NC-3.jpg]

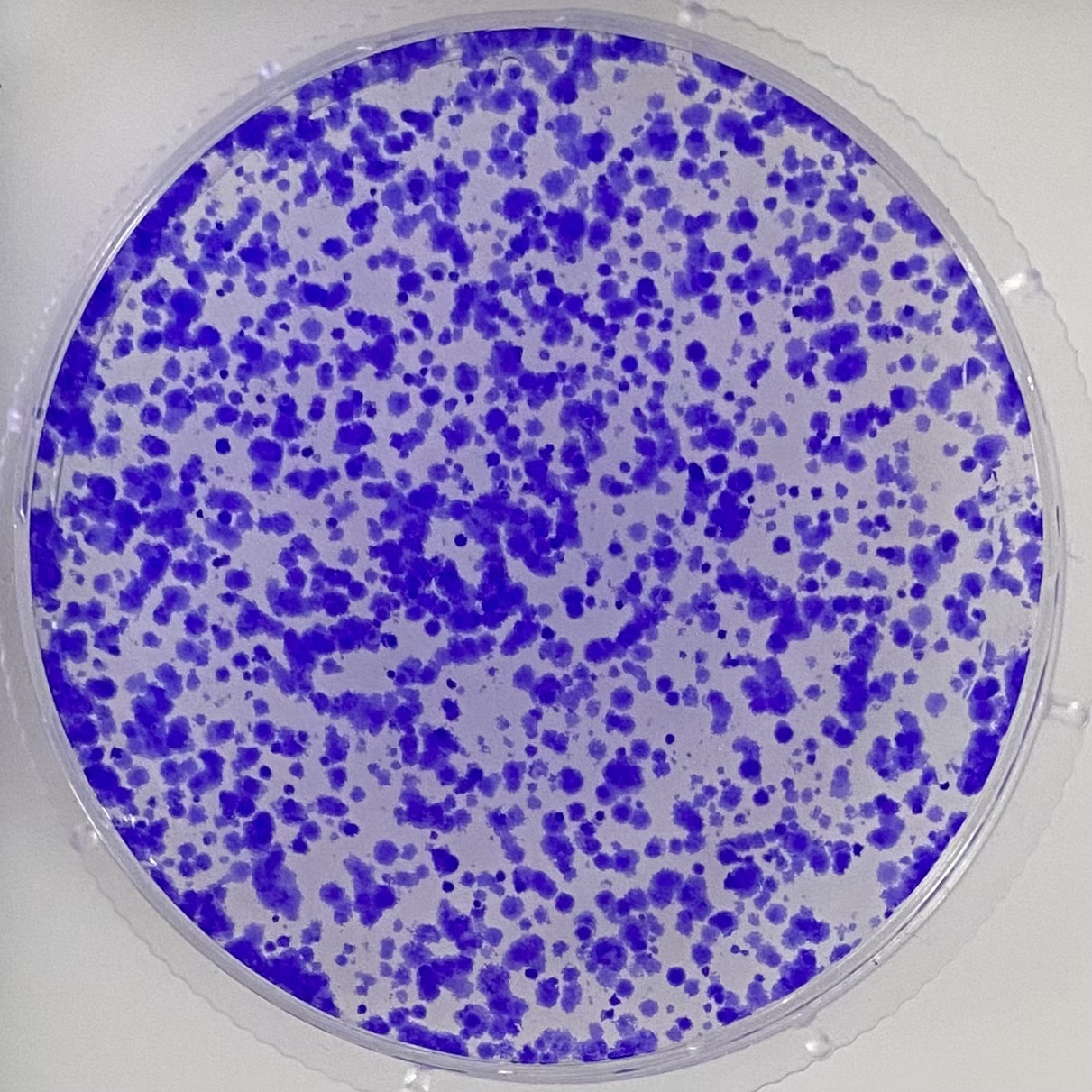

Supplement: Supplemental Information 6 — PZF/PZFX files must be opened using GraphPad Prism. [file peerj-13-19517-s006.zip › FIG 3JK/NC.jpg]

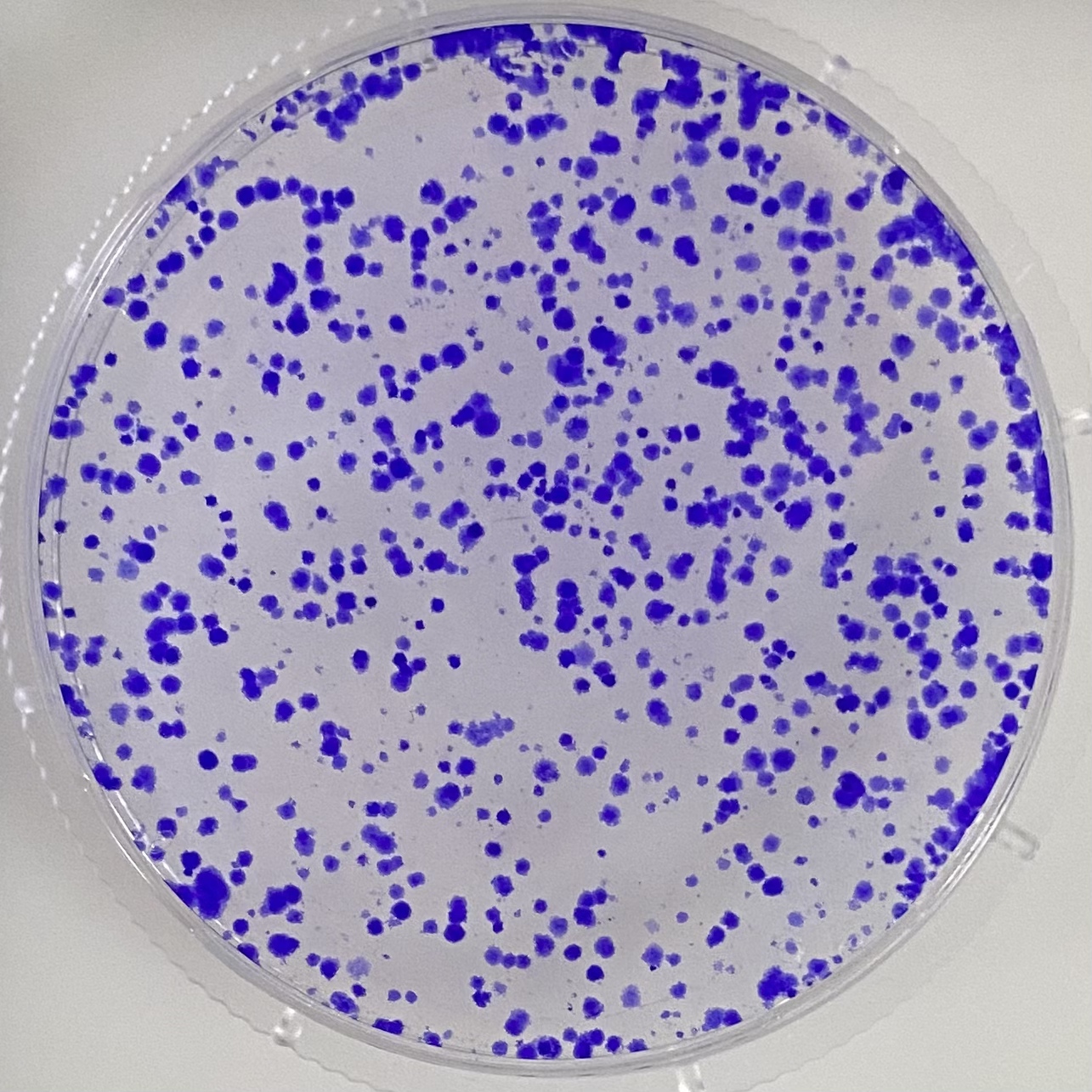

Supplement: Supplemental Information 6 — PZF/PZFX files must be opened using GraphPad Prism. [file peerj-13-19517-s006.zip › FIG 3JK/siPLA2G16-1-2.jpg]

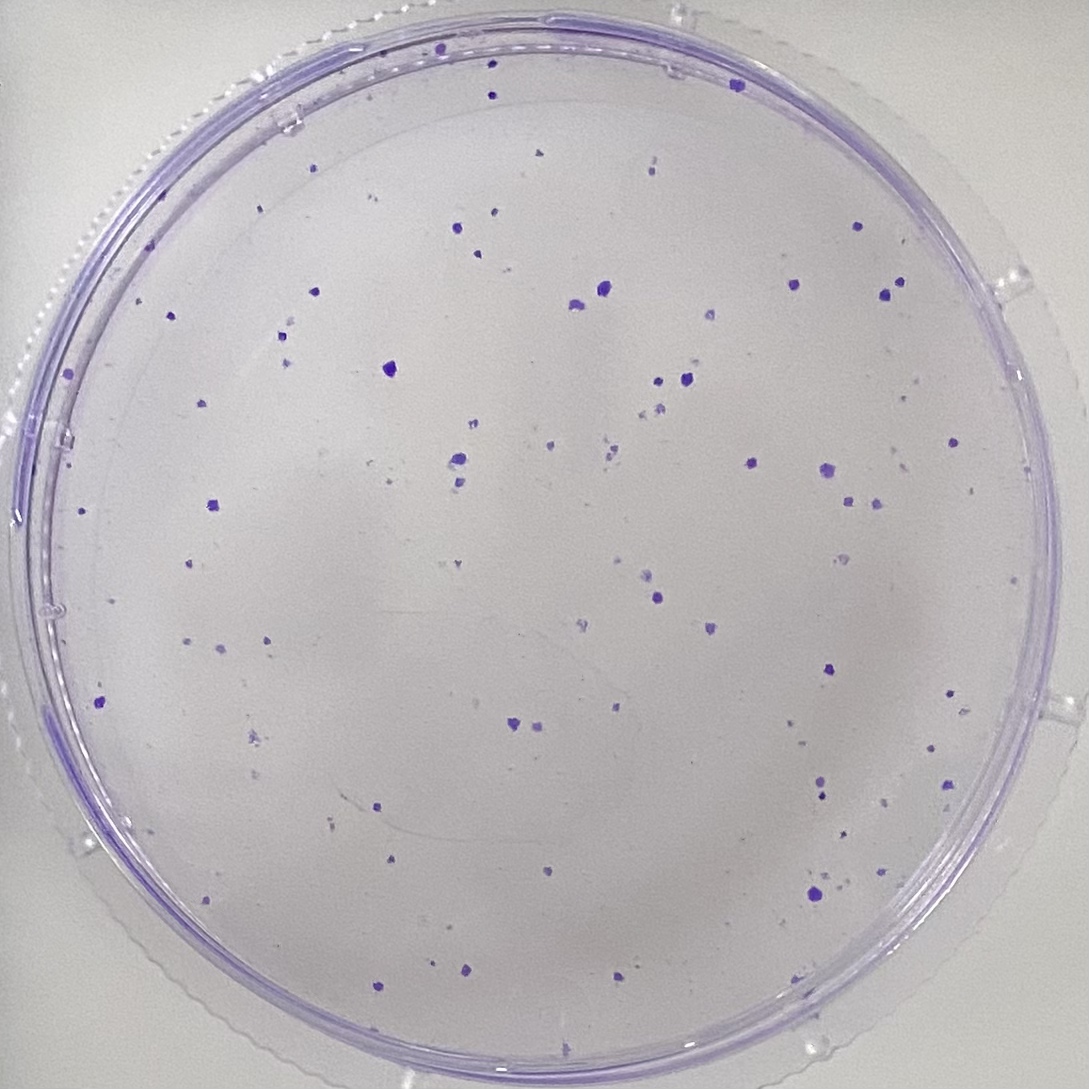

Supplement: Supplemental Information 6 — PZF/PZFX files must be opened using GraphPad Prism. [file peerj-13-19517-s006.zip › FIG 3JK/siPLA2G16-1-3.jpg]

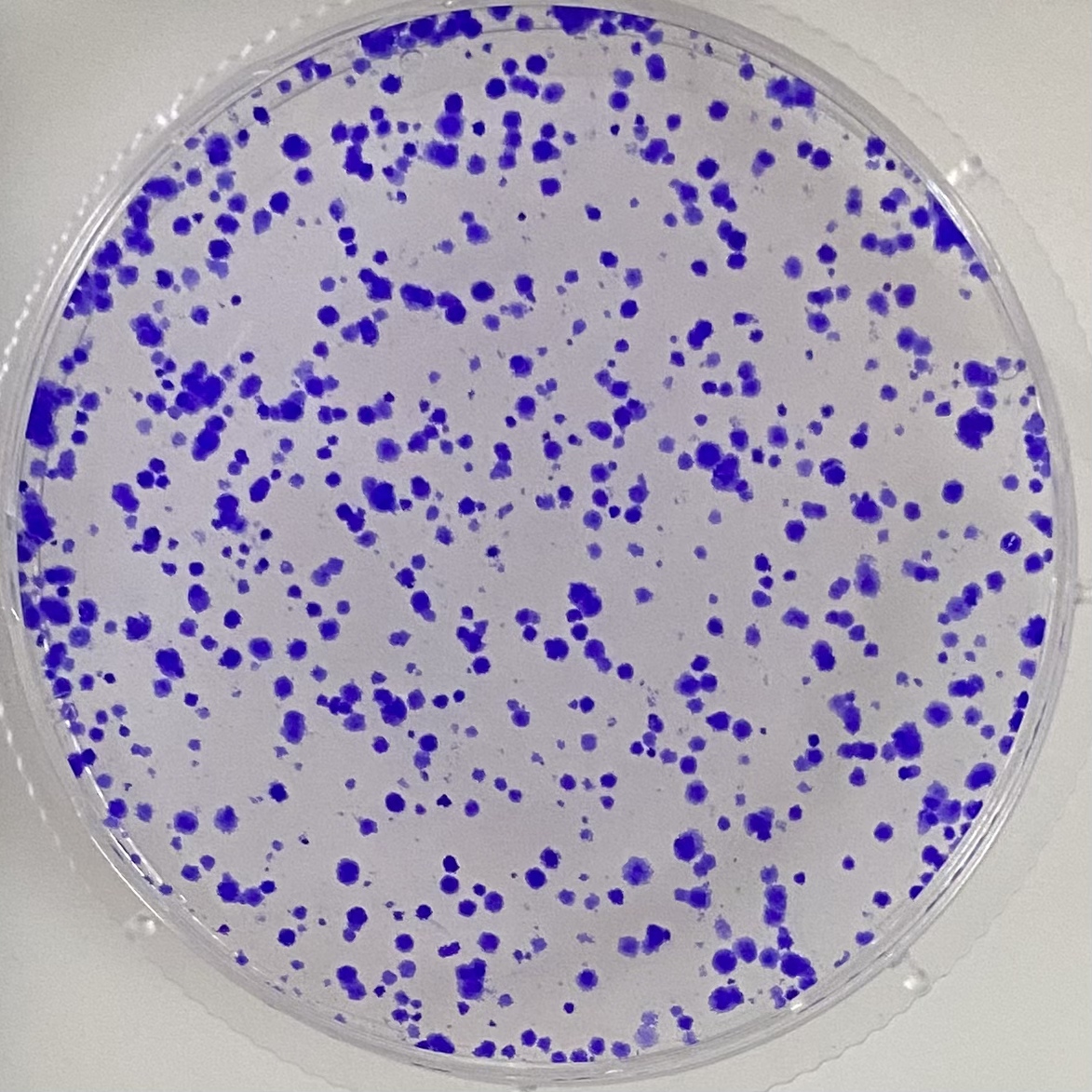

Supplement: Supplemental Information 6 — PZF/PZFX files must be opened using GraphPad Prism. [file peerj-13-19517-s006.zip › FIG 3JK/siPLA2G16-1.jpg]

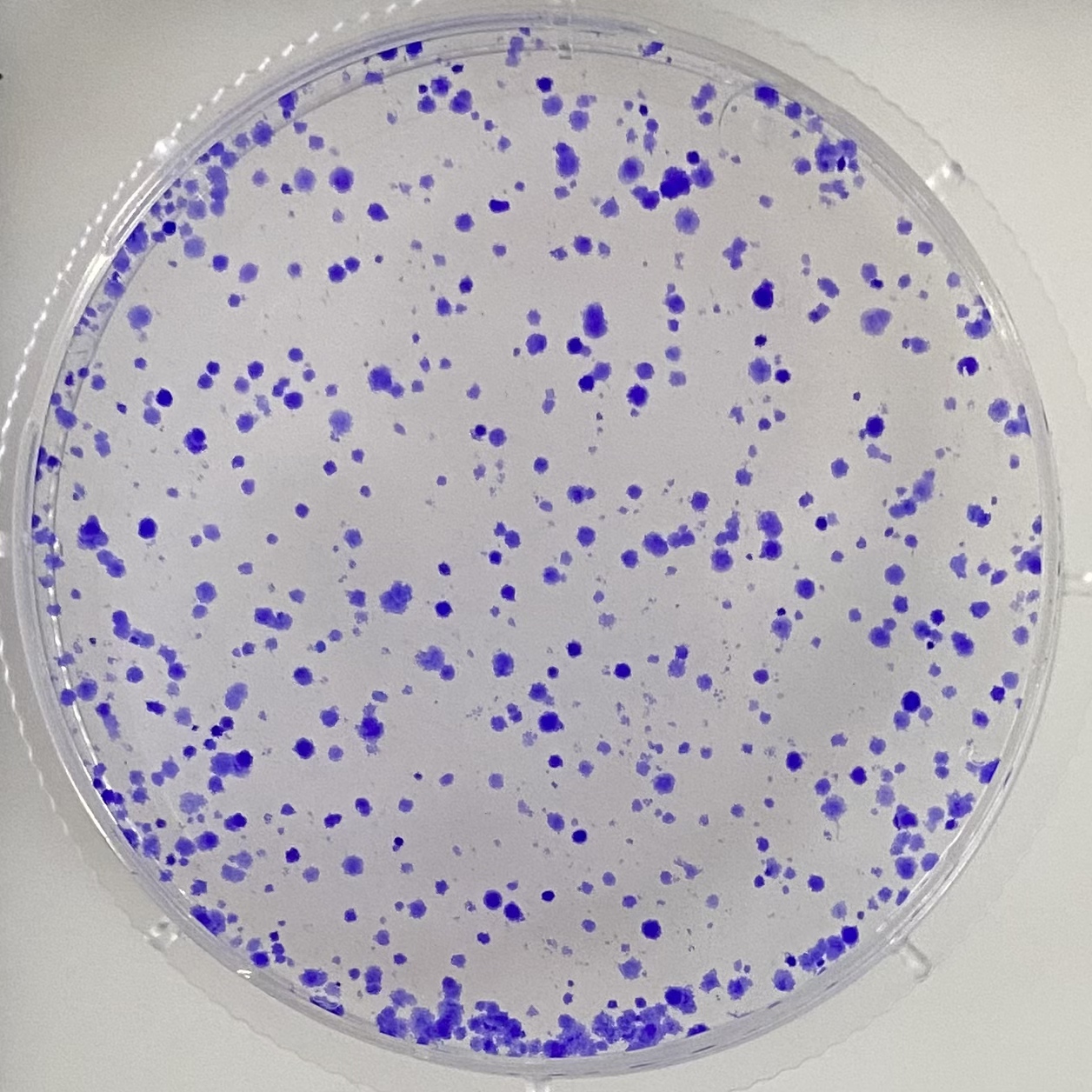

Supplement: Supplemental Information 6 — PZF/PZFX files must be opened using GraphPad Prism. [file peerj-13-19517-s006.zip › FIG 3JK/siPLA2G16-2-2.jpg]

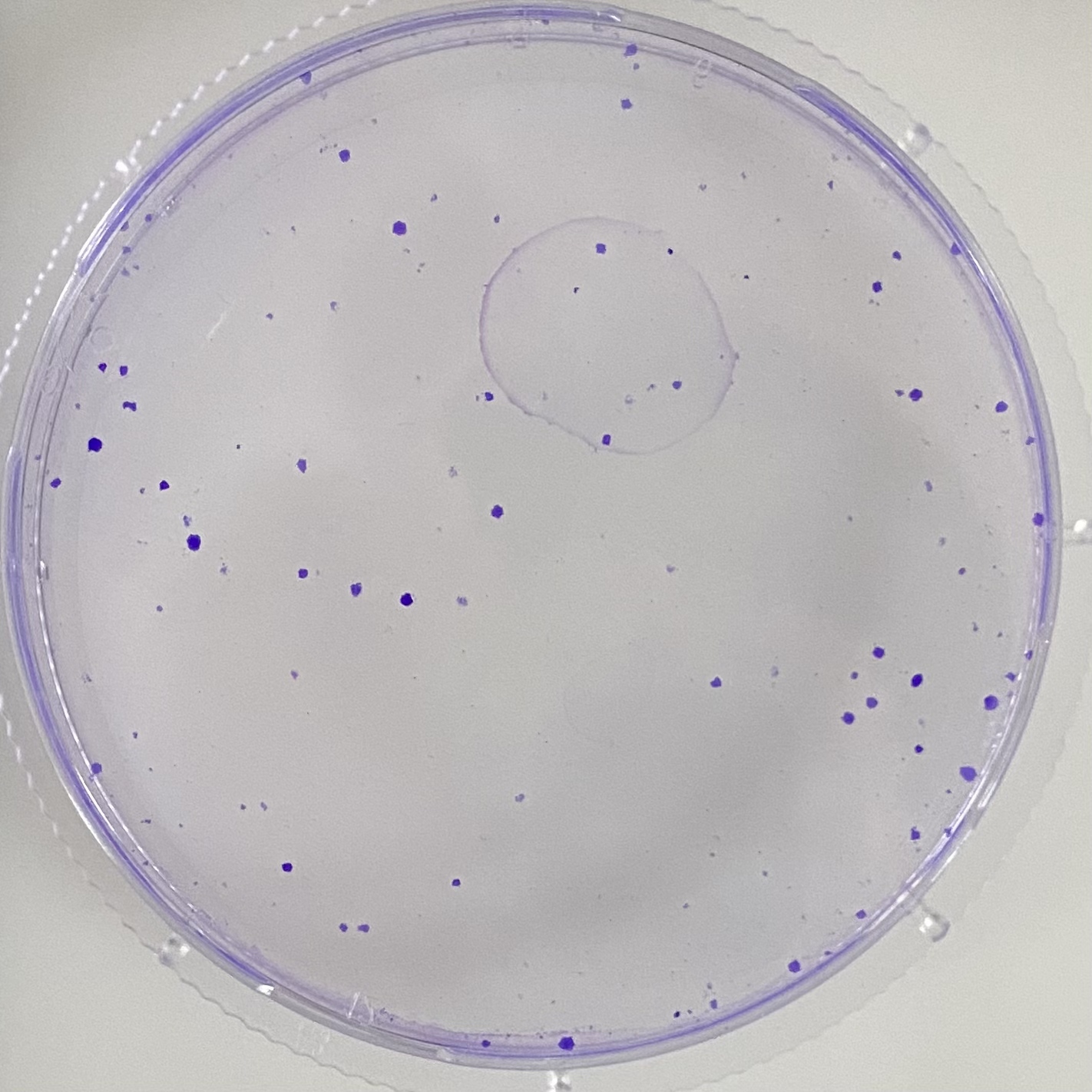

Supplement: Supplemental Information 6 — PZF/PZFX files must be opened using GraphPad Prism. [file peerj-13-19517-s006.zip › FIG 3JK/siPLA2G16-2-3.jpg]

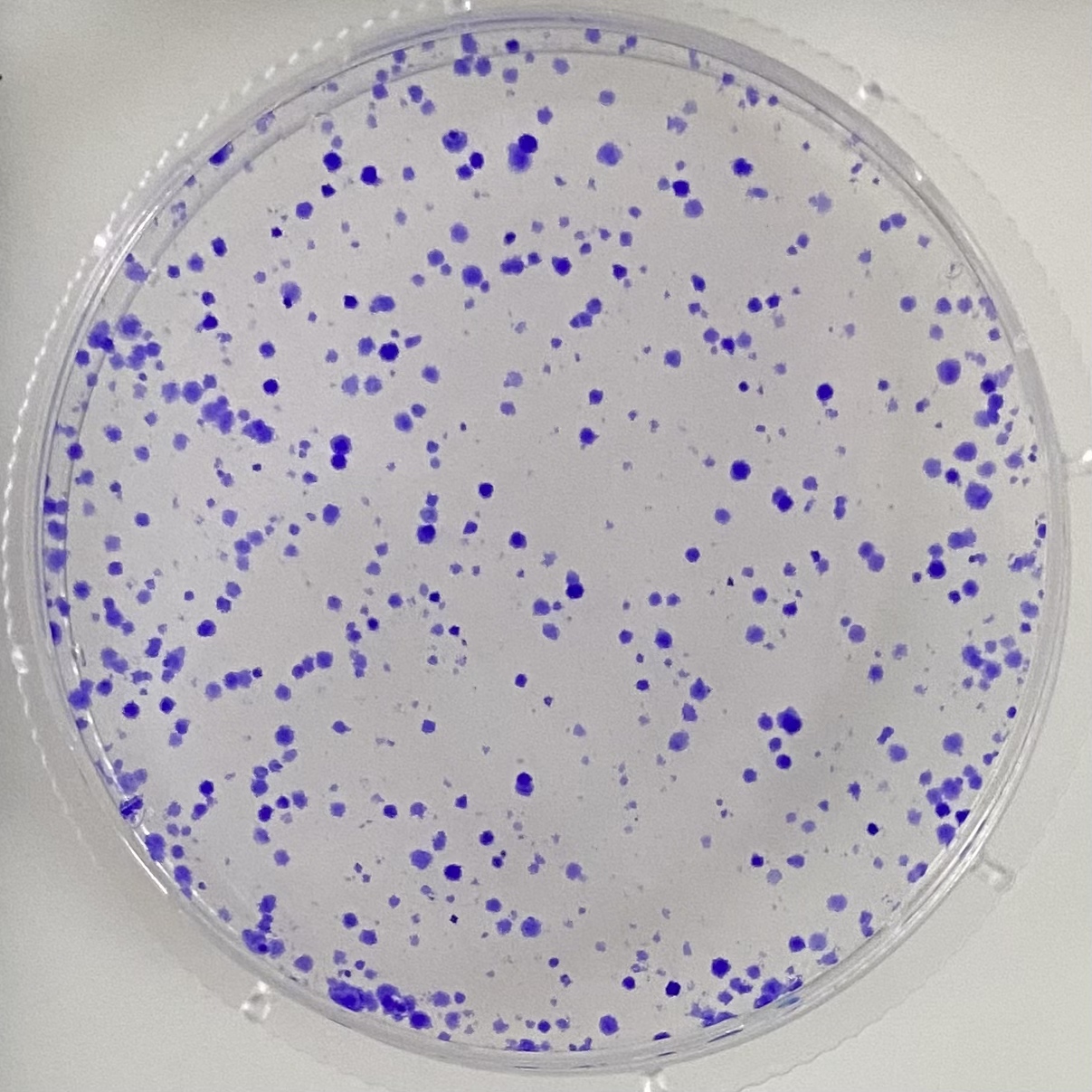

Supplement: Supplemental Information 6 — PZF/PZFX files must be opened using GraphPad Prism. [file peerj-13-19517-s006.zip › FIG 3JK/siPLA2G16-2.jpg]

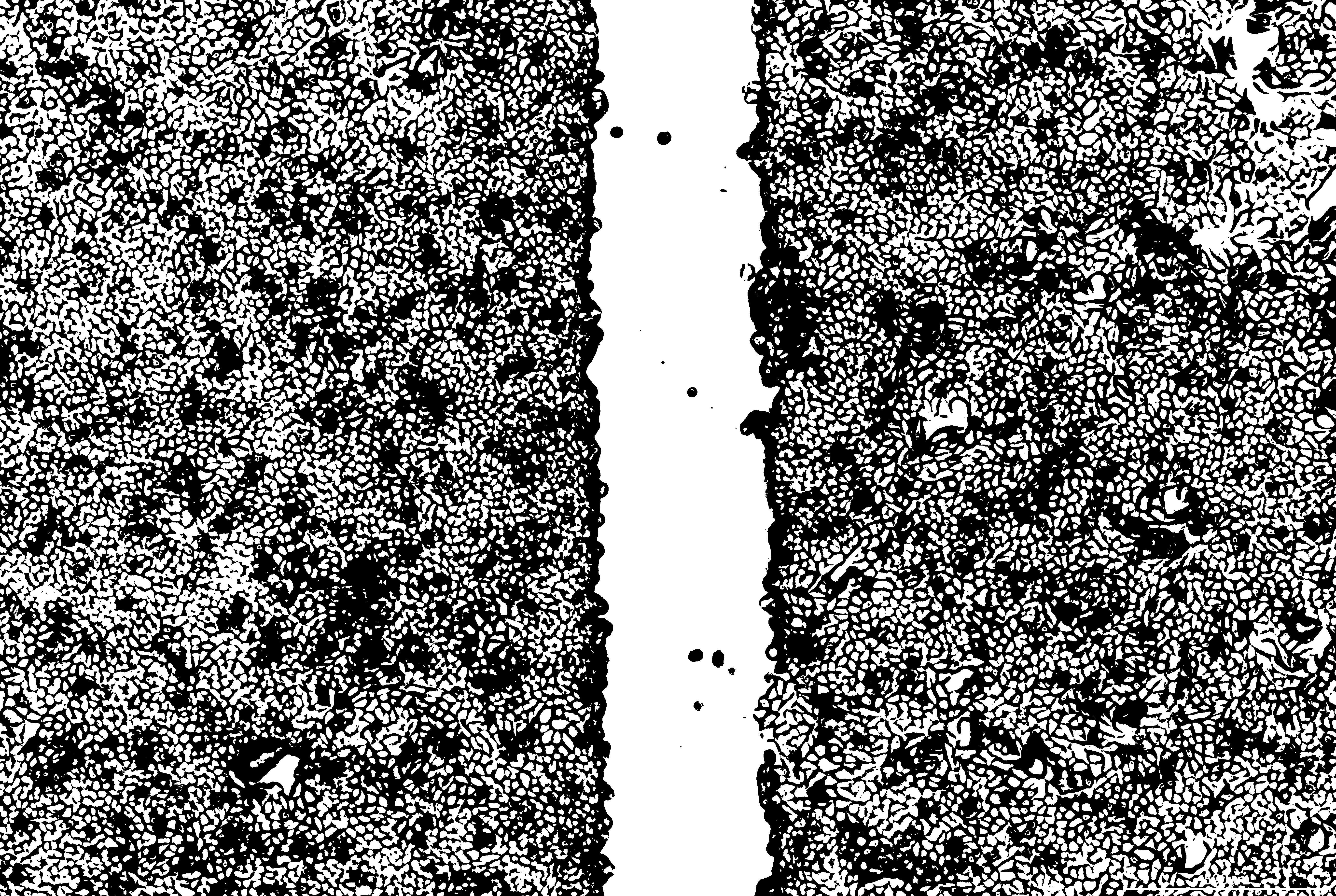

Supplement: Supplemental Information 7 — PZF/PZFX files must be opened using GraphPad Prism. [file peerj-13-19517-s007.zip › FIG 3I/Scratch experiments after imageJ treatment/24.4.12/0001.tif]

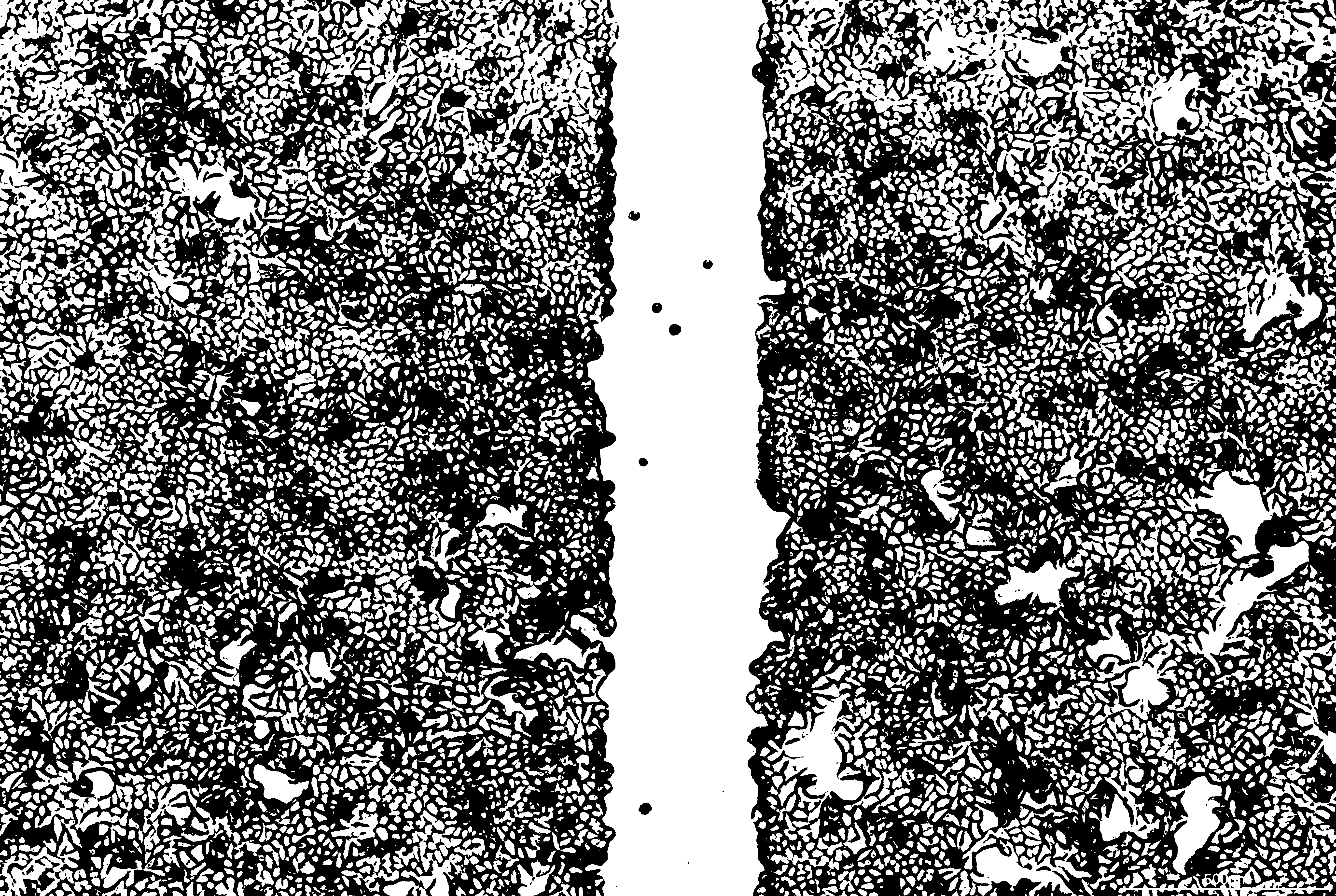

Supplement: Supplemental Information 7 — PZF/PZFX files must be opened using GraphPad Prism. [file peerj-13-19517-s007.zip › FIG 3I/Scratch experiments after imageJ treatment/24.4.12/0002.tif]

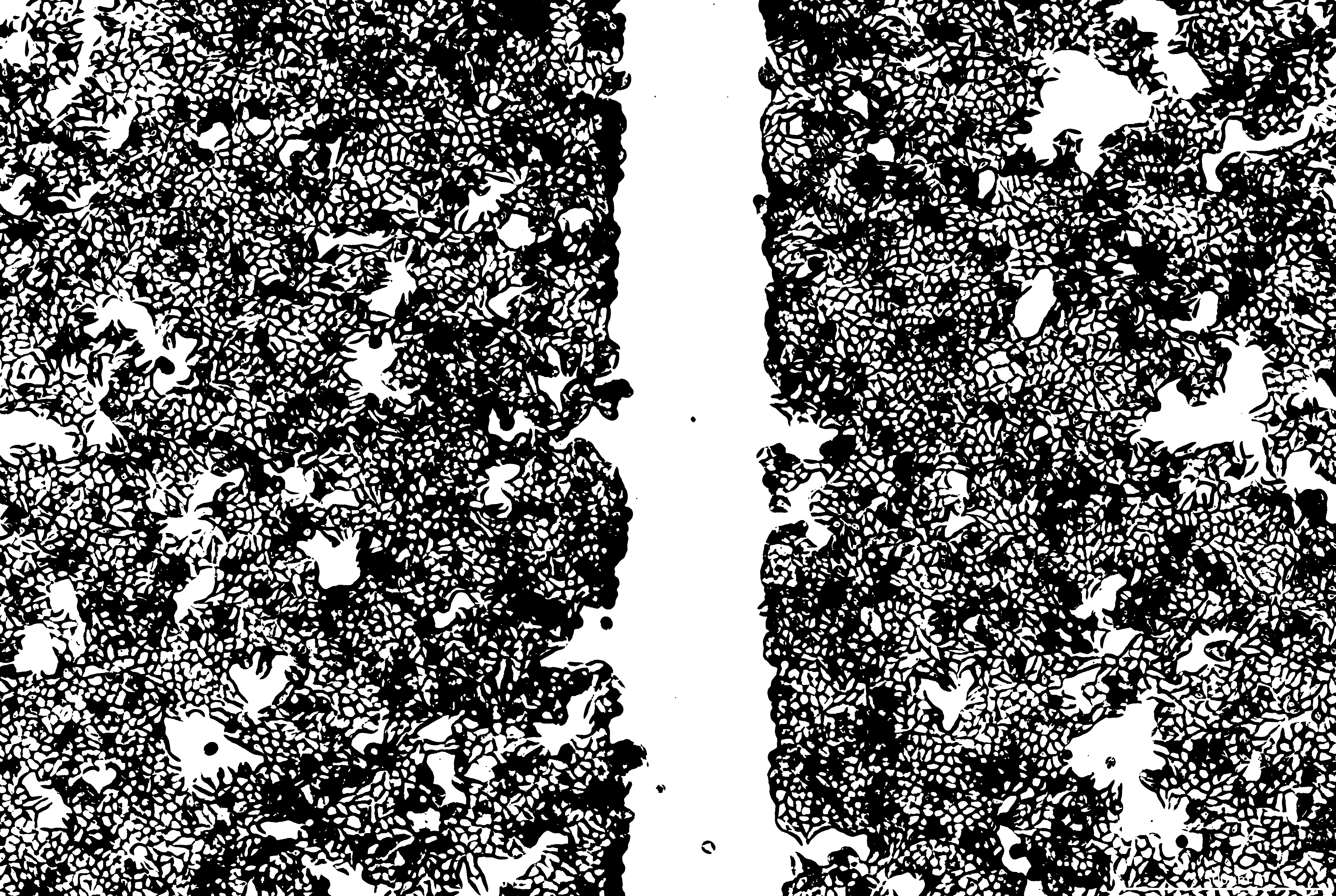

Supplement: Supplemental Information 7 — PZF/PZFX files must be opened using GraphPad Prism. [file peerj-13-19517-s007.zip › FIG 3I/Scratch experiments after imageJ treatment/24.4.12/0003.tif]

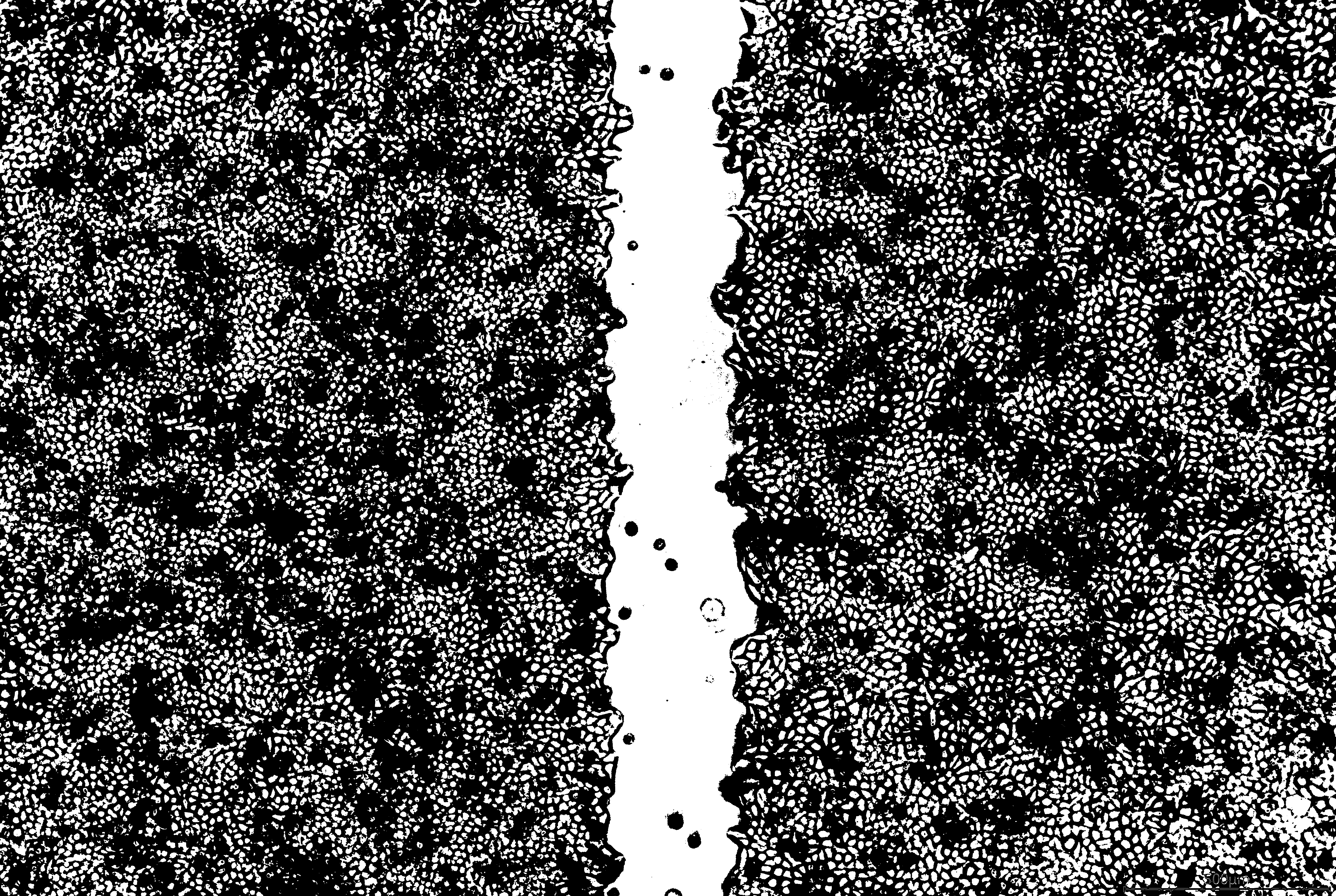

Supplement: Supplemental Information 7 — PZF/PZFX files must be opened using GraphPad Prism. [file peerj-13-19517-s007.zip › FIG 3I/Scratch experiments after imageJ treatment/24.4.12/24H/0001.tif]

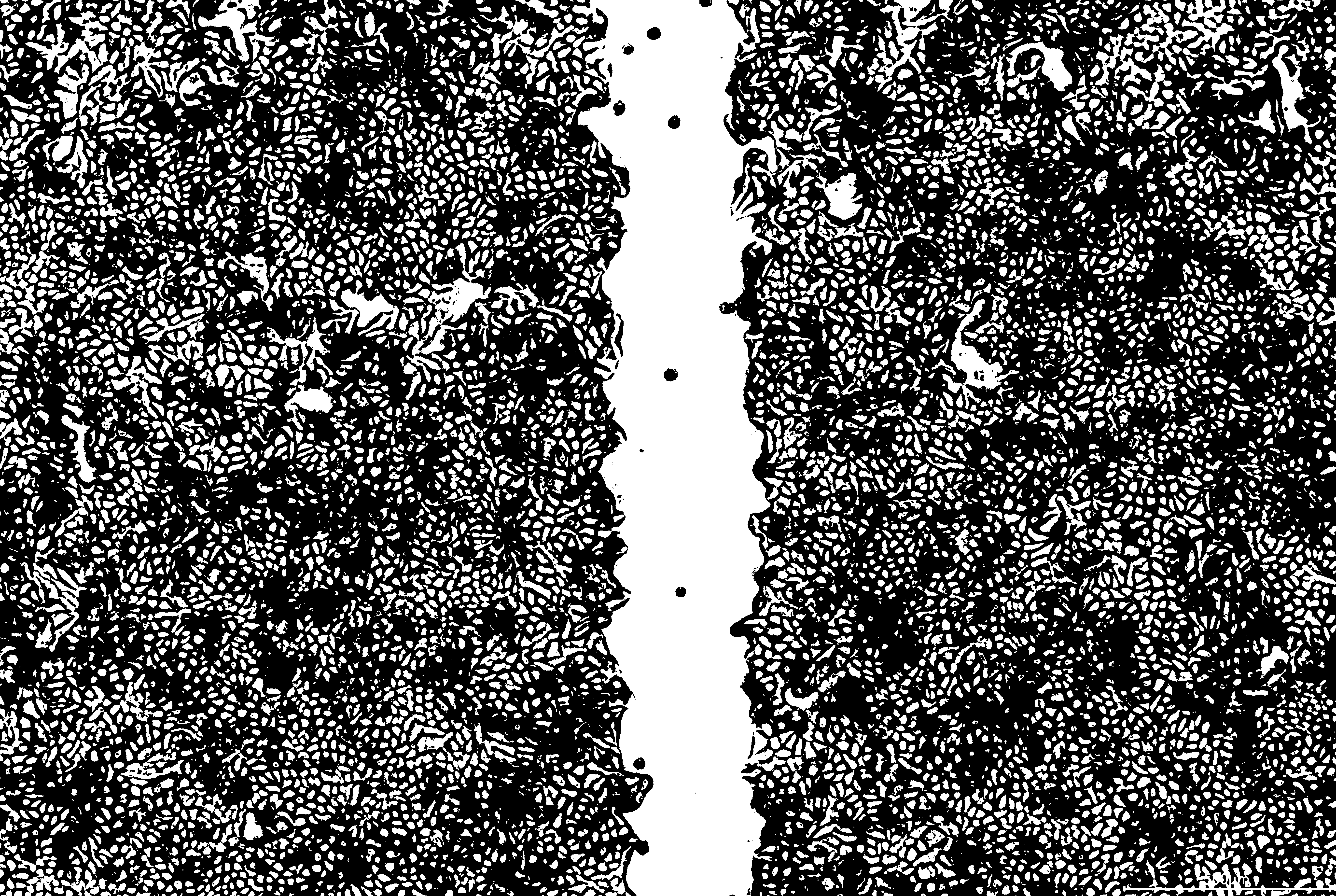

Supplement: Supplemental Information 7 — PZF/PZFX files must be opened using GraphPad Prism. [file peerj-13-19517-s007.zip › FIG 3I/Scratch experiments after imageJ treatment/24.4.12/24H/0002.tif]

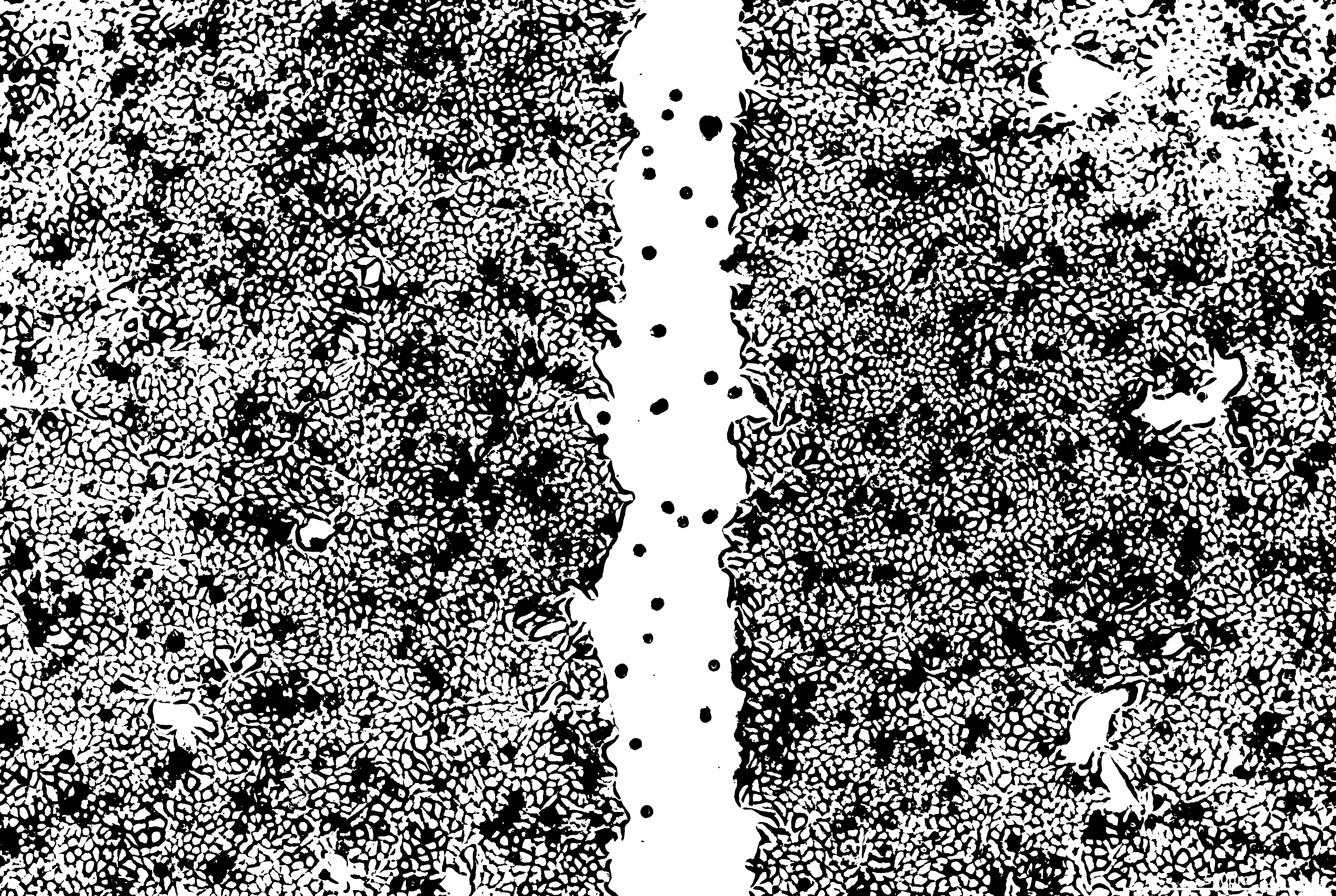

Supplement: Supplemental Information 7 — PZF/PZFX files must be opened using GraphPad Prism. [file peerj-13-19517-s007.zip › FIG 3I/Scratch experiments after imageJ treatment/24.4.12/24H/0003.tif]

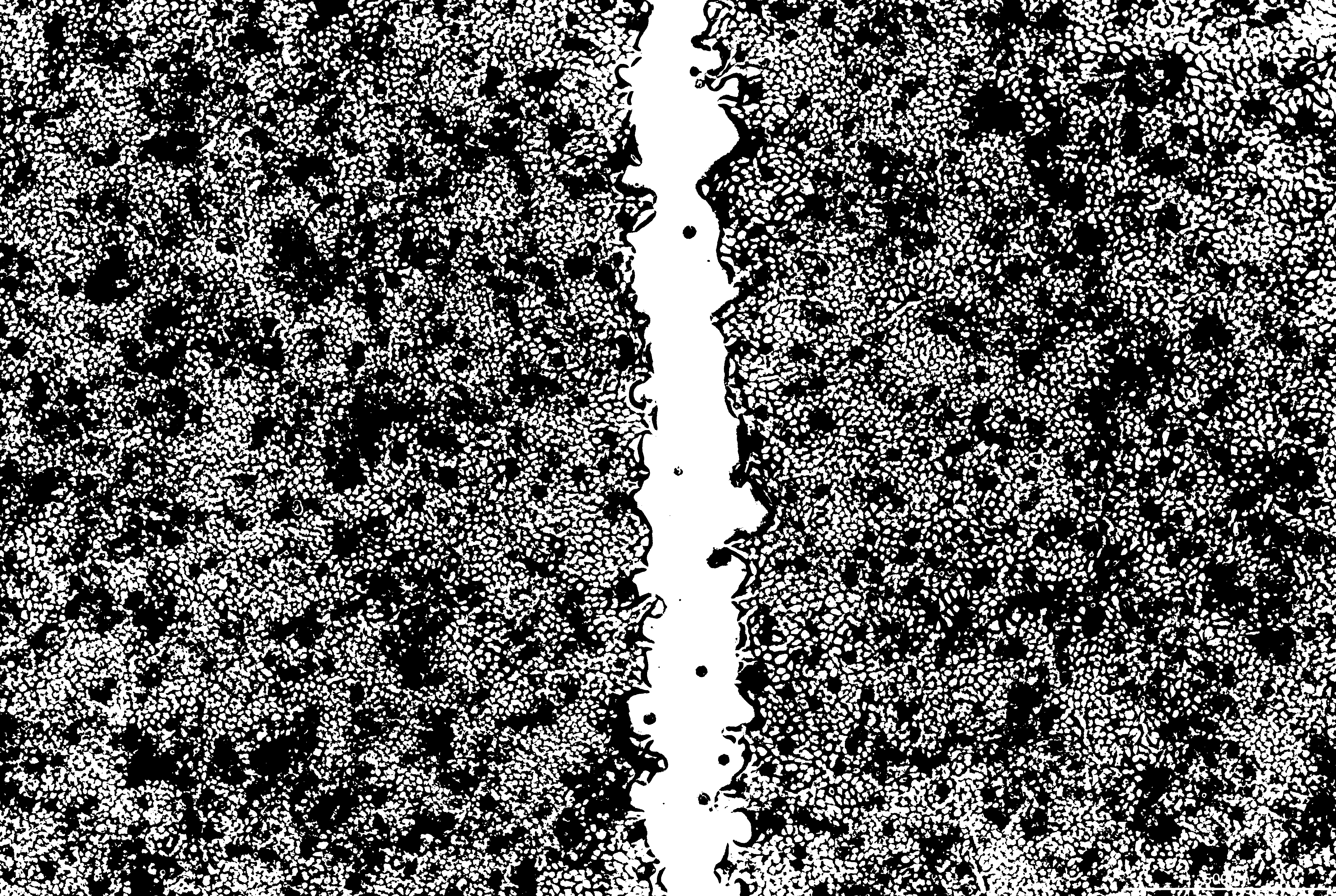

Supplement: Supplemental Information 7 — PZF/PZFX files must be opened using GraphPad Prism. [file peerj-13-19517-s007.zip › FIG 3I/Scratch experiments after imageJ treatment/24.4.12/24H/新建文件夹/0001.tif]

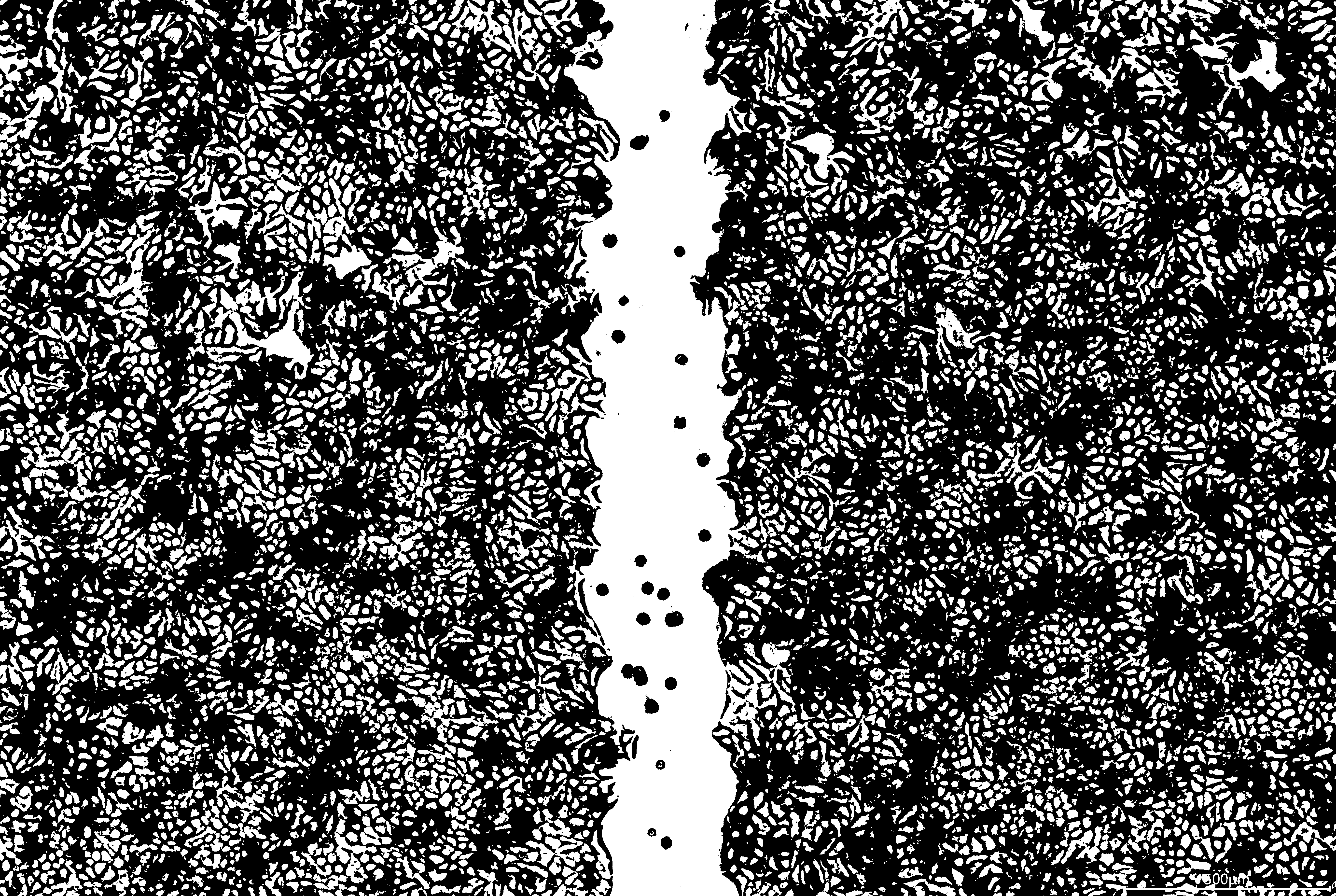

Supplement: Supplemental Information 7 — PZF/PZFX files must be opened using GraphPad Prism. [file peerj-13-19517-s007.zip › FIG 3I/Scratch experiments after imageJ treatment/24.4.12/24H/新建文件夹/0002.tif]

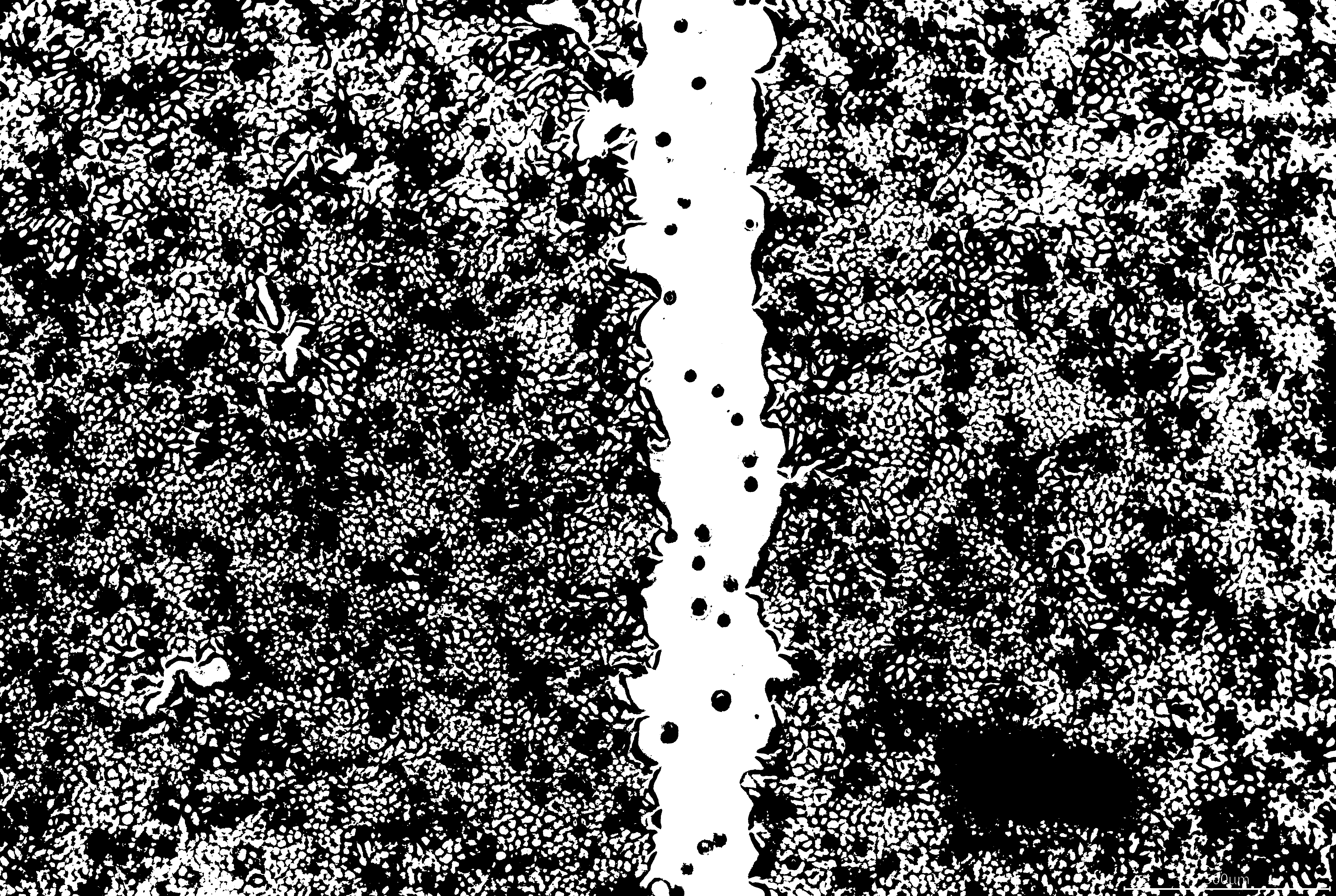

Supplement: Supplemental Information 7 — PZF/PZFX files must be opened using GraphPad Prism. [file peerj-13-19517-s007.zip › FIG 3I/Scratch experiments after imageJ treatment/24.4.12/24H/新建文件夹/0003.tif]

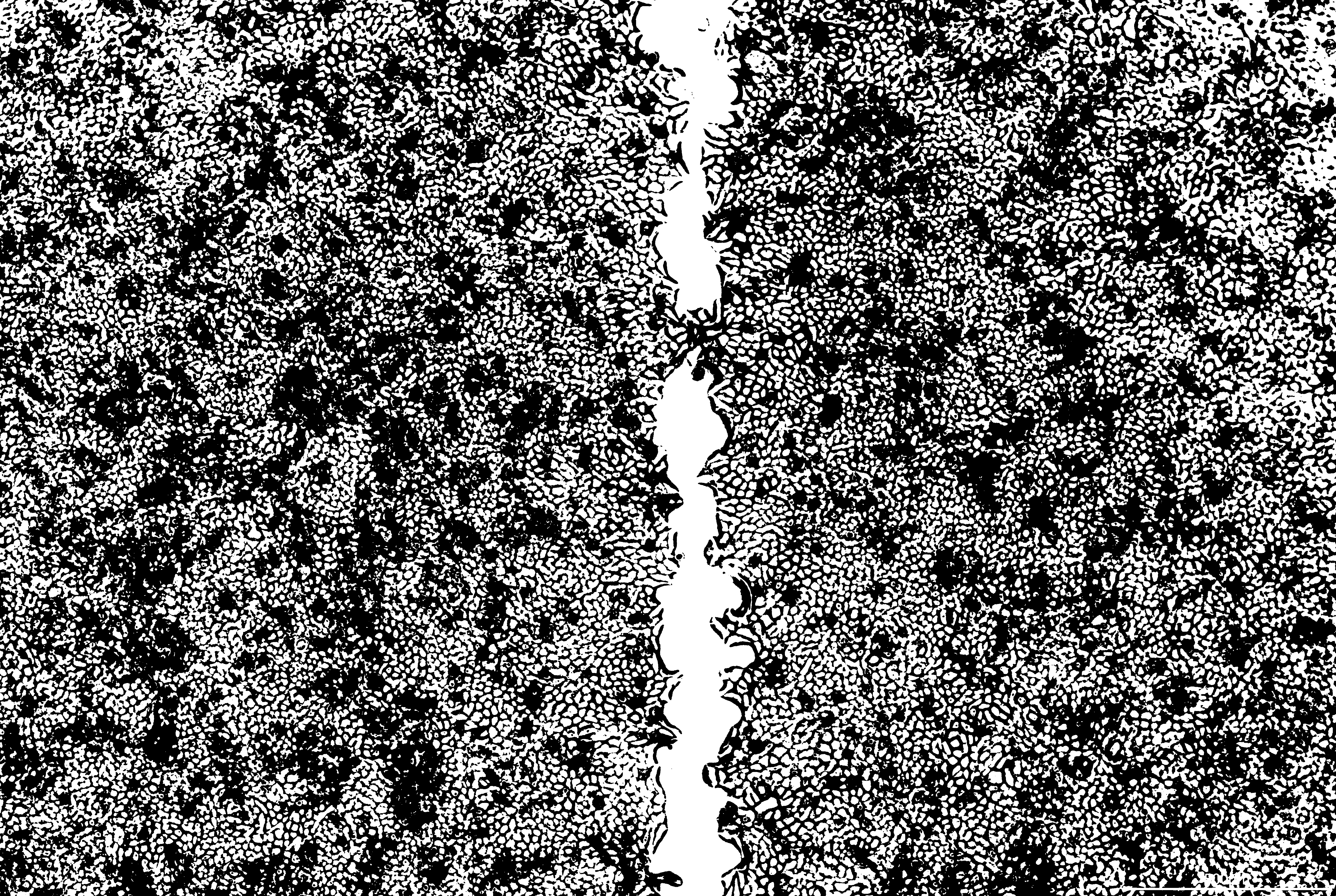

Supplement: Supplemental Information 7 — PZF/PZFX files must be opened using GraphPad Prism. [file peerj-13-19517-s007.zip › FIG 3I/Scratch experiments after imageJ treatment/24.4.12/24H/新建文件夹/新建文件夹/0001.tif]

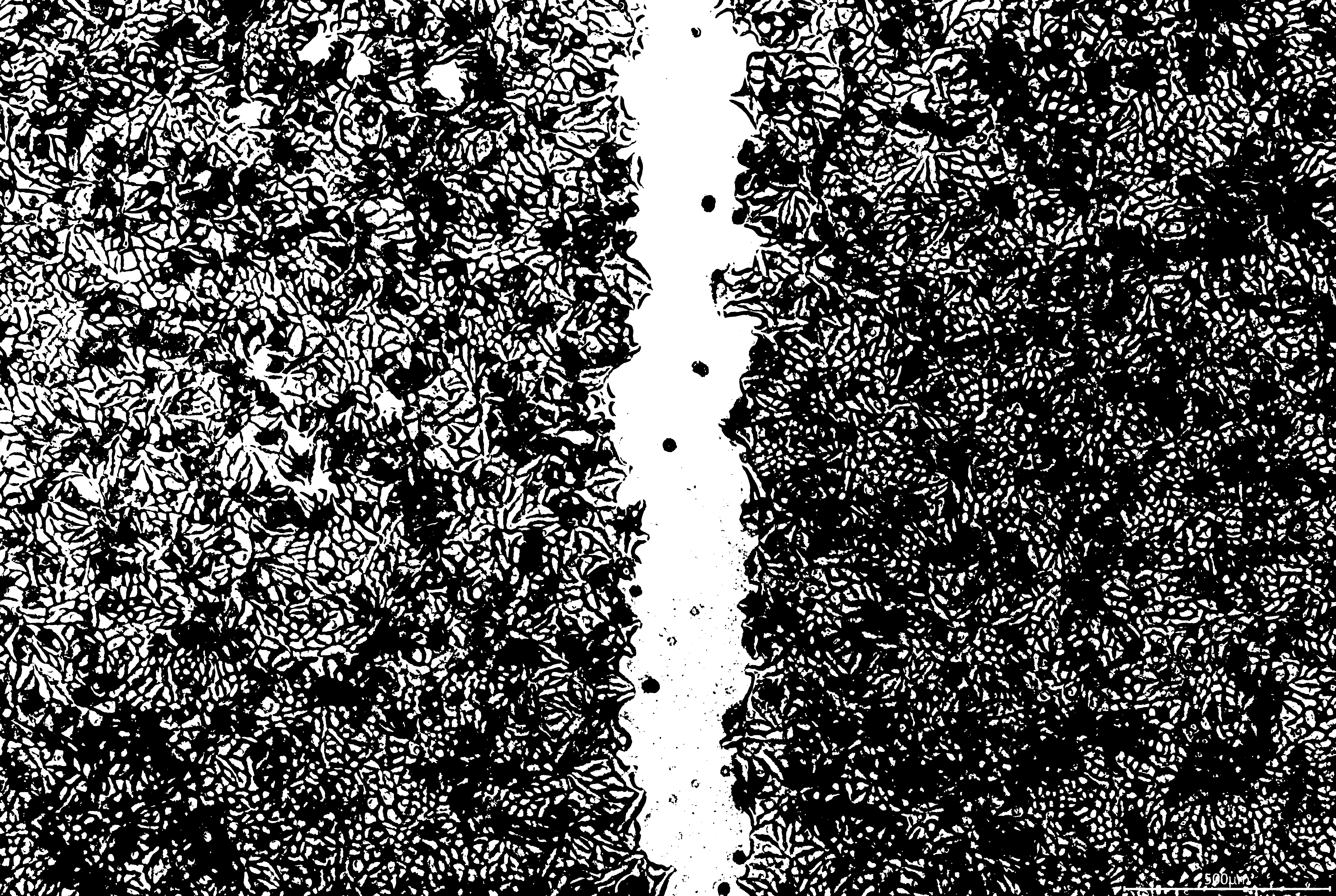

Supplement: Supplemental Information 7 — PZF/PZFX files must be opened using GraphPad Prism. [file peerj-13-19517-s007.zip › FIG 3I/Scratch experiments after imageJ treatment/24.4.12/24H/新建文件夹/新建文件夹/0002.tif]

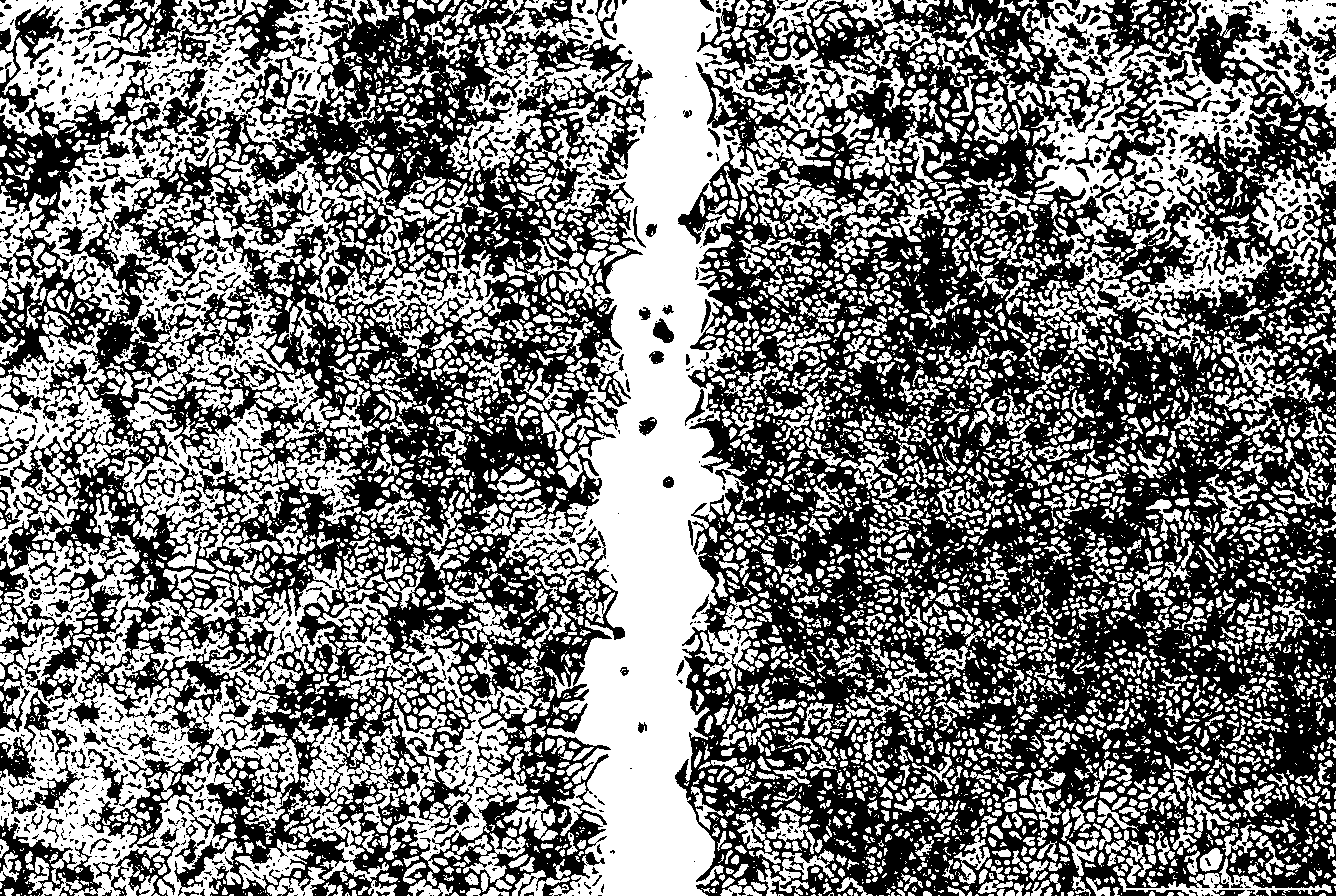

Supplement: Supplemental Information 7 — PZF/PZFX files must be opened using GraphPad Prism. [file peerj-13-19517-s007.zip › FIG 3I/Scratch experiments after imageJ treatment/24.4.12/24H/新建文件夹/新建文件夹/0003.tif]

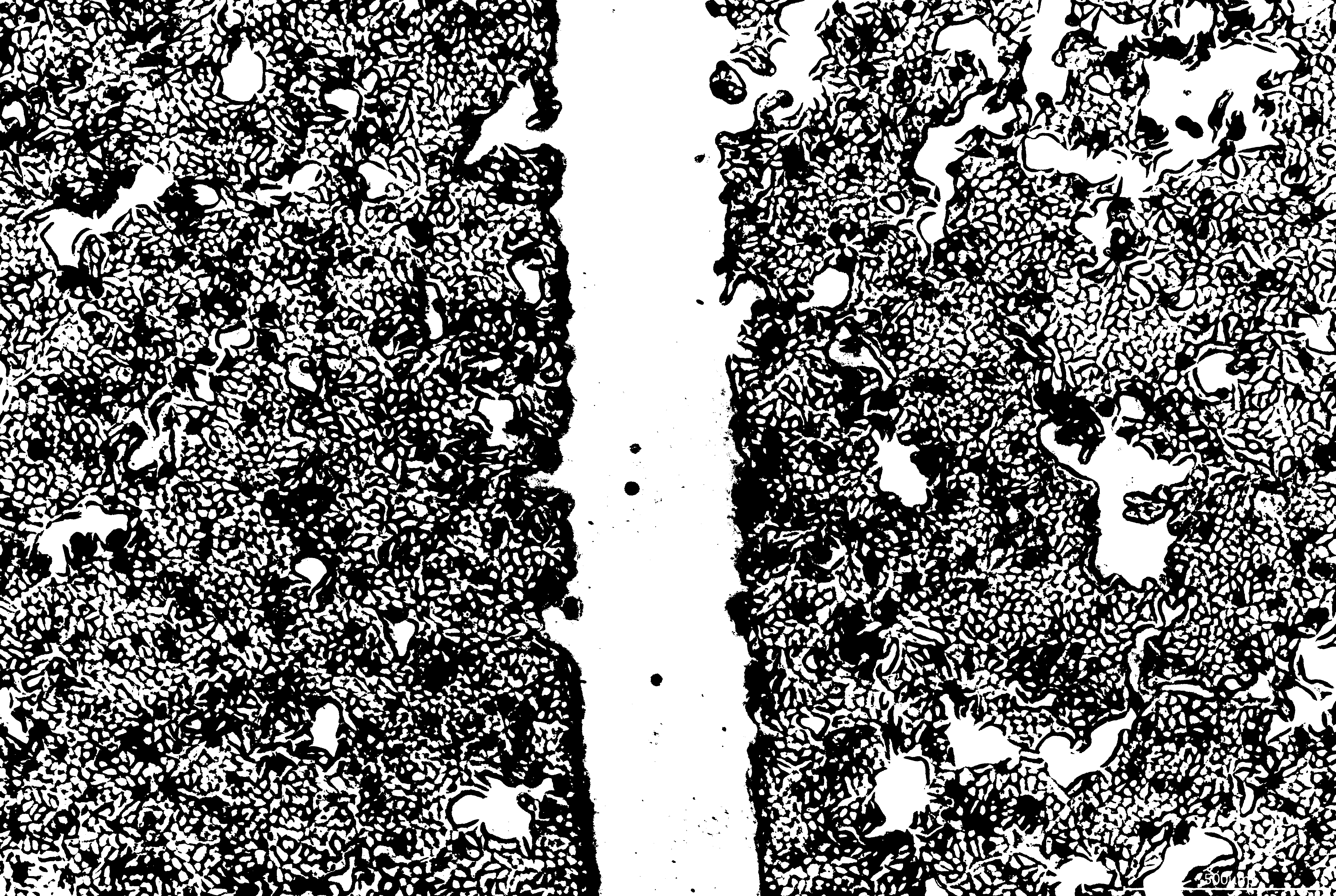

Supplement: Supplemental Information 7 — PZF/PZFX files must be opened using GraphPad Prism. [file peerj-13-19517-s007.zip › FIG 3I/Scratch experiments after imageJ treatment/24.4.8/0001.tif]

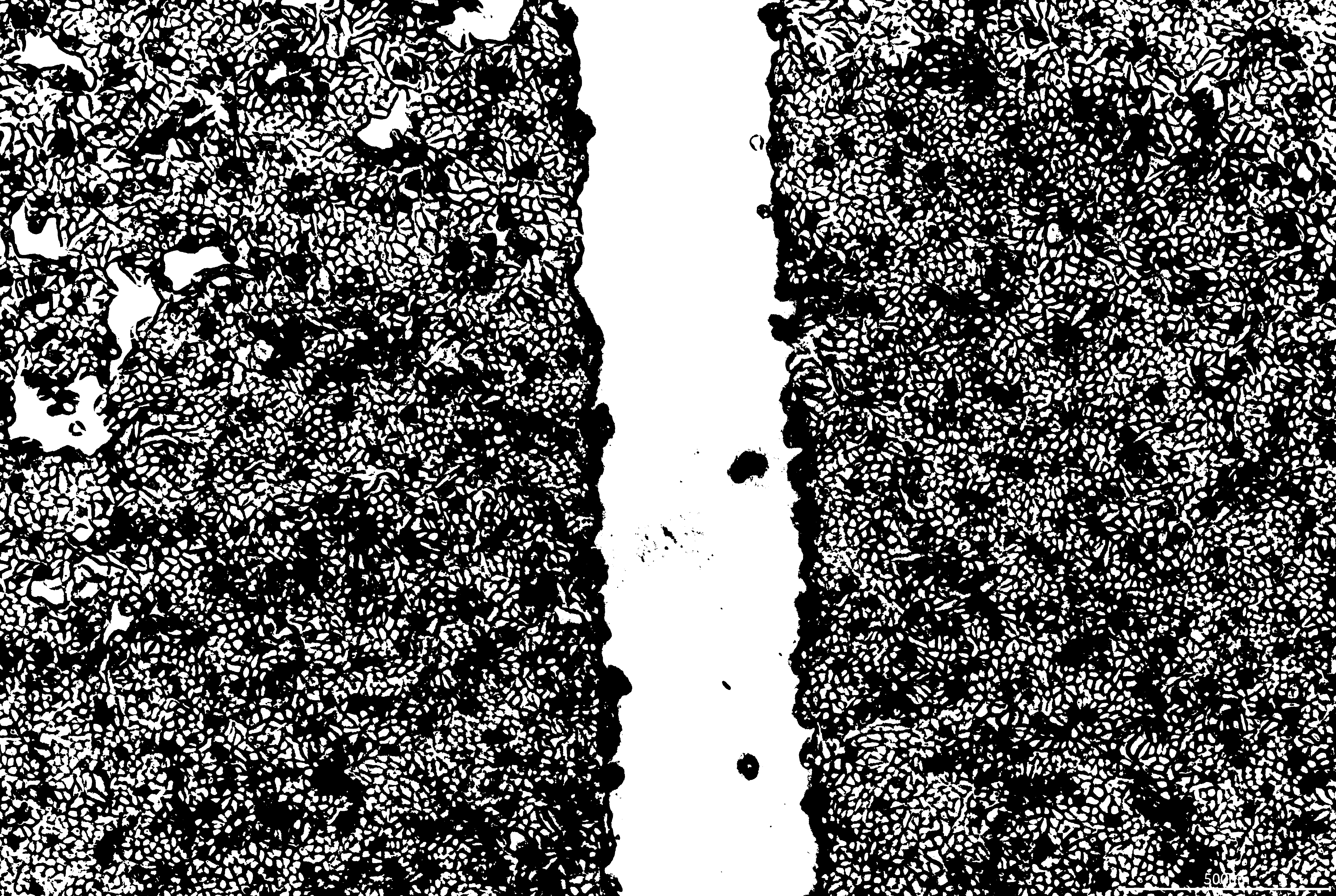

Supplement: Supplemental Information 7 — PZF/PZFX files must be opened using GraphPad Prism. [file peerj-13-19517-s007.zip › FIG 3I/Scratch experiments after imageJ treatment/24.4.8/0002.tif]

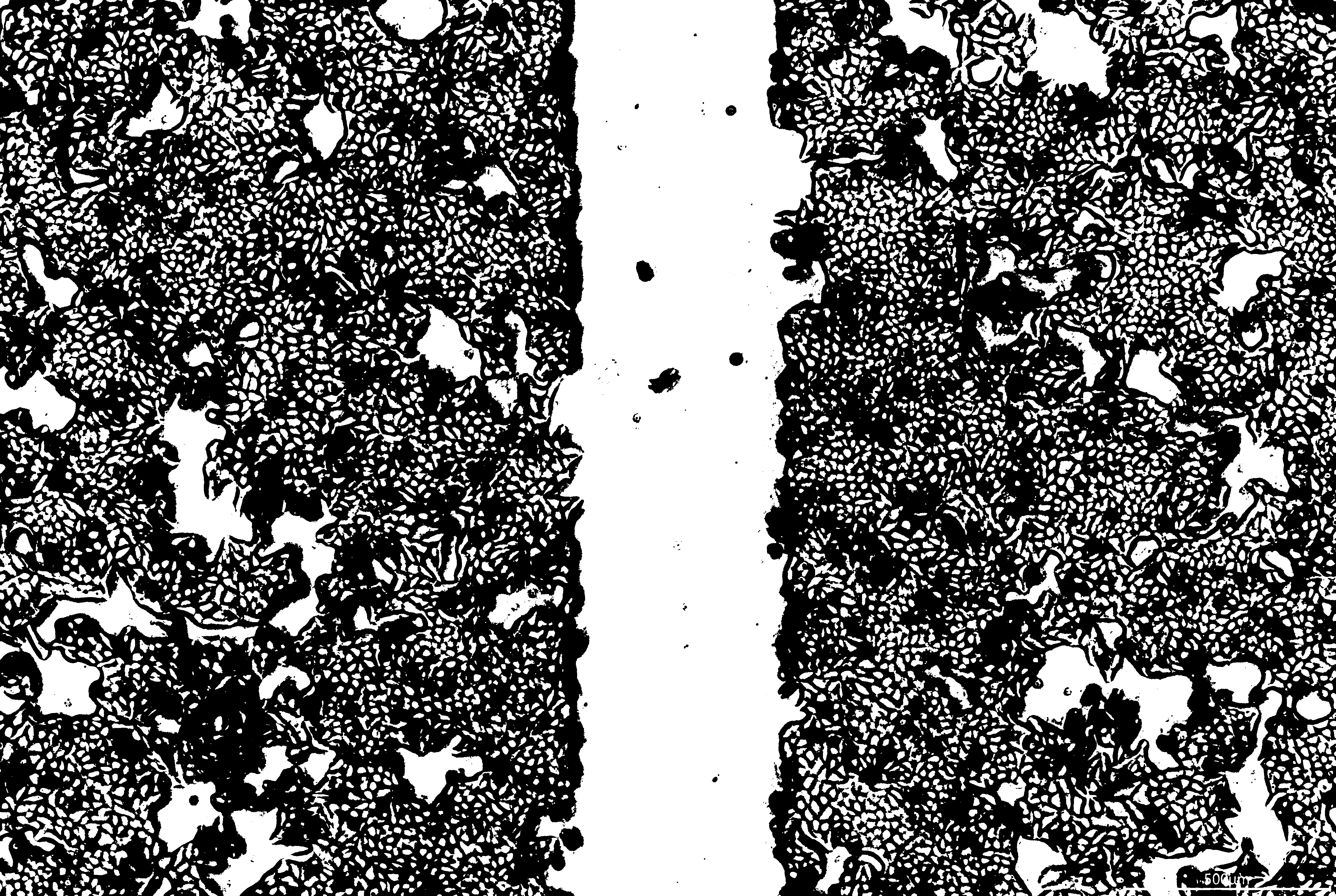

Supplement: Supplemental Information 7 — PZF/PZFX files must be opened using GraphPad Prism. [file peerj-13-19517-s007.zip › FIG 3I/Scratch experiments after imageJ treatment/24.4.8/0003.tif]

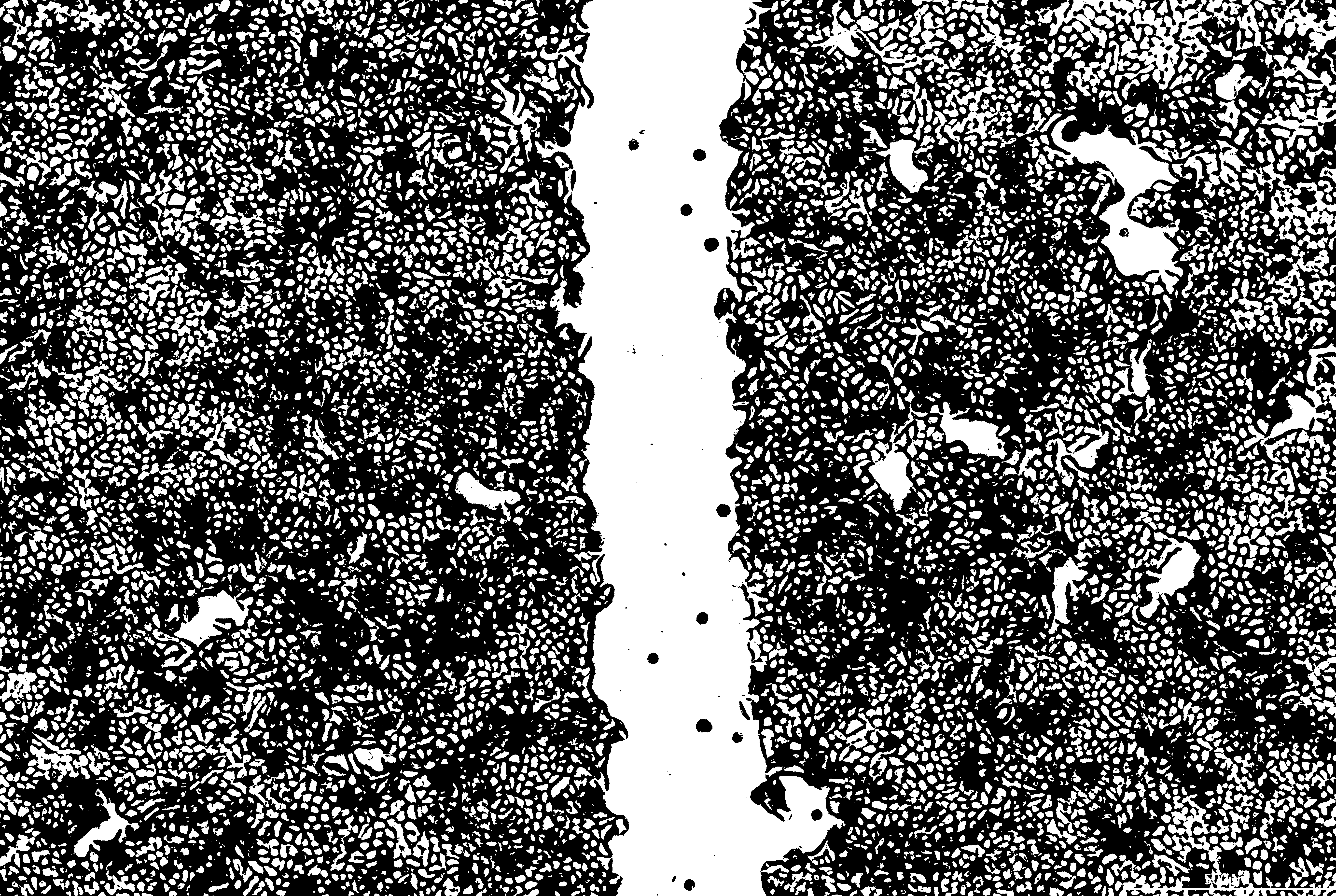

Supplement: Supplemental Information 7 — PZF/PZFX files must be opened using GraphPad Prism. [file peerj-13-19517-s007.zip › FIG 3I/Scratch experiments after imageJ treatment/24.4.8/24H/0001.tif]

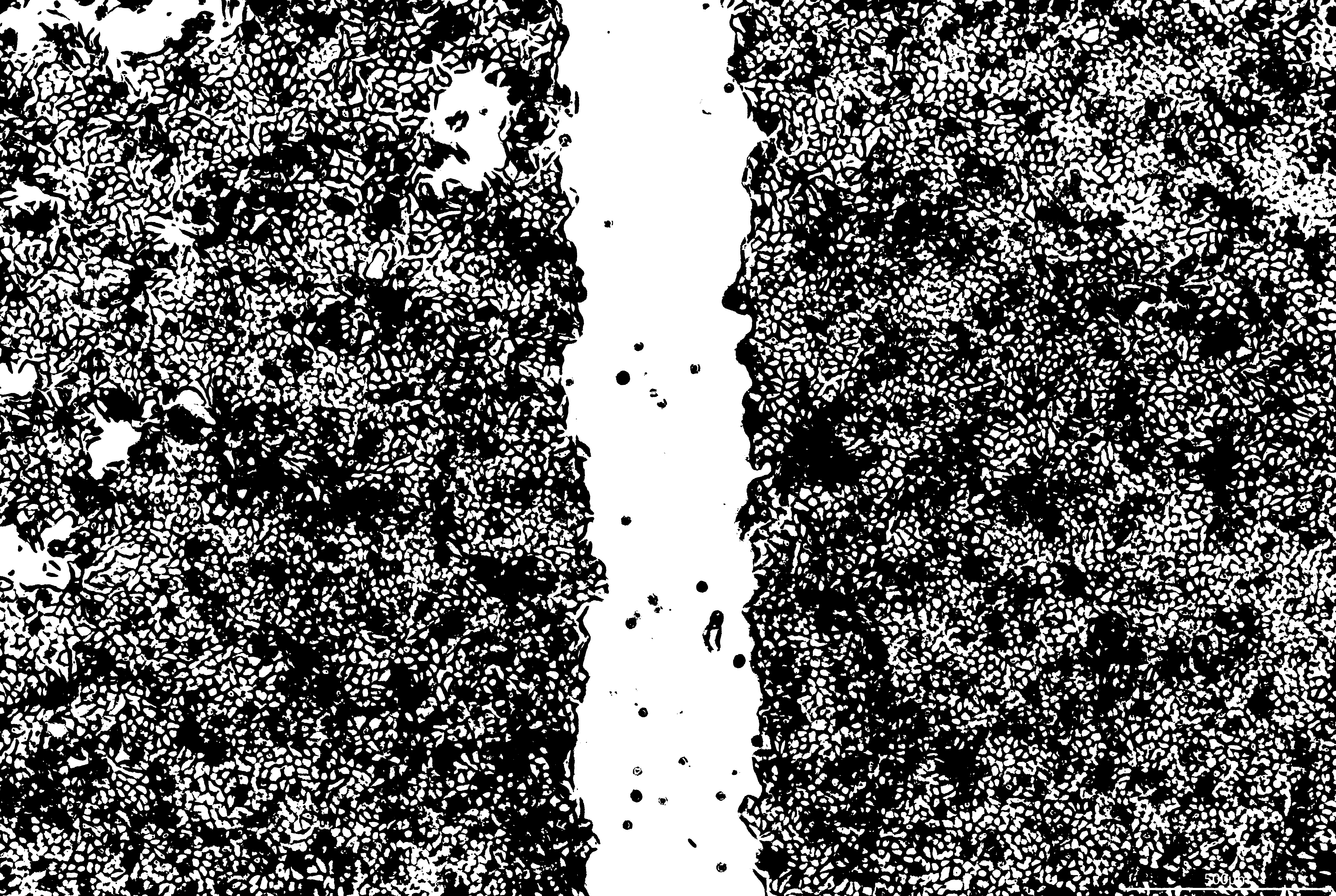

Supplement: Supplemental Information 7 — PZF/PZFX files must be opened using GraphPad Prism. [file peerj-13-19517-s007.zip › FIG 3I/Scratch experiments after imageJ treatment/24.4.8/24H/0002.tif]

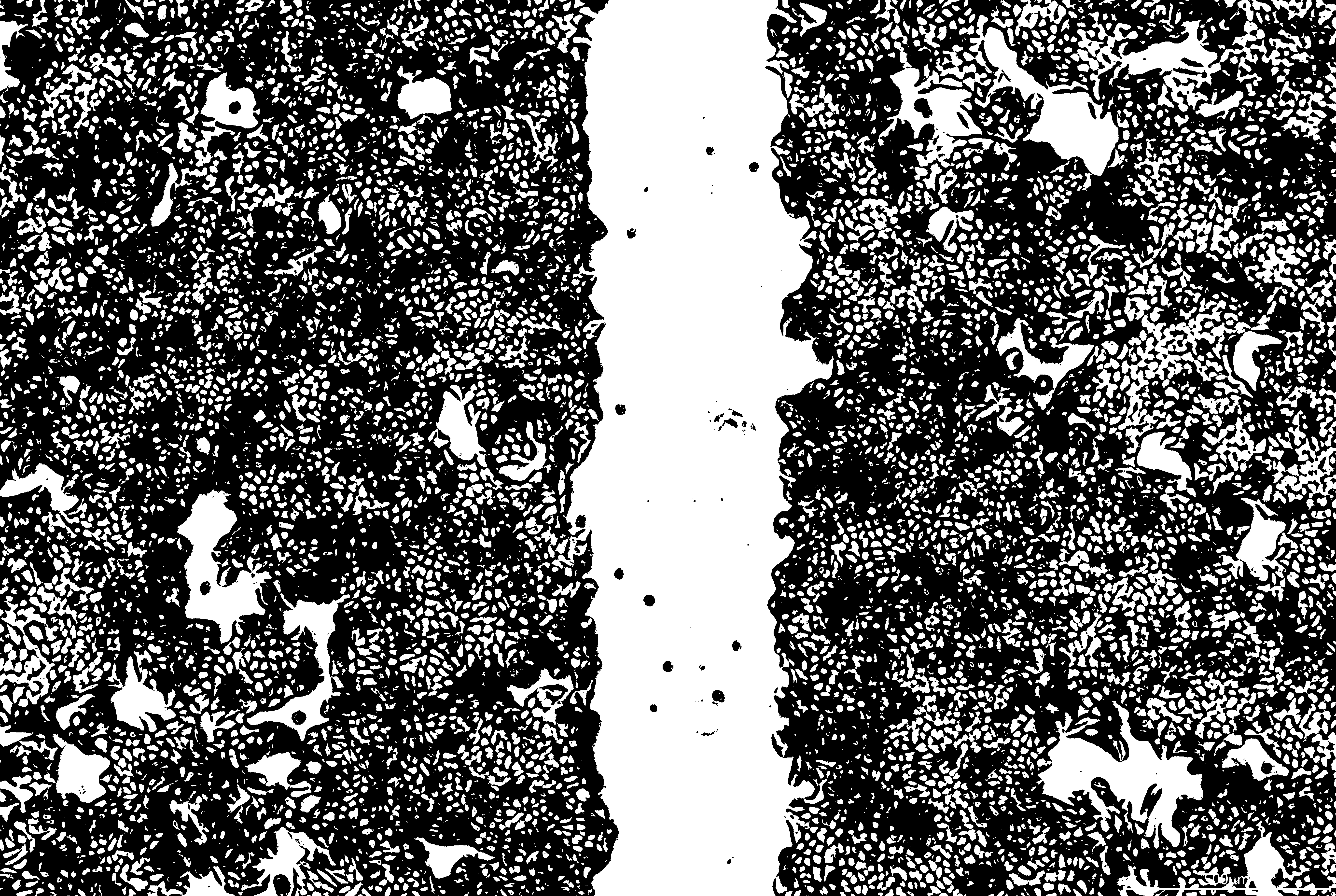

Supplement: Supplemental Information 7 — PZF/PZFX files must be opened using GraphPad Prism. [file peerj-13-19517-s007.zip › FIG 3I/Scratch experiments after imageJ treatment/24.4.8/24H/0003.tif]

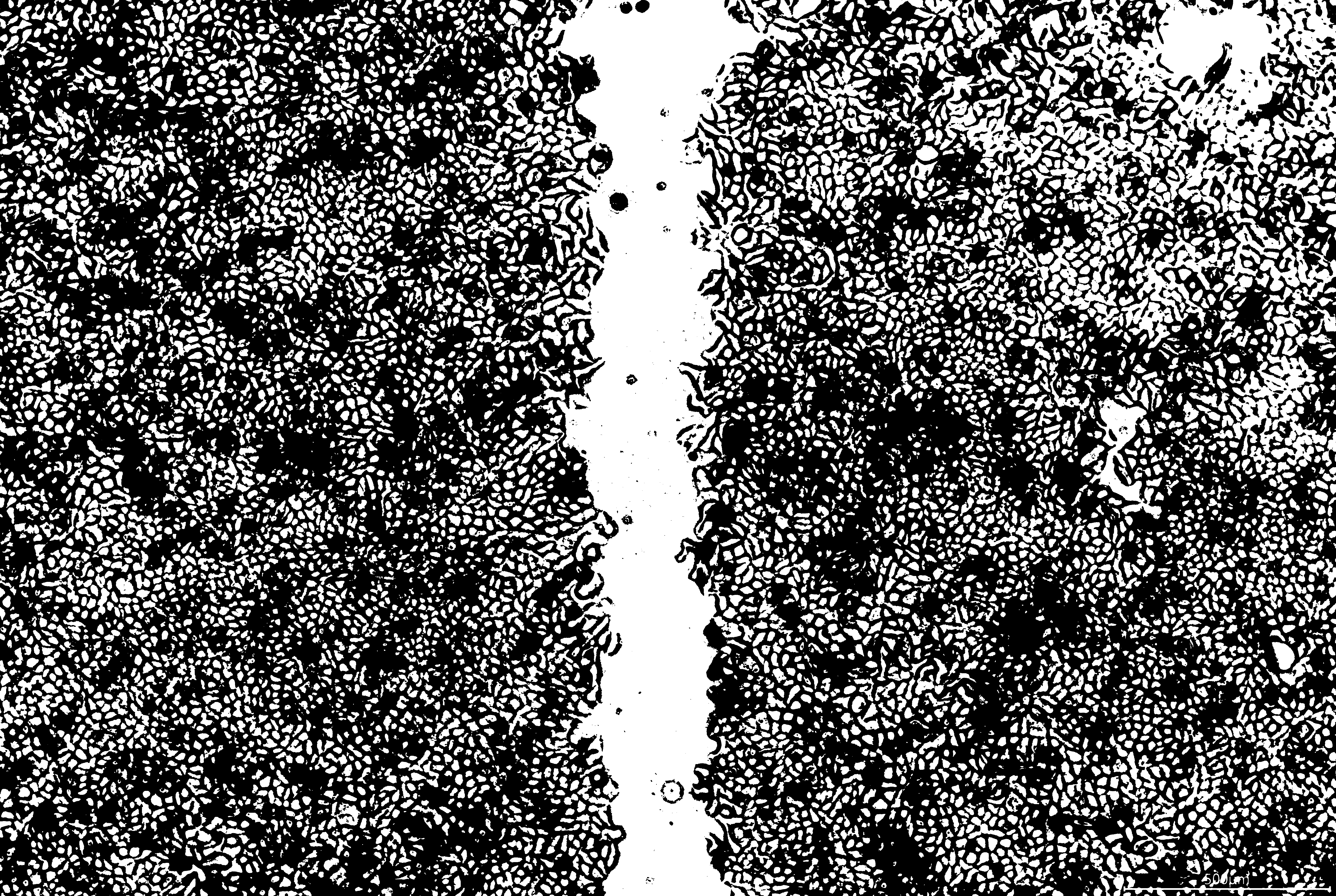

Supplement: Supplemental Information 7 — PZF/PZFX files must be opened using GraphPad Prism. [file peerj-13-19517-s007.zip › FIG 3I/Scratch experiments after imageJ treatment/24.4.8/24H/48H/0001.tif]

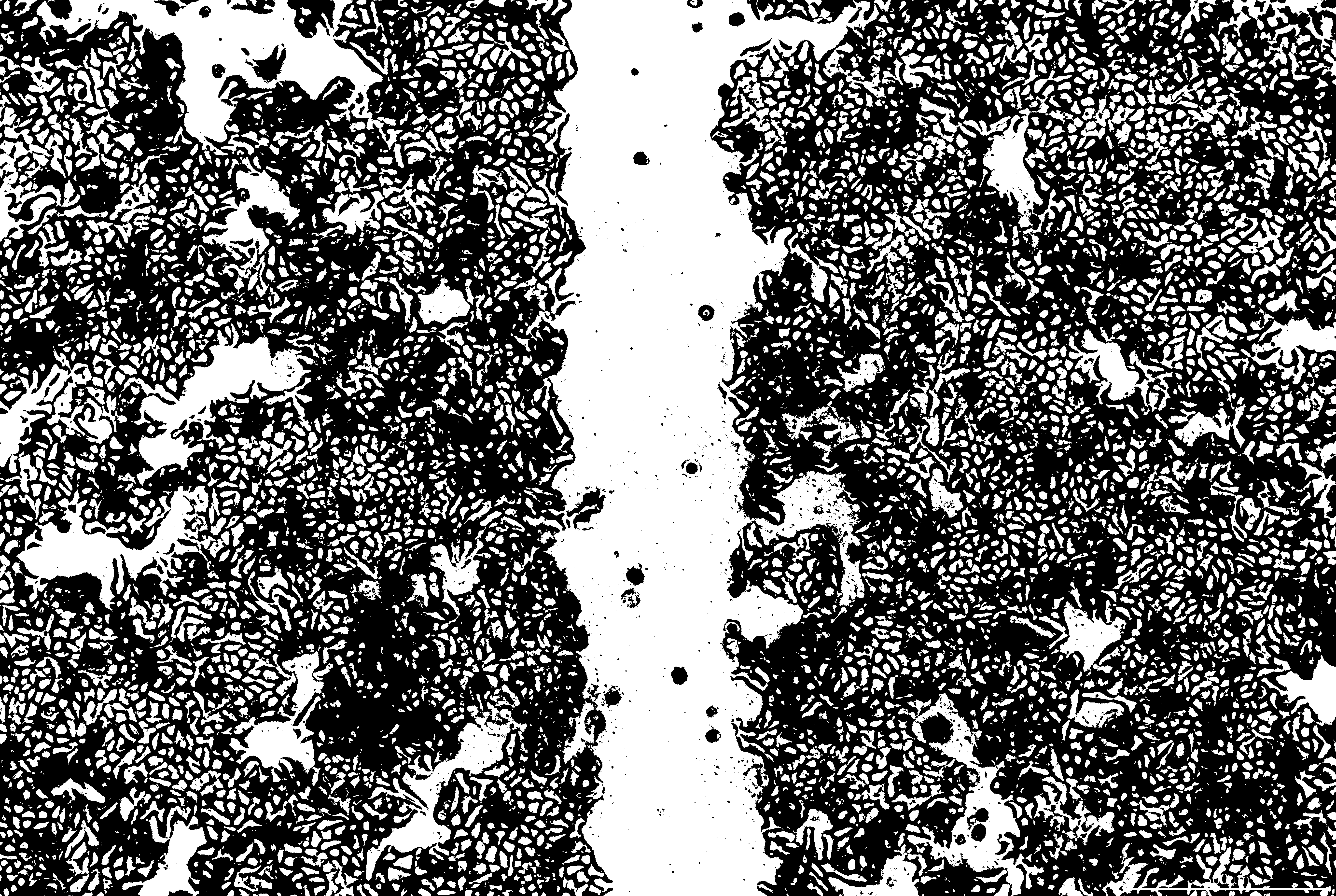

Supplement: Supplemental Information 7 — PZF/PZFX files must be opened using GraphPad Prism. [file peerj-13-19517-s007.zip › FIG 3I/Scratch experiments after imageJ treatment/24.4.8/24H/48H/0002.tif]

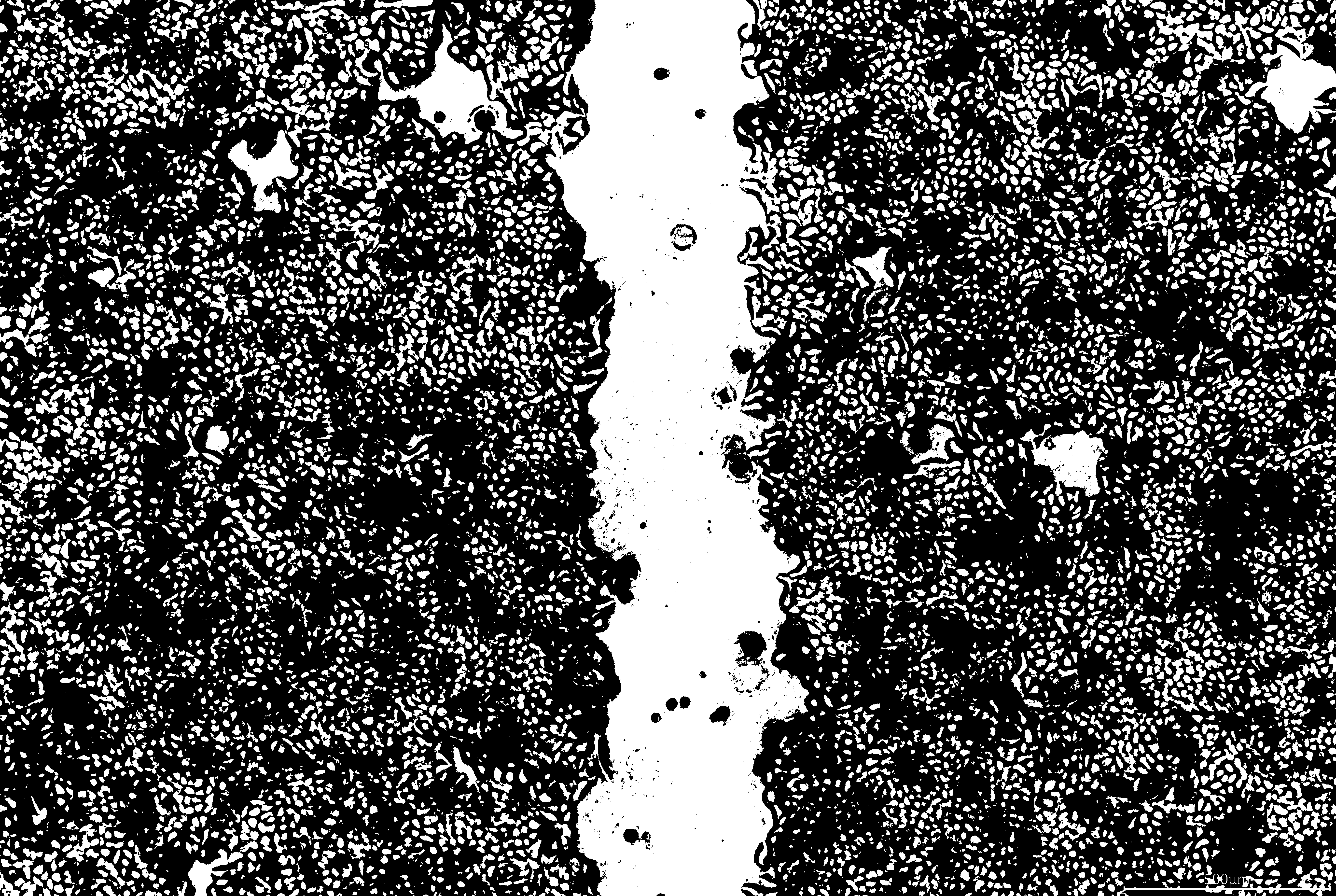

Supplement: Supplemental Information 7 — PZF/PZFX files must be opened using GraphPad Prism. [file peerj-13-19517-s007.zip › FIG 3I/Scratch experiments after imageJ treatment/24.4.8/24H/48H/0003.tif]

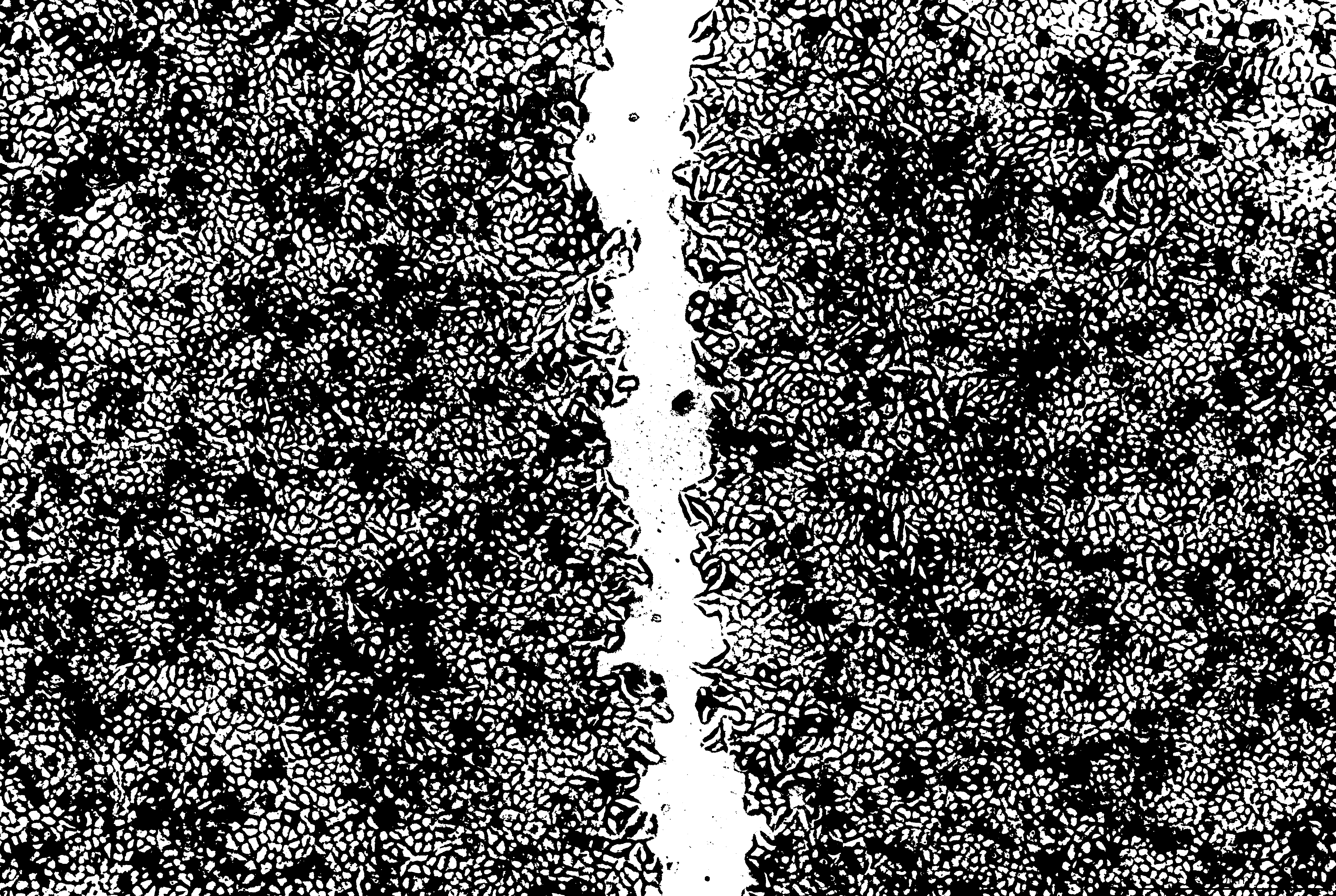

Supplement: Supplemental Information 7 — PZF/PZFX files must be opened using GraphPad Prism. [file peerj-13-19517-s007.zip › FIG 3I/Scratch experiments after imageJ treatment/24.4.8/24H/48H/72H/0001.tif]

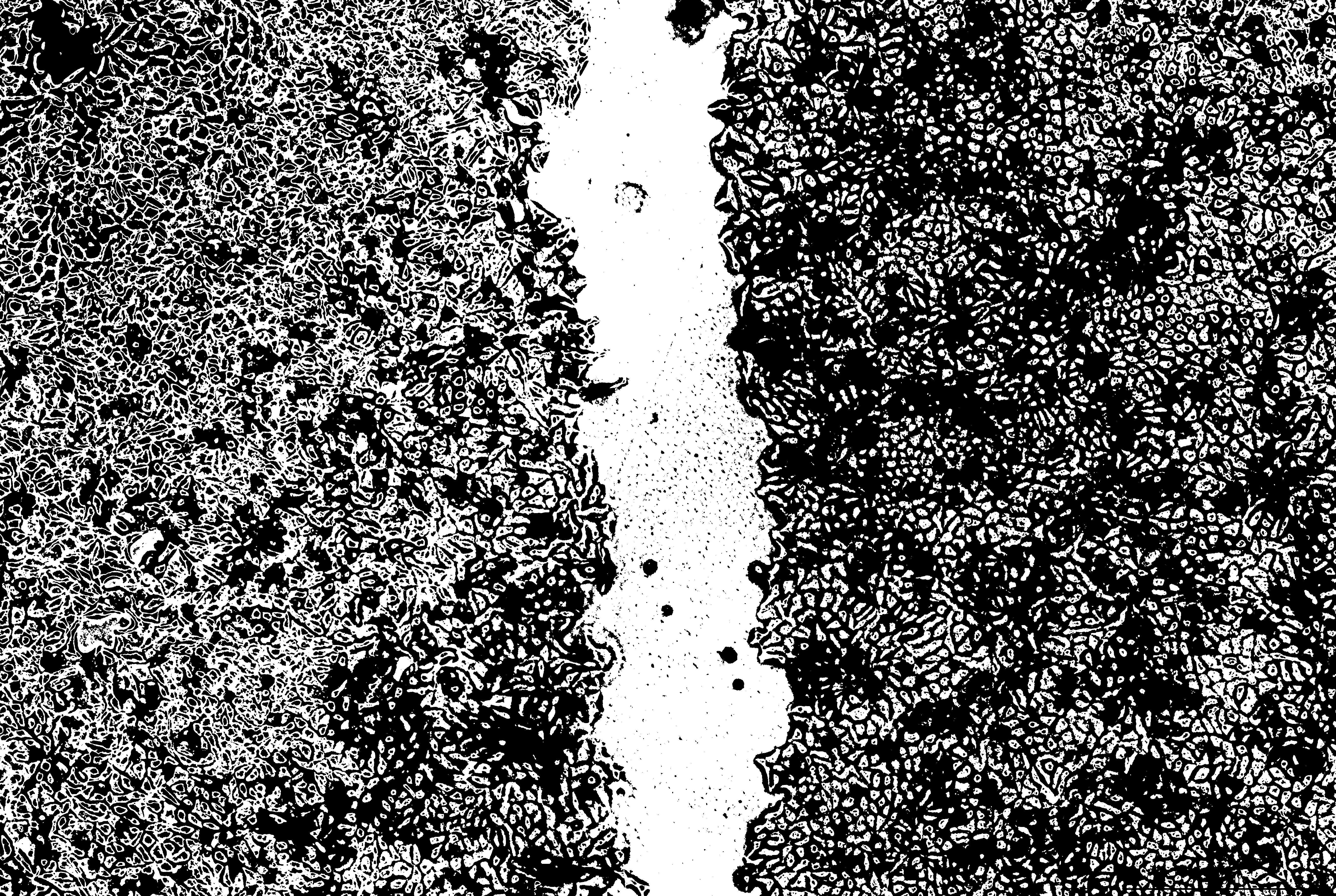

Supplement: Supplemental Information 7 — PZF/PZFX files must be opened using GraphPad Prism. [file peerj-13-19517-s007.zip › FIG 3I/Scratch experiments after imageJ treatment/24.4.8/24H/48H/72H/0002.tif]

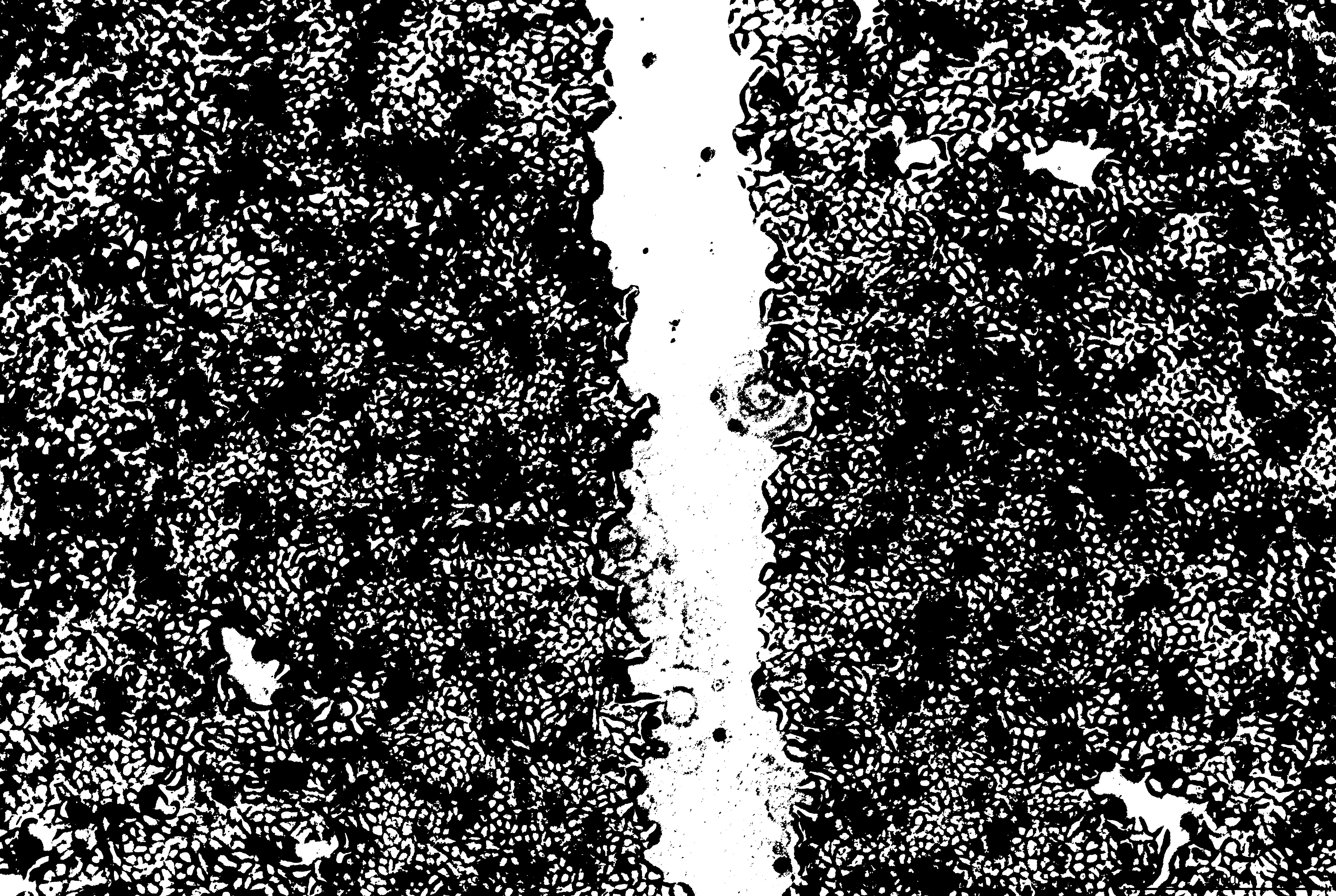

Supplement: Supplemental Information 7 — PZF/PZFX files must be opened using GraphPad Prism. [file peerj-13-19517-s007.zip › FIG 3I/Scratch experiments after imageJ treatment/24.4.8/24H/48H/72H/0003.tif]

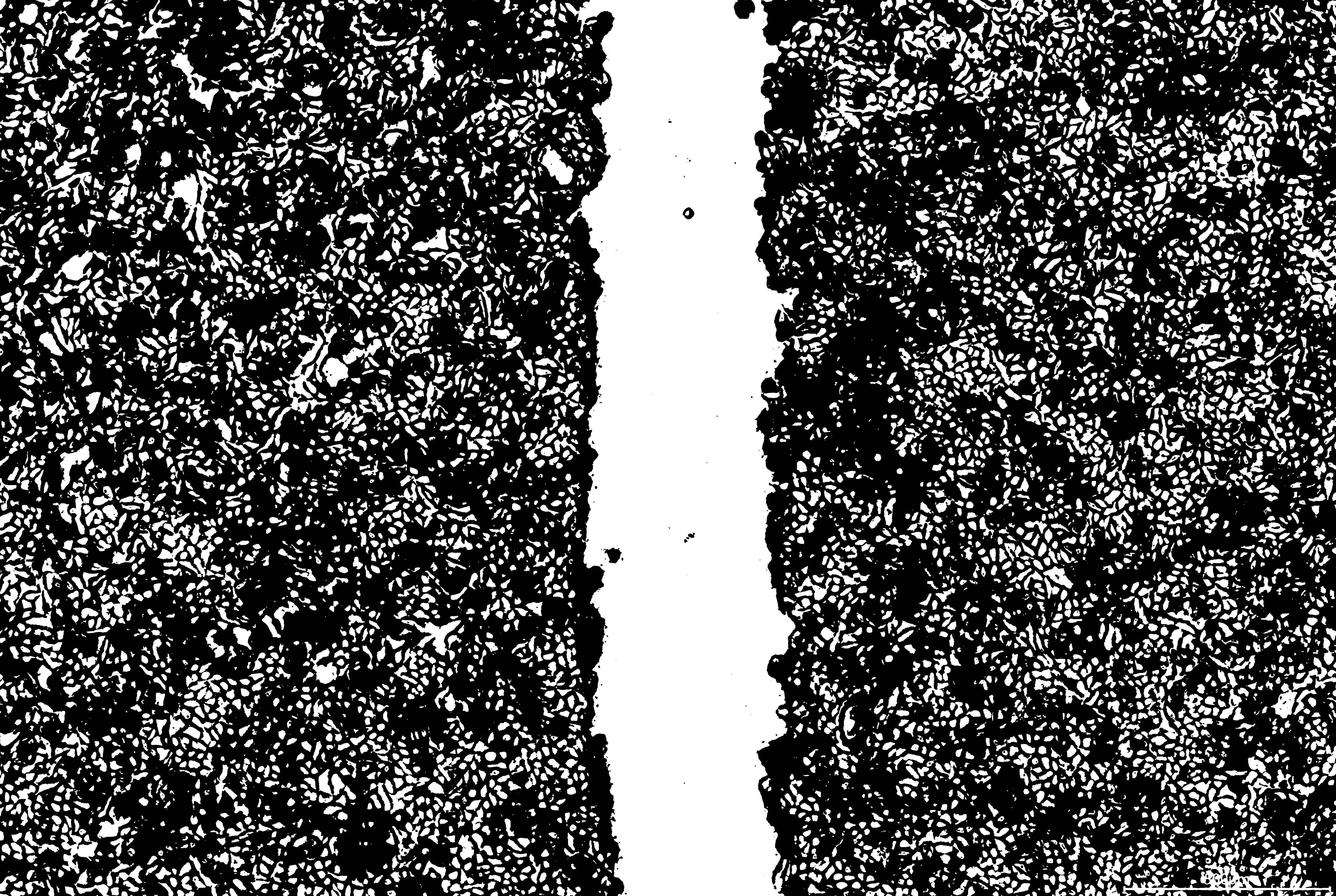

Supplement: Supplemental Information 7 — PZF/PZFX files must be opened using GraphPad Prism. [file peerj-13-19517-s007.zip › FIG 3I/Scratch experiments after imageJ treatment/24.4.9/0001.tif]

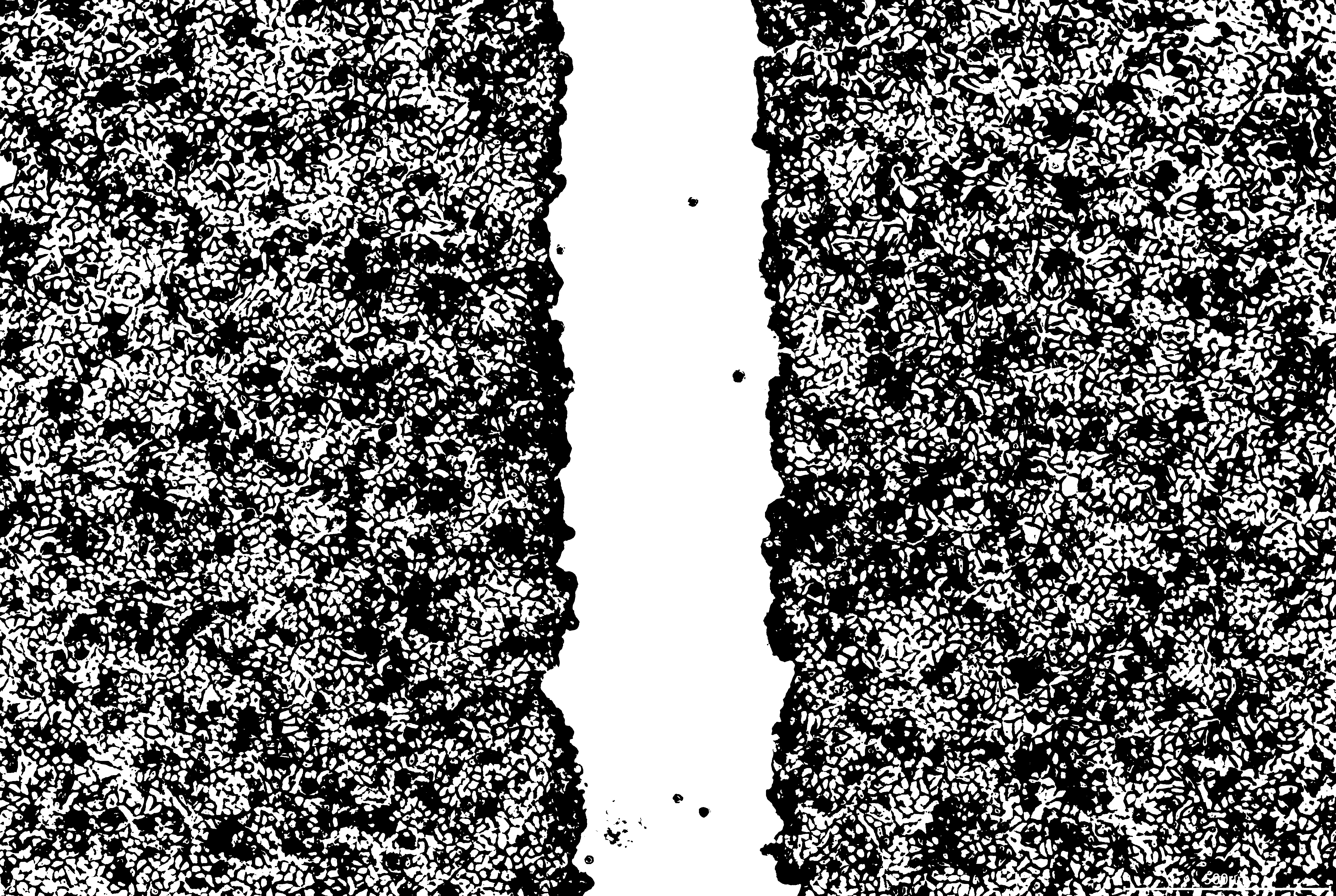

Supplement: Supplemental Information 7 — PZF/PZFX files must be opened using GraphPad Prism. [file peerj-13-19517-s007.zip › FIG 3I/Scratch experiments after imageJ treatment/24.4.9/0002.tif]

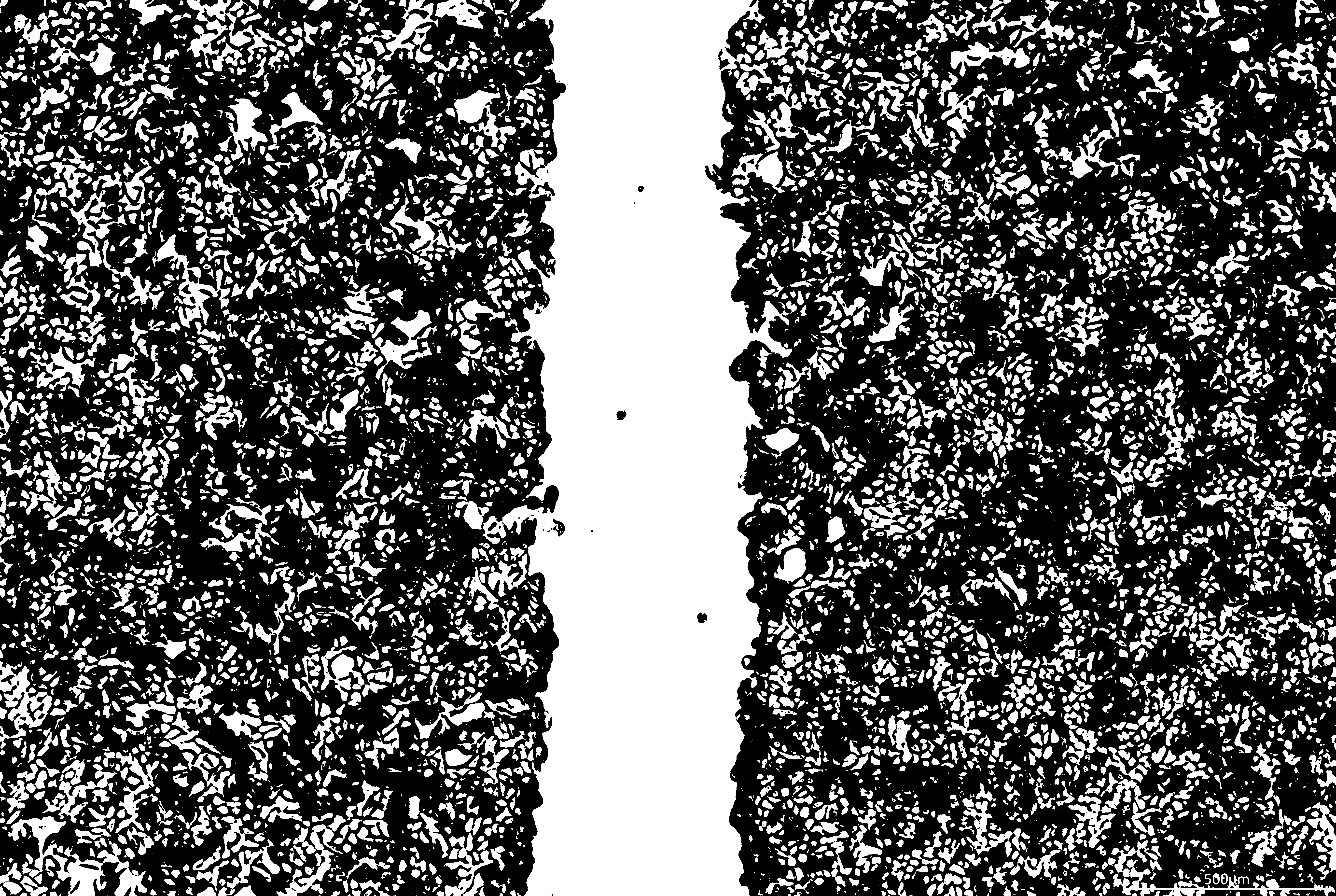

Supplement: Supplemental Information 7 — PZF/PZFX files must be opened using GraphPad Prism. [file peerj-13-19517-s007.zip › FIG 3I/Scratch experiments after imageJ treatment/24.4.9/0003.tif]

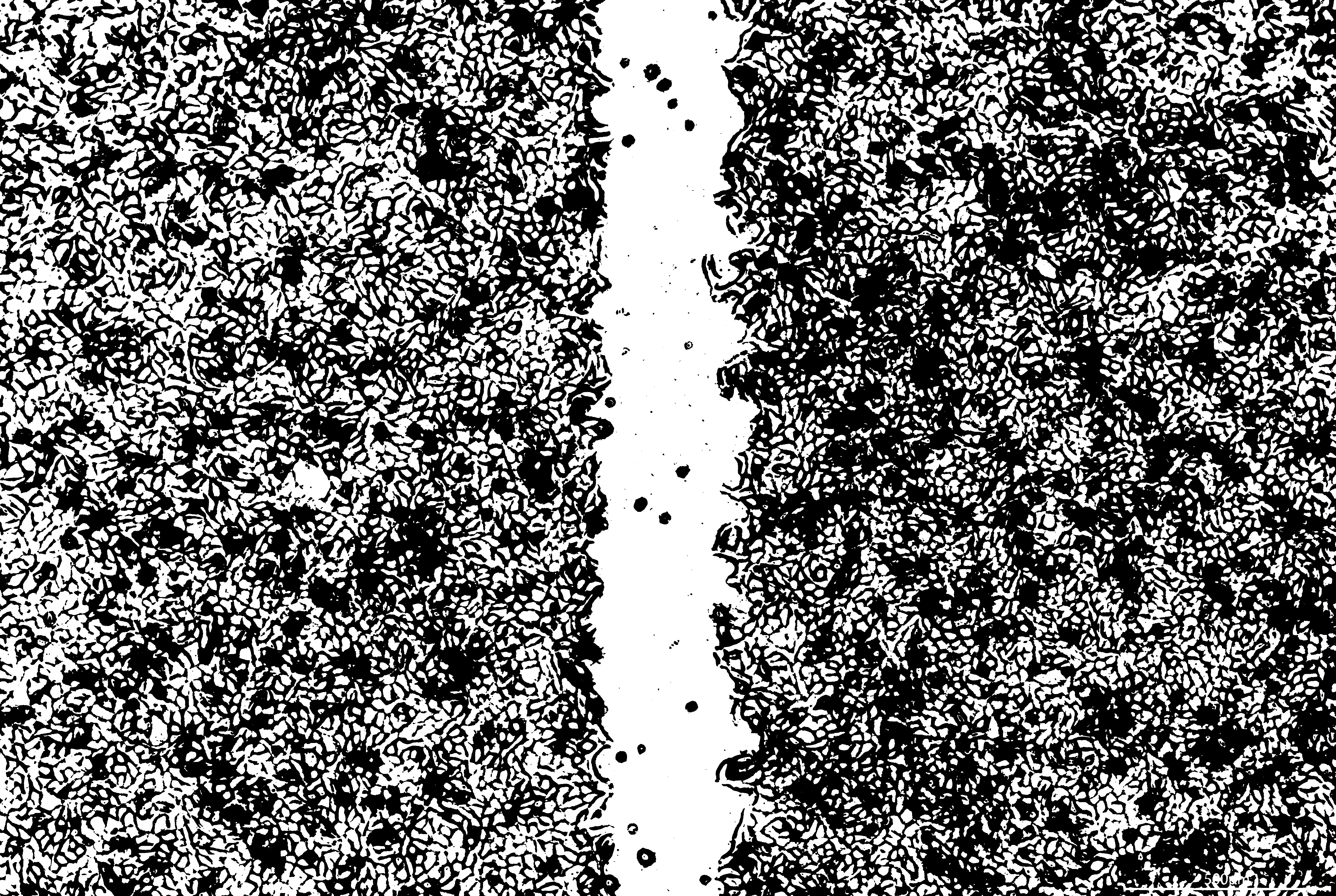

Supplement: Supplemental Information 7 — PZF/PZFX files must be opened using GraphPad Prism. [file peerj-13-19517-s007.zip › FIG 3I/Scratch experiments after imageJ treatment/24.4.9/24H/0001.tif]

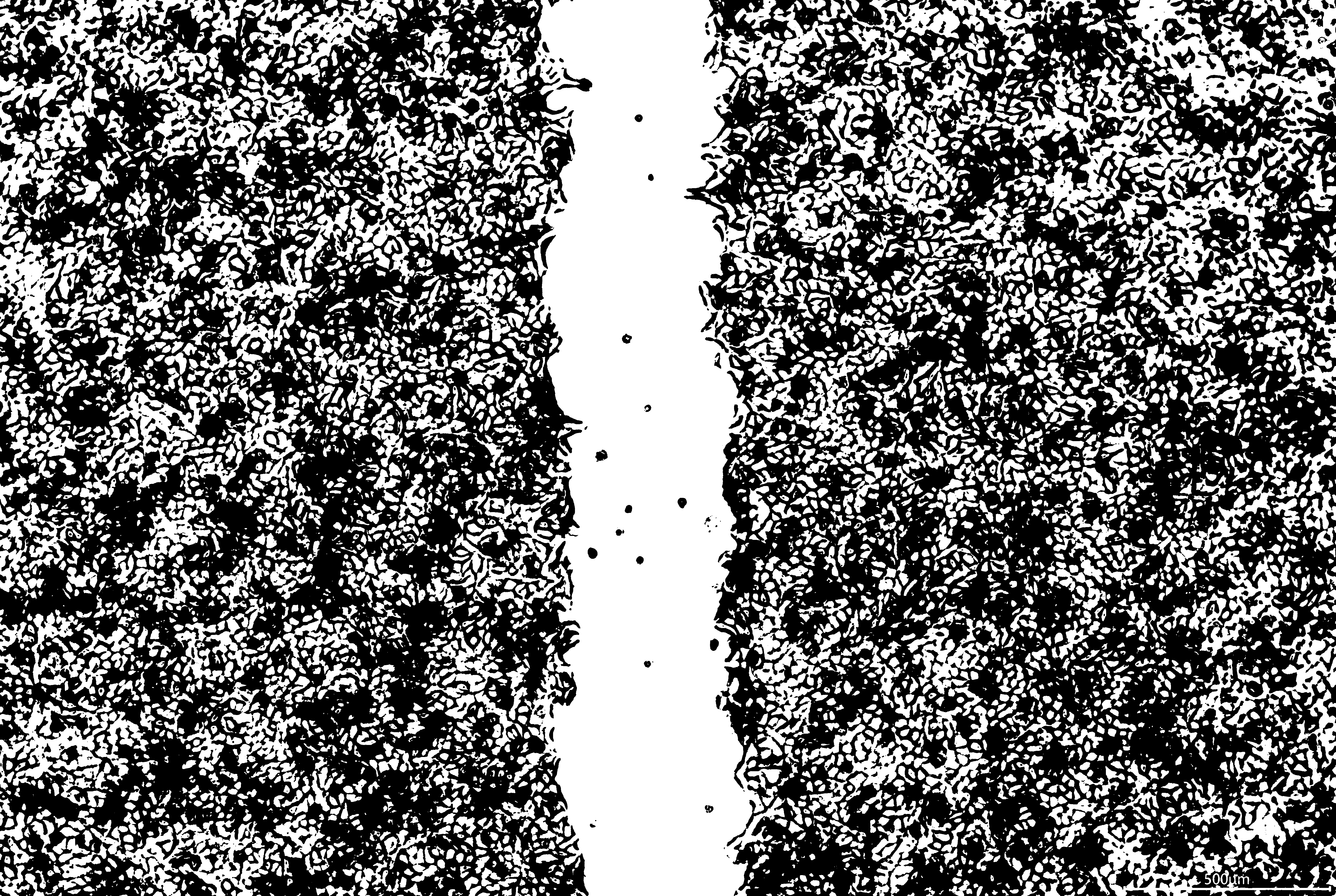

Supplement: Supplemental Information 7 — PZF/PZFX files must be opened using GraphPad Prism. [file peerj-13-19517-s007.zip › FIG 3I/Scratch experiments after imageJ treatment/24.4.9/24H/0002.tif]

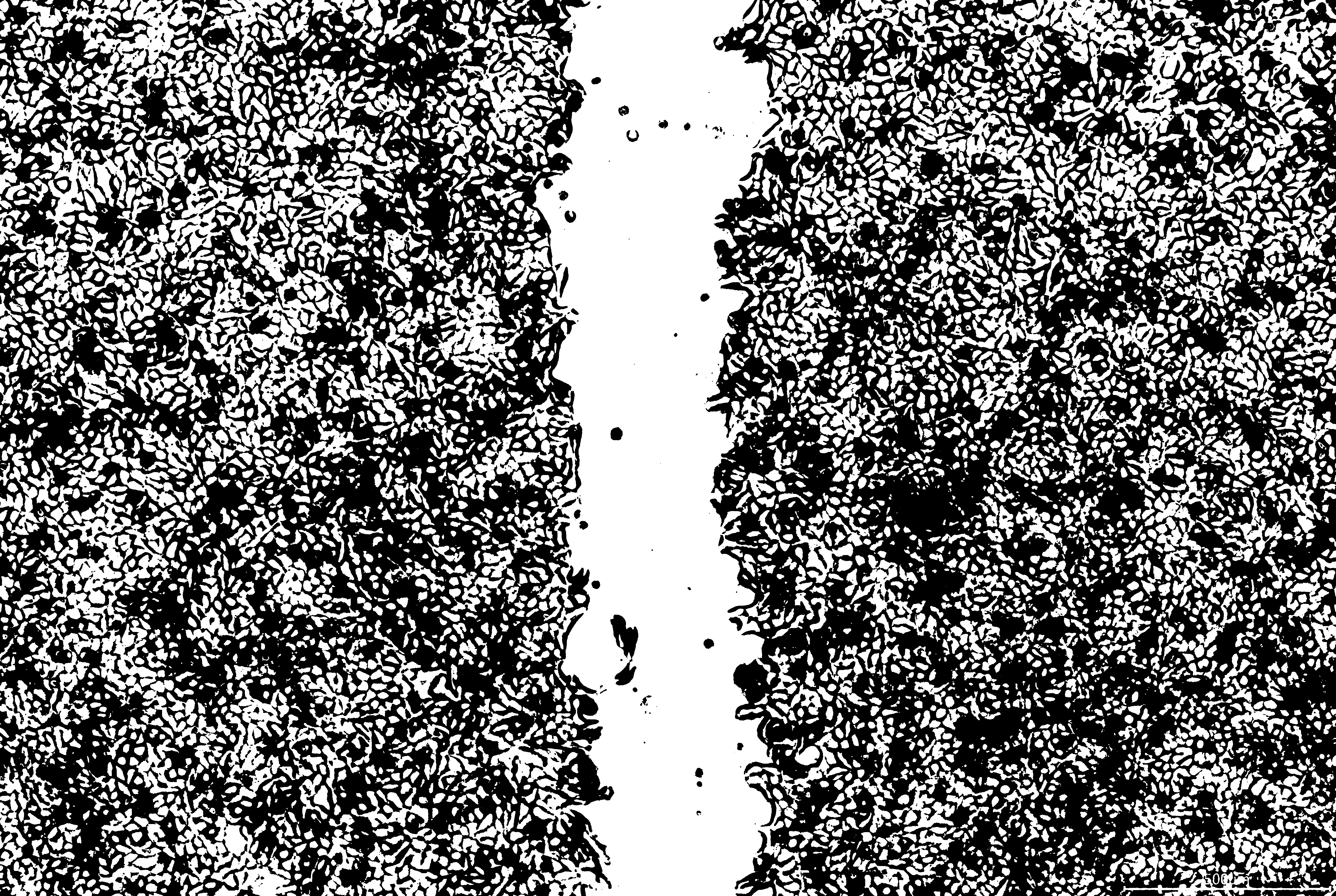

Supplement: Supplemental Information 7 — PZF/PZFX files must be opened using GraphPad Prism. [file peerj-13-19517-s007.zip › FIG 3I/Scratch experiments after imageJ treatment/24.4.9/24H/0003.tif]

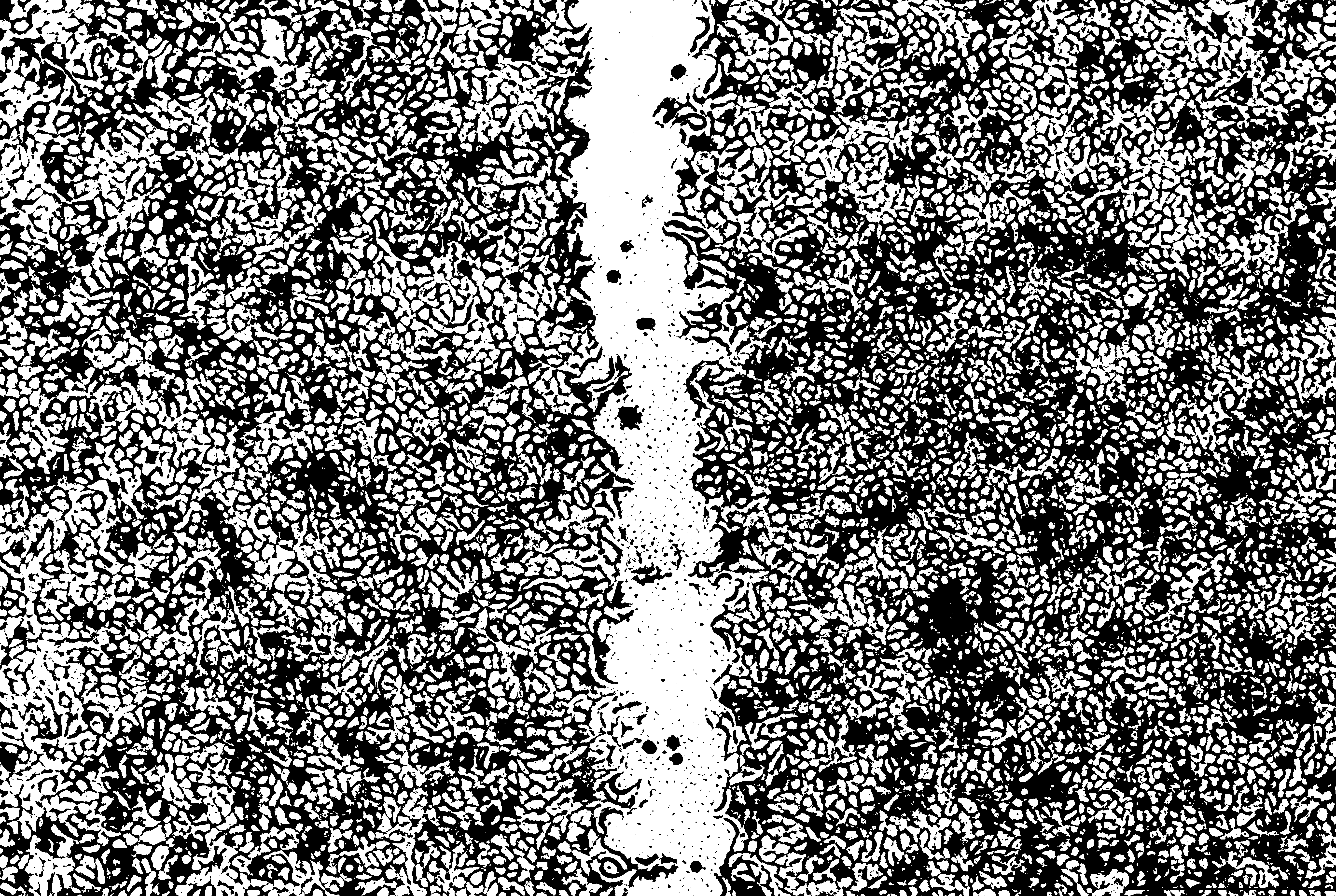

Supplement: Supplemental Information 7 — PZF/PZFX files must be opened using GraphPad Prism. [file peerj-13-19517-s007.zip › FIG 3I/Scratch experiments after imageJ treatment/24.4.9/24H/48H/0001.tif]

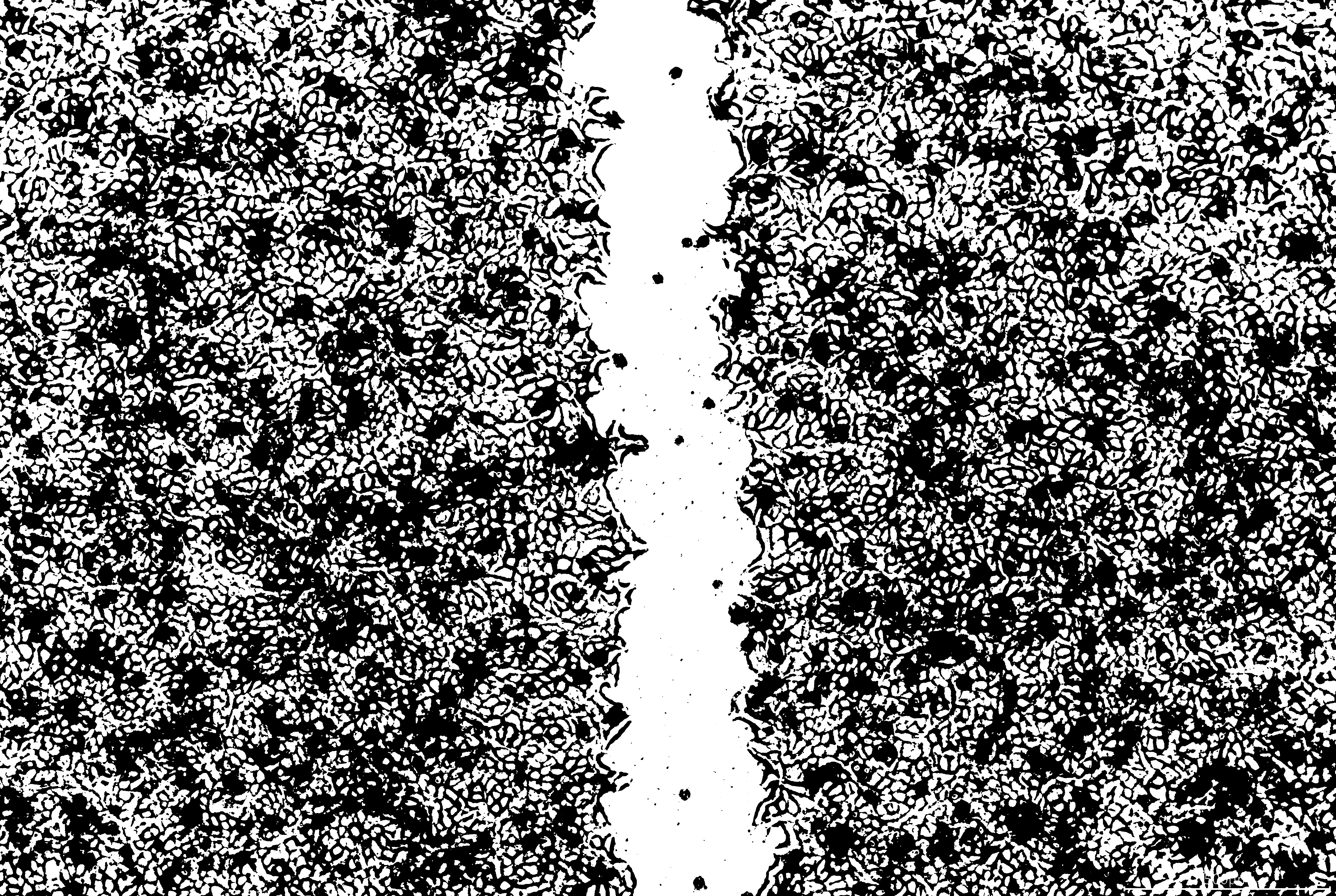

Supplement: Supplemental Information 7 — PZF/PZFX files must be opened using GraphPad Prism. [file peerj-13-19517-s007.zip › FIG 3I/Scratch experiments after imageJ treatment/24.4.9/24H/48H/0002.tif]

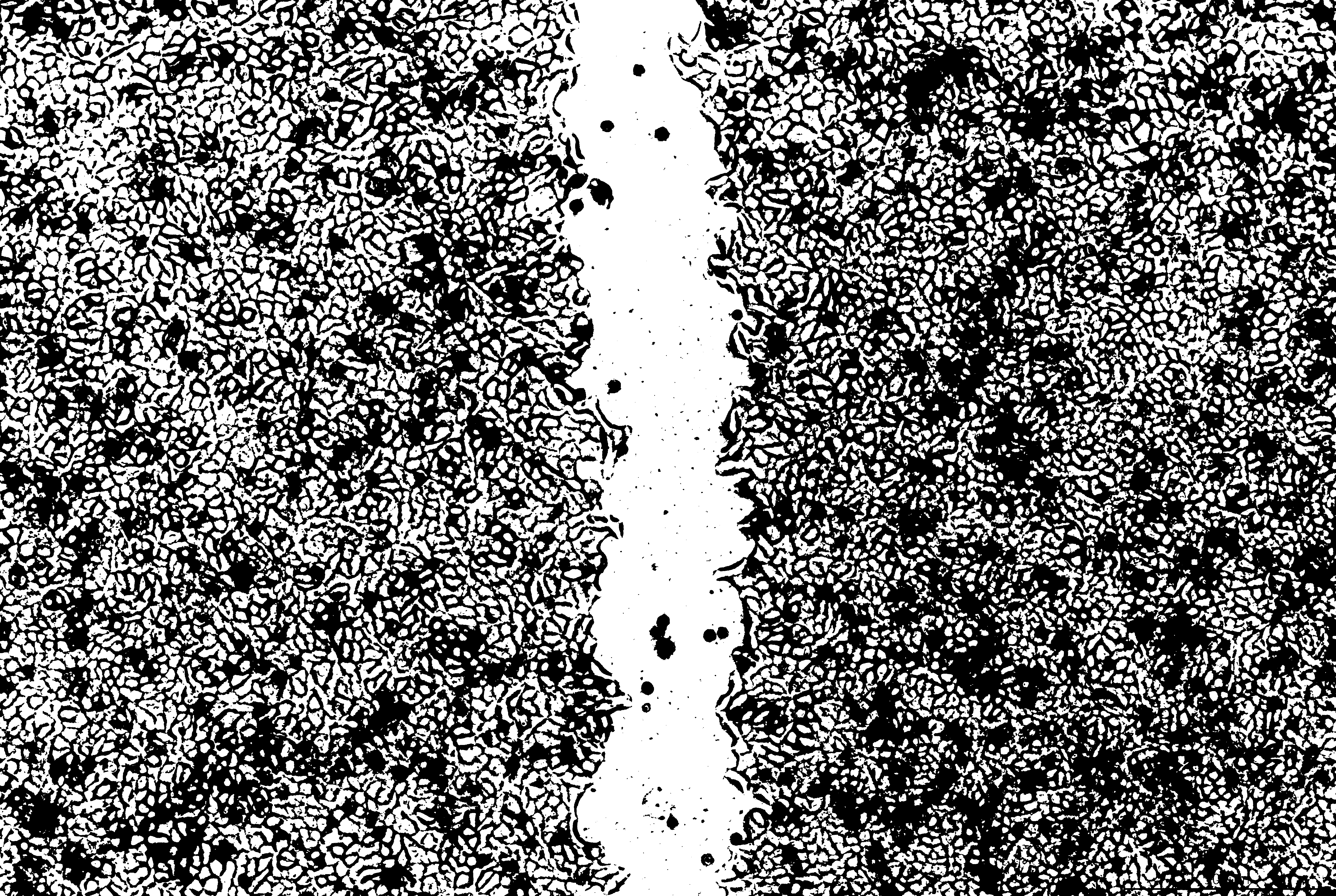

Supplement: Supplemental Information 7 — PZF/PZFX files must be opened using GraphPad Prism. [file peerj-13-19517-s007.zip › FIG 3I/Scratch experiments after imageJ treatment/24.4.9/24H/48H/0003.tif]

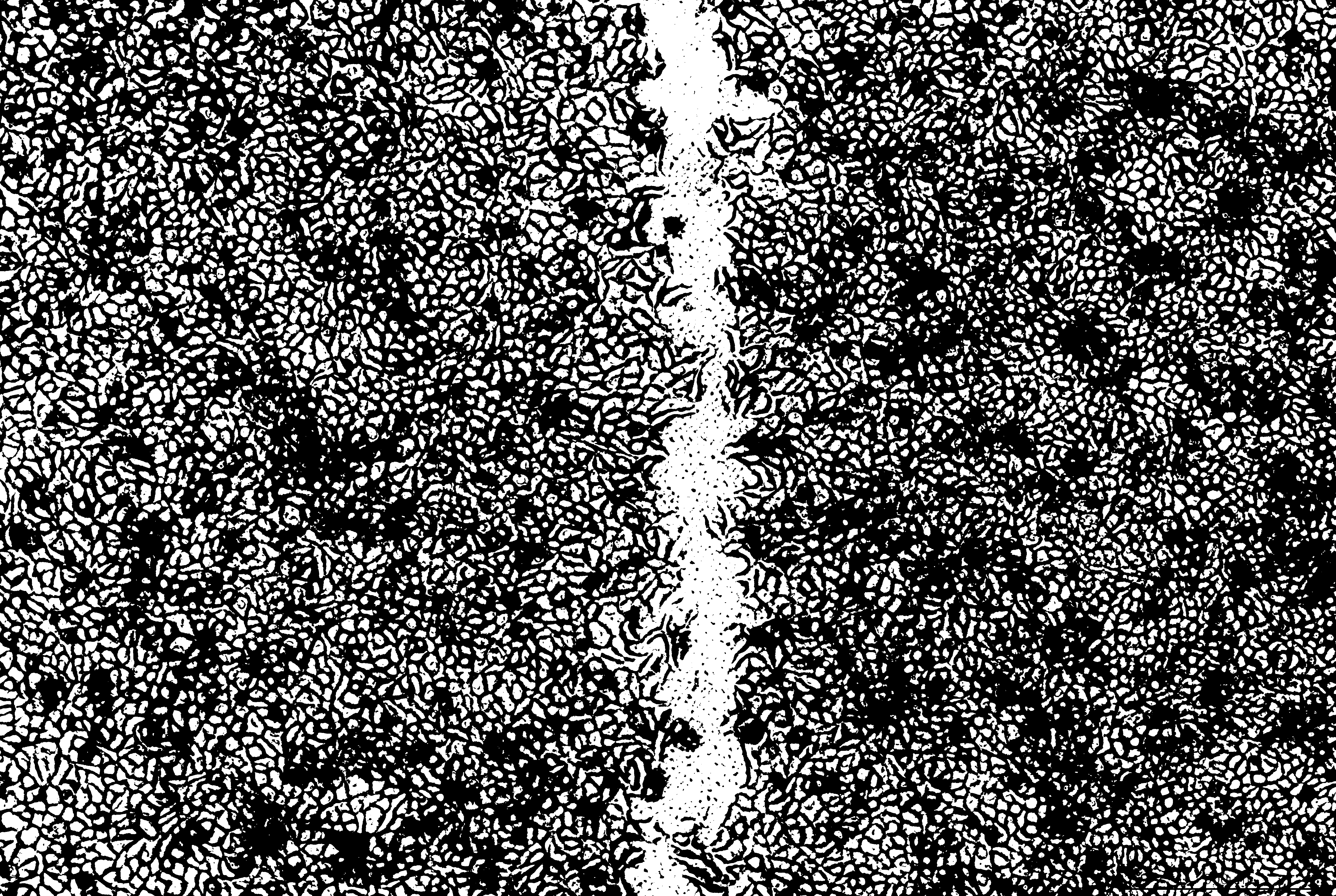

Supplement: Supplemental Information 7 — PZF/PZFX files must be opened using GraphPad Prism. [file peerj-13-19517-s007.zip › FIG 3I/Scratch experiments after imageJ treatment/24.4.9/24H/48H/72H/0001.tif]

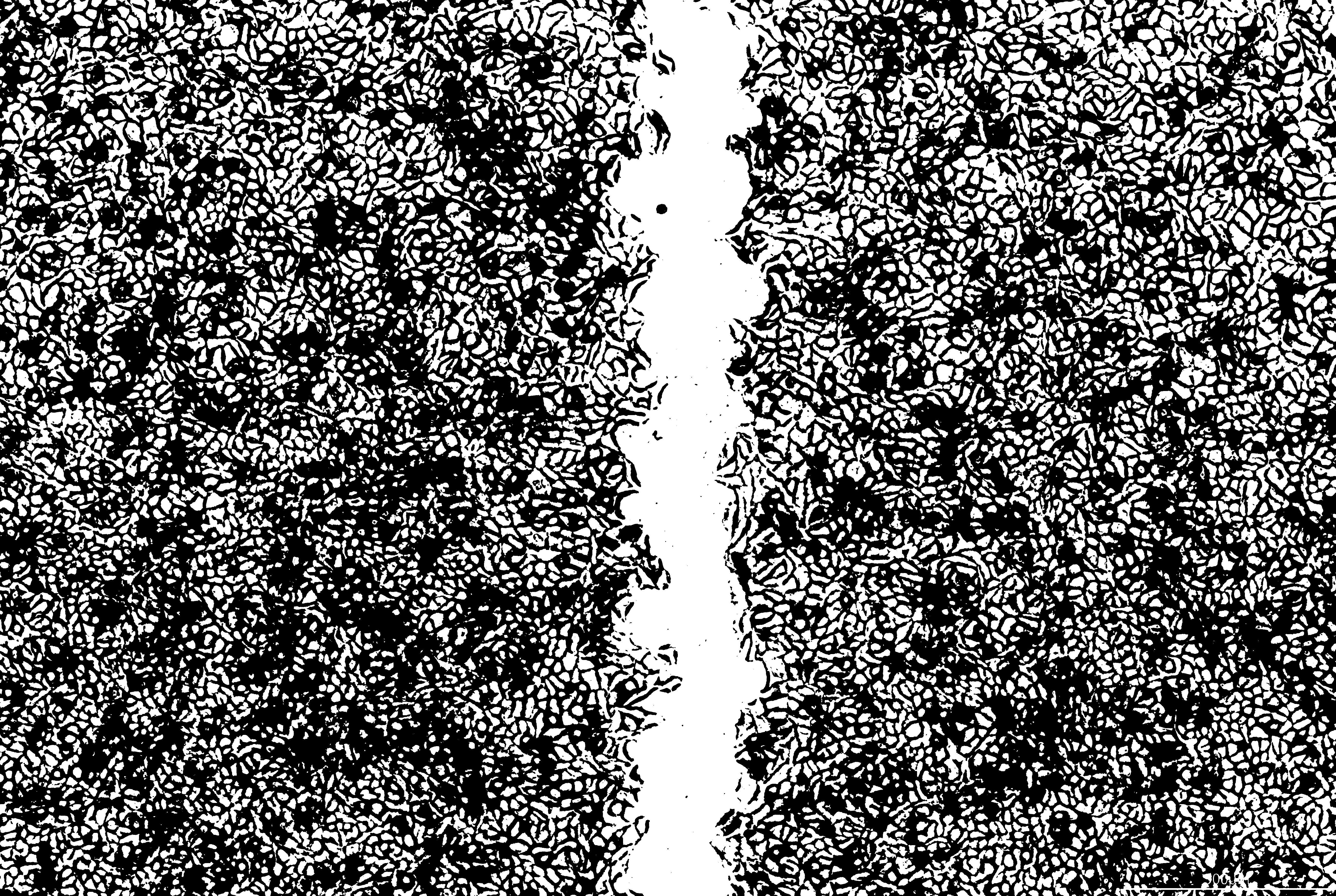

Supplement: Supplemental Information 7 — PZF/PZFX files must be opened using GraphPad Prism. [file peerj-13-19517-s007.zip › FIG 3I/Scratch experiments after imageJ treatment/24.4.9/24H/48H/72H/0002.tif]

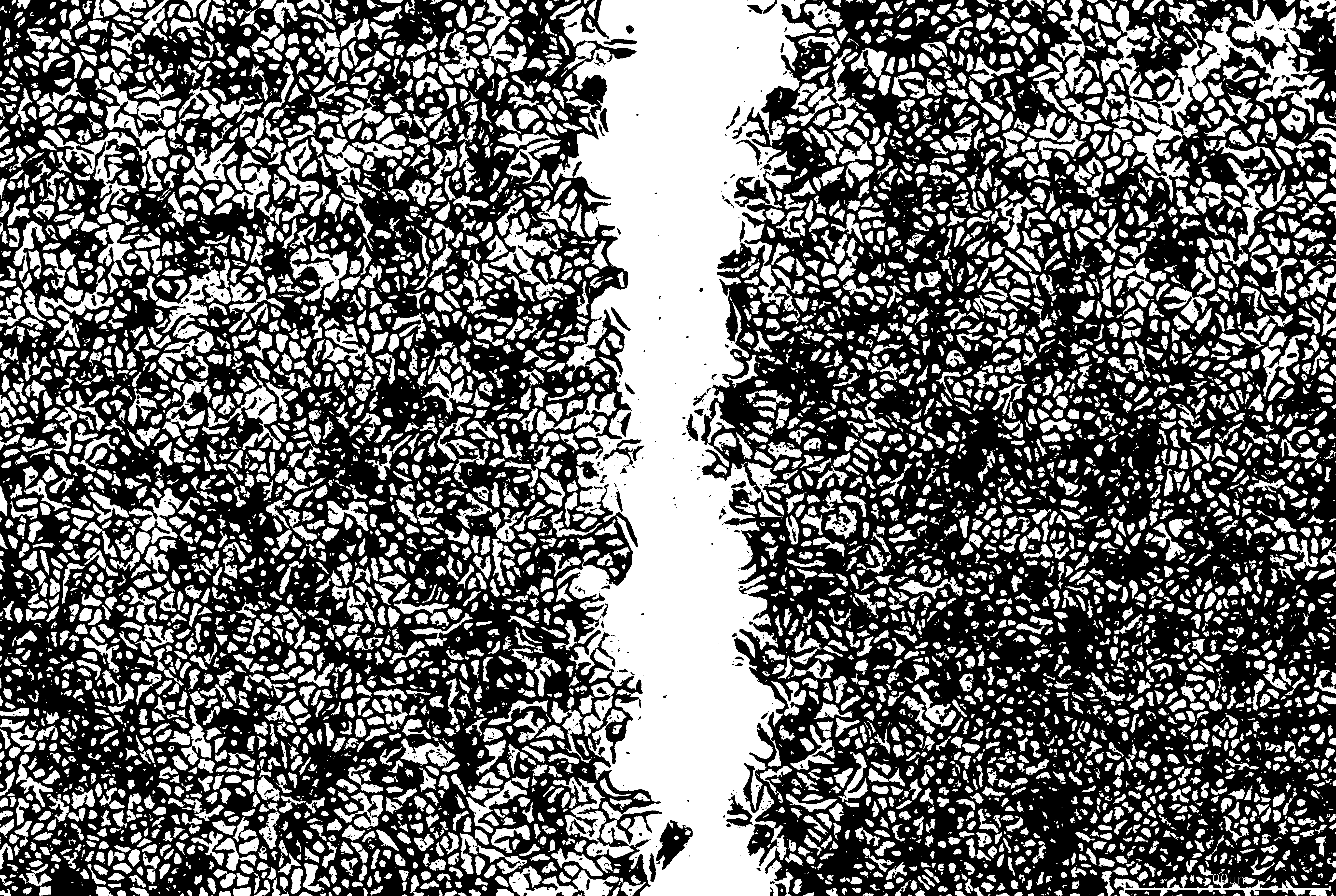

Supplement: Supplemental Information 7 — PZF/PZFX files must be opened using GraphPad Prism. [file peerj-13-19517-s007.zip › FIG 3I/Scratch experiments after imageJ treatment/24.4.9/24H/48H/72H/0003.tif]

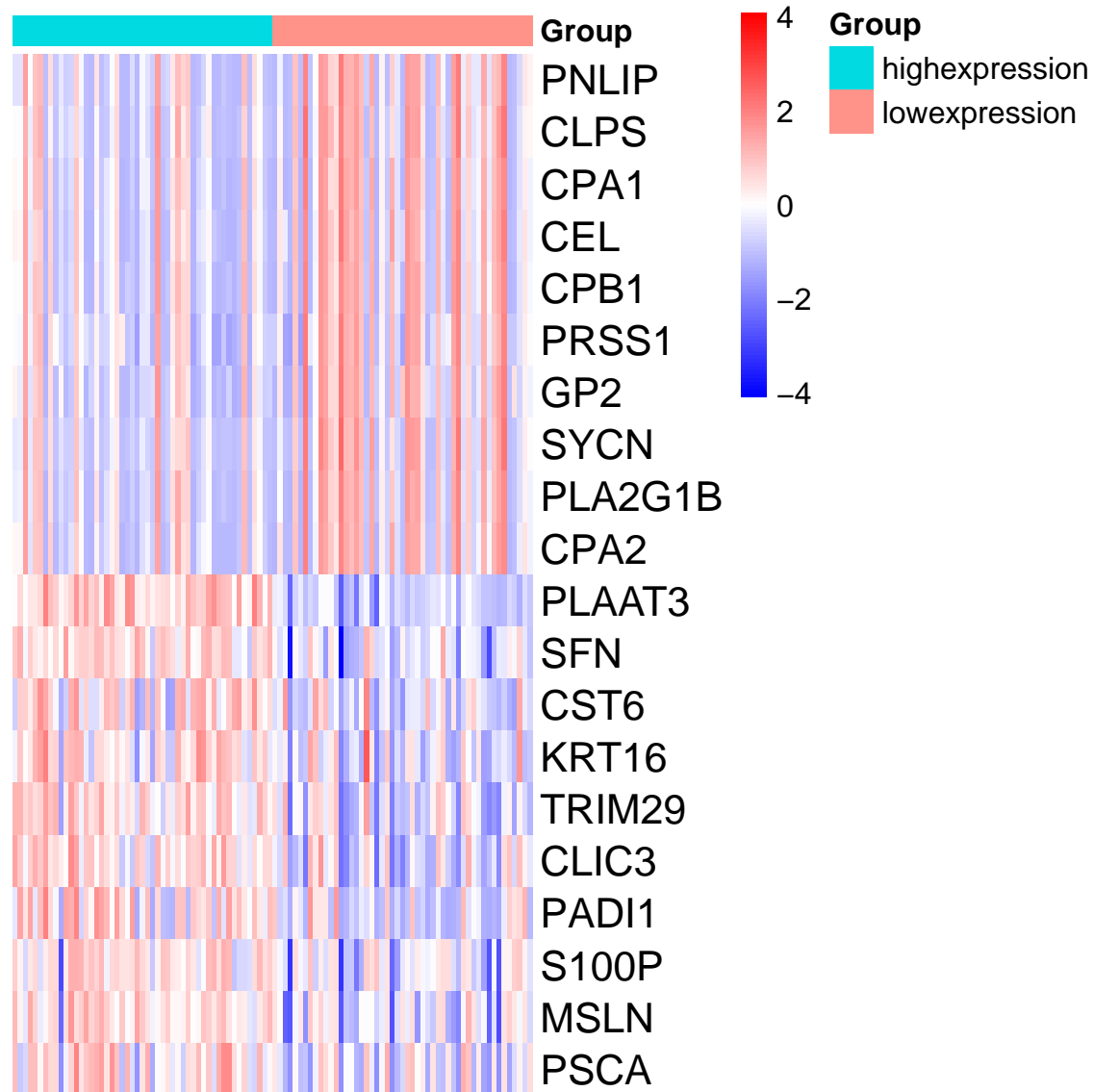

Supplement: Supplemental Information 10 — PZF/PZFX files must be opened using GraphPad Prism. [file peerj-13-19517-s010.zip › FIG 4B-F/FIG 4B/FIG 4B.pdf]

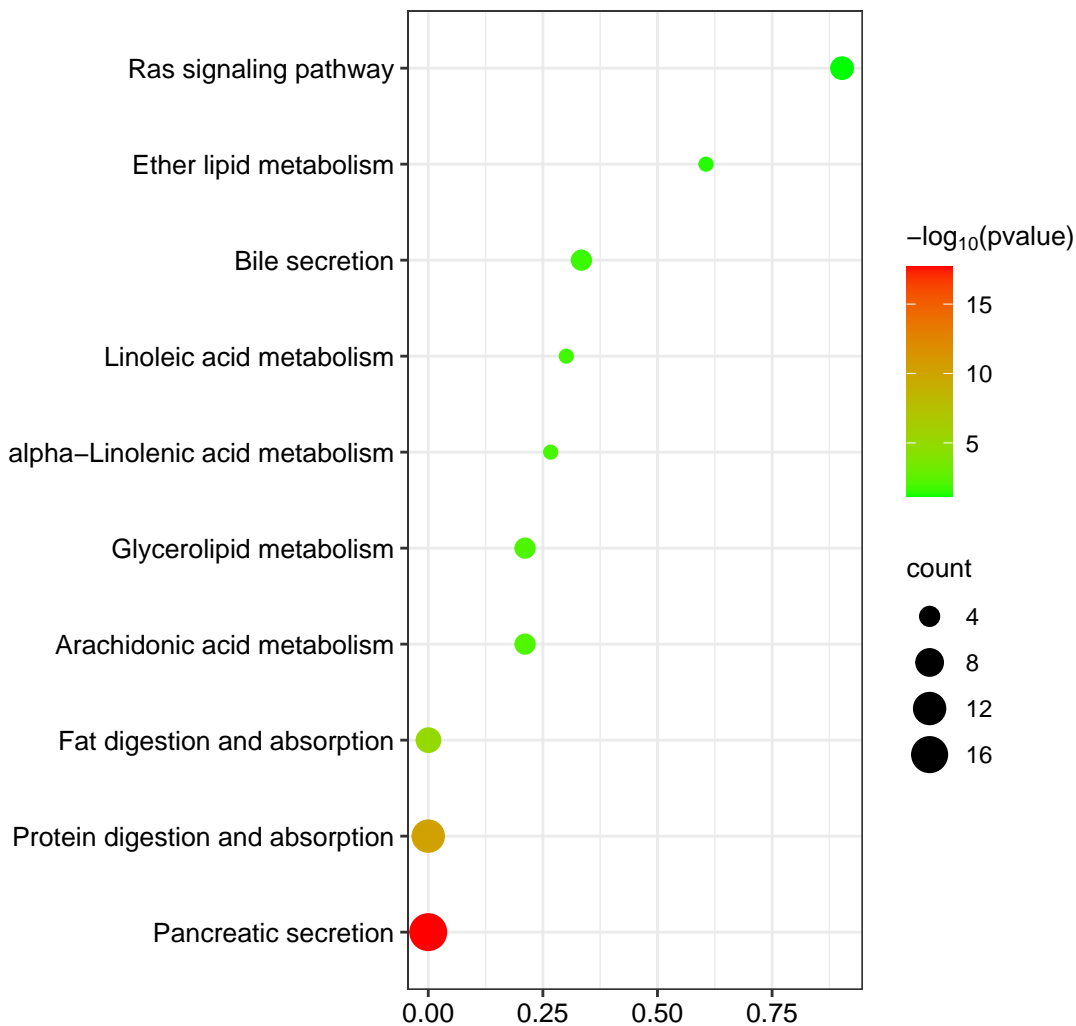

Supplement: Supplemental Information 10 — PZF/PZFX files must be opened using GraphPad Prism. [file peerj-13-19517-s010.zip › FIG 4B-F/FIG 4C/KEGG_PATHWAY.pdf]

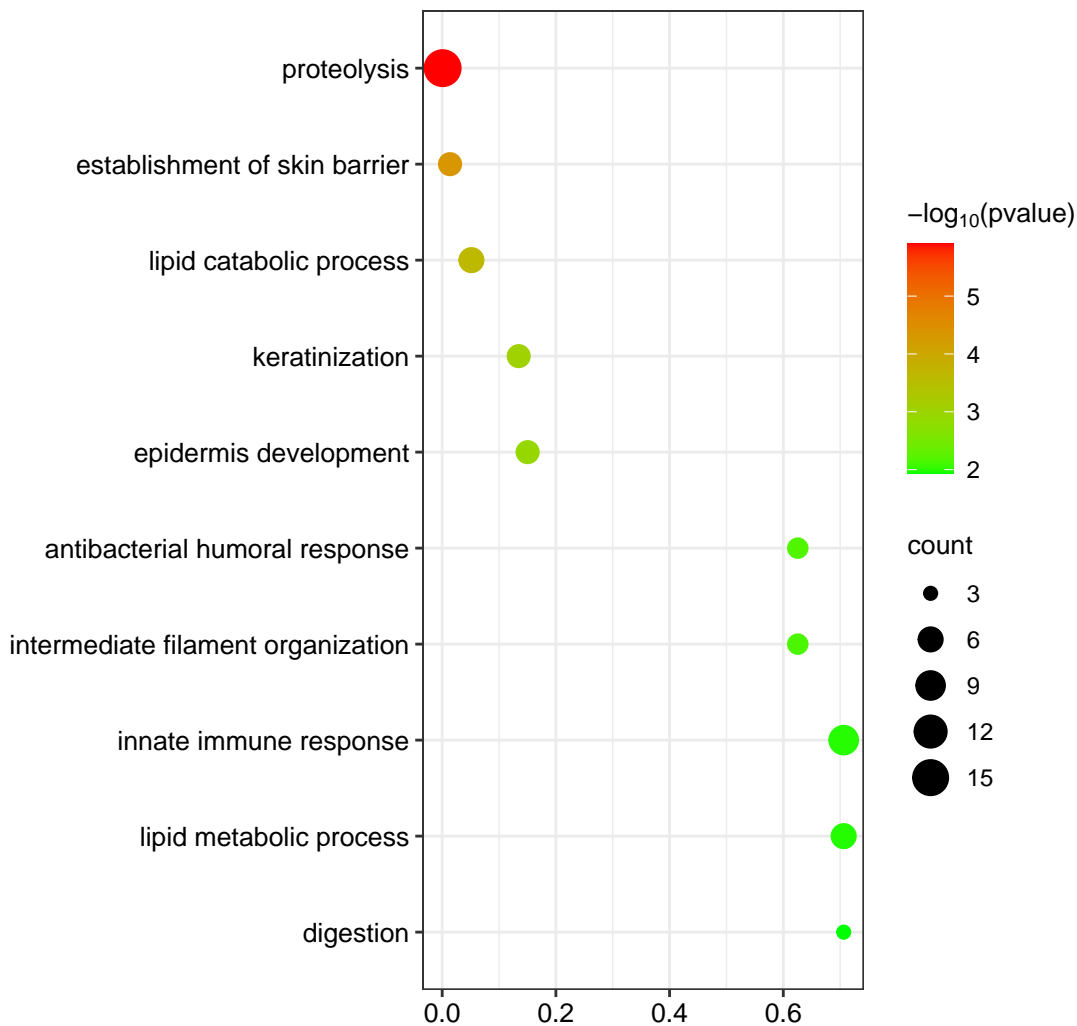

Supplement: Supplemental Information 10 — PZF/PZFX files must be opened using GraphPad Prism. [file peerj-13-19517-s010.zip › FIG 4B-F/FIG 4D/GO_BP.pdf]

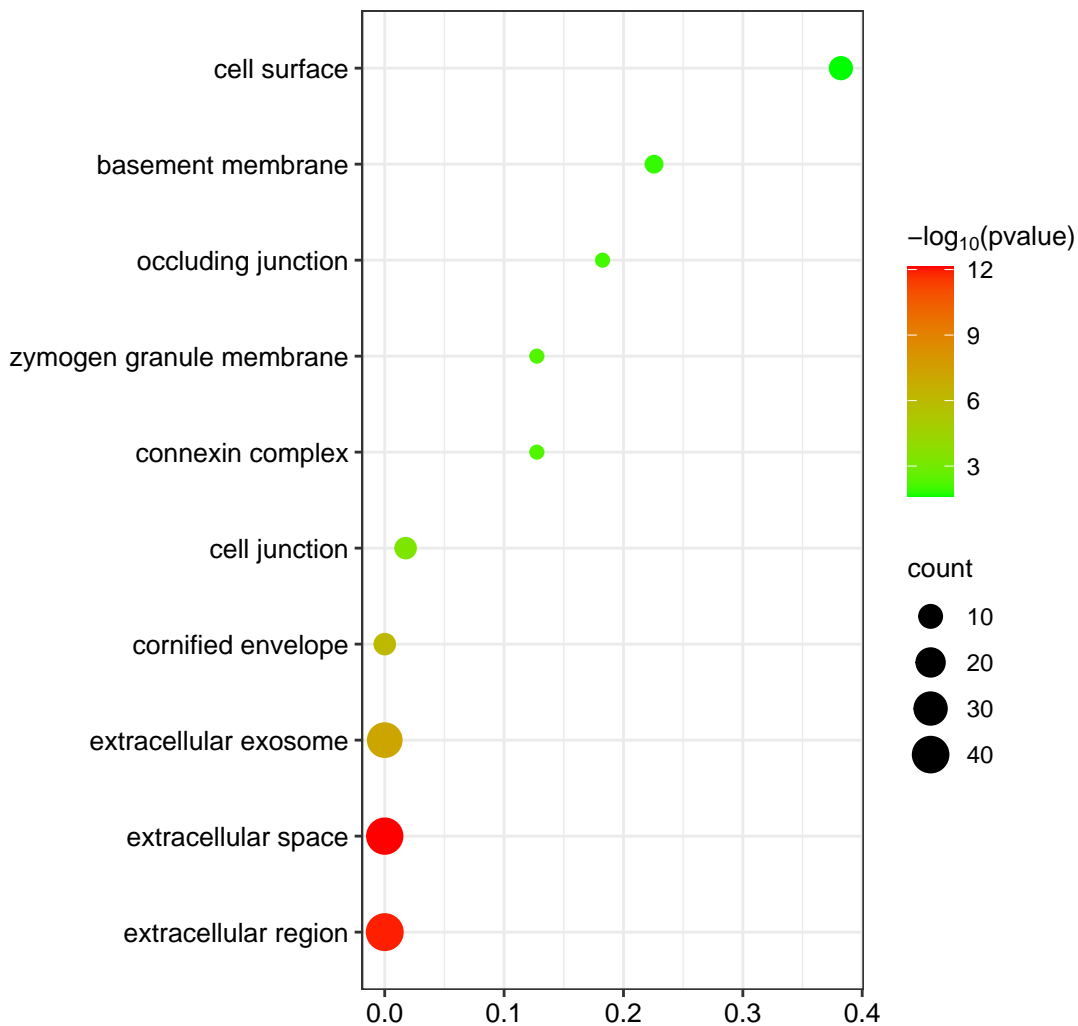

Supplement: Supplemental Information 10 — PZF/PZFX files must be opened using GraphPad Prism. [file peerj-13-19517-s010.zip › FIG 4B-F/FIG 4E/GO_CC.pdf]

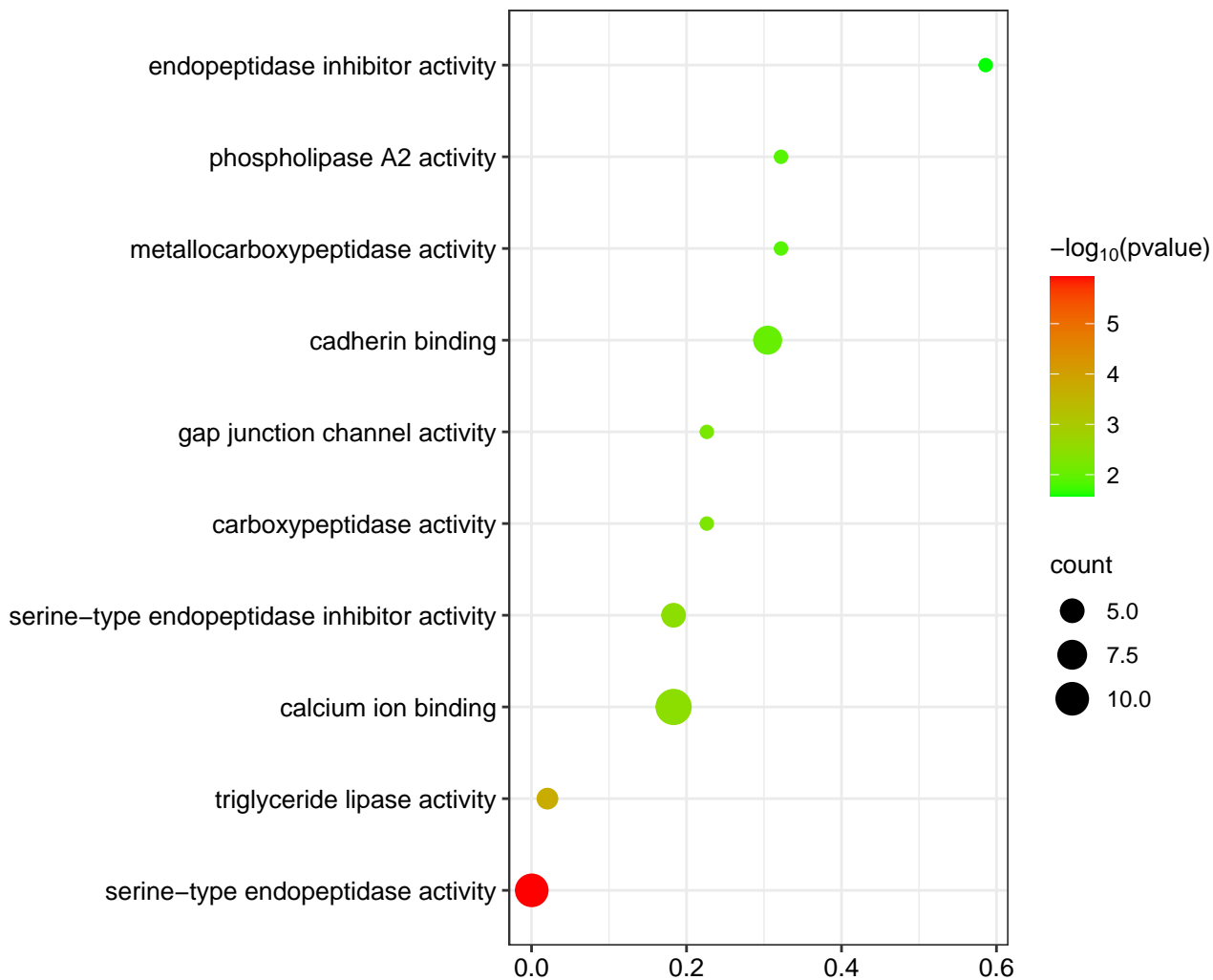

Supplement: Supplemental Information 10 — PZF/PZFX files must be opened using GraphPad Prism. [file peerj-13-19517-s010.zip › FIG 4B-F/FIG 4F/GO_MF.pdf]
