# Supplementary material for: PLA2G16 expression predicts prognosis and gemcitabine sensitivity in patients with pancreatic cancer
Source: PeerJ. 2025 May 30;13:e19517. doi: 10.7717/peerj.19517 (PMC12129006; doi:10.7717/peerj.19517)
Supplement: Supplemental Information 3 — PZF/PZFX files must be opened using GraphPad Prism. [file FIG 1C.pdf › FIG 1/FIG 1C.pdf]

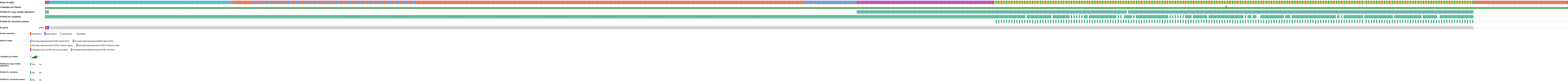

Study of origin
Samples per Patient
Profiled for copy number alterations
Profiled for mutations
Profiled for structural variants
PLAAT3
0.3%*
Genetic Alteration
Amplification
Deep Deletion
No alterations
Not profiled
Study of origin
Pancreatic Adenocarcinoma (ICGC, Nature 2012)
Pancreatic Adenocarcinoma (QCMG, Nature 2016)
Pancreatic Adenocarcinoma (TCGA, Firehose Legacy)
Pancreatic Adenocarcinoma (TCGA, PanCancer Atlas)
Pancreatic Cancer (UTSW, Nat Commun 2015)
Pancreatic Ductal Adenocarcinoma (CPTAC, Cell 2021)
Samples per Patient
0
2
Profiled for copy number alterations
Yes
No
Profiled for mutations
Yes
No
Profiled for structural variants
Yes
No
